# The Role of Funding and Policies on Innovation in Cancer Drug Development

**DOI:** 10.3332/ecancer.2010.164

**Published:** 2010-02-03

**Authors:** P Kanavos, R Sullivan, G Lewison, W Schurer, S Eckhouse, Z Vlachopioti

**Affiliations:** 1 LSE Health, Department of Social Policy, London School of Economics; 2,5 European Cancer Research Managers Forum (ECRM); 3 Evaluametrics Ltd; 4 LSE Health, London School of Economics; 6 LSE Health, London School of Economics

During the past two decades, cancer incidence has steadily increased due to aging populations, lifestyle and environmental factors, with great personal and national economic consequences. Concurrently, cancer treatments have improved with increased treatment options as well as lengthier disease and disease-free survival rates. The latest innovation in cancer treatments are targeted biological treatments, joining the current arsenal of surgery, radiotherapy and chemotherapy, particularly significant in latter stage cancers associated with very poor survival.

Despite this latest breakthrough in cancer treatment, this has in fact only opened the door to beginning to understand the complexity of cancer on a molecular and genetic basis. Oncology research and development (R&D) has the highest failure rate for new molecular entities (NME) and significantly higher development costs. Although tremendous scientific and economic barriers exist, the oncology development market has increased twofold over the past 5 years.

This report aims to map current oncology R&D funding and management, primarily in Europe and the USA, to examine public-private relationships, current oncology R&D strategies and oncology innovation policies. Its objectives are:

- to map current funding and management of oncology R&D via questionnaire surveys and interviews of oncology experts;
- to produce a high-resolution bibliometric analysis of oncology drug R&D in order to better understand the public-private mix in research activity;
- to investigate the cumulative life-time funding of specific oncology drugs;
- to review current public policy affecting oncology drug R&D, specifically, public R&D investment policies, transnational investment policies, regulatory policies and drug reimbursement policies; and
- to propose future oncology policies supporting the R&D process.

## Results: Funding, Bibliometric Outputs and Faculty Survey

### Funding

Public oncology R&D funding can be sourced from a variety of sources: national governments, regional authorities, charities, non-governmental organisations and supranational organisations. Funding can be ***directly*** tagged for oncology research from these organisations or ***indirectly*** flow into oncology research via overall budgets (i.e. hospital budgets).

Our examination of oncology funding found 153 public research funding organisations (RFO) in the EU (UK 19, France 12, Belgium 12, Italy 11) and 21 in the USA who spent greater than €1 million annually. The EU RFOs collectively spent €2.79 billion and the US RFOs €5.8 billion, although the EU did not include European Commission (EC) investment, which is significant and likely brings the EU figure closer to €3 billion. Individually, the US and the UK (€1.1 billion) were the largest oncology public R&D investors, whilst Germany (€426 million), France (€389 million) and Italy (€233 million) followed. Calculations per capita found leaders (USA, UK) unchanged, however, placed Sweden, the Netherlands and Norway next. Likewise, examination of public oncology drug R&D investment placed the USA (€1.67 billion) and UK (€305 million) at the top, regardless of absolute or per capita valuation. When direct and indirect funding are added together, the EU invests 0.011% of GDP, or €3.64 per capita, and the USA 0.018% GDP, or €5.74 per capita. Furthermore, the EU has significantly increased funding by 34.7% from 2004 to 2007 whilst the USA increased only 9.7%.

Examination of national cancer strategies and funding found only the United States and United Kingdom with strong visions and policies, whilst the remaining EU countries appear to favour an *ad hoc* approach. Philanthropic oncology remains impressive, estimated at over €500 million Europe and €230 million in the USA in 2007. Private oncology investment by the top 17 pharmaceutical companies globally in 2004 amounted to €3.1 billion, 59% from European companies. In addition, public-private partnerships (PPP) are becoming more common and found in 68% in the United States, 57% in the EU and 31% elsewhere of new oncology drug R&D projects.

### Bibliometric analysis

Bibliometric analysis of 19 anti-cancer drug publications (1963–2009) produced 28,752 papers for analysis. Paper outputs rose from 200 annually in 1980 to 2000 by 2007–2008. Examination of 15 main oncology research countries found the USA the leader (33%) followed by Japan (10.6%), Italy (7.5%) and the UK (7.1%). Initially, the USA and Europe dominated oncology research outputs, although recently other counties such as China and India are increasing their publication outputs.

Neighbouring countries still favour each other (USA: Canada, UK: NL) despite increasing international collaboration. Further, countries appear to concentrate on certain drugs and produce less research on others. Surprisingly, most national oncology research portfolios were poorly correlated with their internal oncology burden.

The type of oncology research performed changed with time from basic to clinical, although per drug this was not necessarily the case. Different countries produced different types of research (i.e. basic: India, China; clinical: Spain, Greece), with 15% of papers describing phased clinical trials, primarily Phase II.

The presence of 26 leading pharma companies, including the 12 associated with development of the 19 selected drugs, occurred in 1589 papers, or 5.5% of the total. Dominating companies responsible for oncology paper outputs were Aventis (274 papers), AstraZeneca (173) and BristolMyerSquibb (155).

### Survey of oncology faculty

Faculty were surveyed on a number of public and private oncology R&D issues. They felt strongly that PPP were important for future oncology developments, however, its ideal definition was not clearly defined regarding financial incentives and length of private support. Europeans were less agreeable regarding oncology R&D nationalisation than Americans and Canadians, whilst American faculty felt reimbursement policies for new oncology drugs was less important to future successes. All agreed, however, that the degree of national public sector investment was inadequate to meet future oncology demands.

Faculty expressed concern about the inadequacy of current oncology R&D models and encouraged re-thinking of ideal models. Suggestions included greater transnational cooperation, support of translational research and a degree of institutional involvement. Specifically, regulatory bottlenecks must be resolved as well as ideal balance of public versus private funding.

## Policy Implications: Funding, Bibliometric Outputs and Faculty Survey

Our funding analysis produced a number of interesting issues. First, it appears there are funding gaps between the USA and Europe, supplemented by further variations within Europe. Second, it appears public funding is more likely to support basic rather than applied research, whilst industry supports the latter. Third, European funding appears to be fragmented concurrently with duplication and inadequacies. Fourth, indirect and philanthropic funding appear to be significant and uncounted sources of oncology funding. Fifth, PPP investment in oncology is of increasing importance in addition to being complex, reducing economic risk, smoothing the operations process and will likely play an increasing role in the future.

Our survey of oncology faculty found substantial support for PPP although its ideal definition remained unresolved. Both public and private sources of activity and funding are important to oncology, yet the balanced equation of their interaction and involvement needs further study. New models specifically for oncology R&D are urgently needed to reduce attrition rates, increase the rate and sophistication of parallel biomarker development and work on the vast number of combination regimens and indications necessary for the next generation of cancer drugs.

New PPP policy development should include a number of new variables.

- Strong institutional support and dedicated public RFO funding.
- Increased freedom to operate for translational leads within specific projects, achieved by improved support, light-touch governance and decreased administrative bureaucracy (national legislative, private-contractual, public contractual).
- Partnerships supporting transnational cooperation and collaboration focused on key cancers, including ‘orphans’ not viewed as commercially attractive.
- Partnerships subject to high-quality peer review and fully disclosable upon completion to the public.

Faculty clearly identified over-regulation and reimbursement of new cancer drugs as critical issues, which continues to overshadow public sector oncology R&D and remains a threat to future new breakthroughs. Of further significance was intellectual environment and infrastructure for oncology R&D, expressed as vital to institutional and national policies. Strategic alliances and cooperation between industry and academia are key to future oncology discoveries, as the complex nature of oncology research cannot support monopoly in knowledge and creation. Particularly for novel biologicals this holds true

### Fostering Oncology Innovation

Encouraging innovation in oncology brings forth a number of priorities: first, the role of science, research and innovation; second, the role of pricing and reimbursement systems; third, the continuous evaluation of oncology drugs; fourth, the ideal environment for long-term innovation and fifth, the optimisation of resource allocation in health care.

## National and Supranational Roles in Innovation

Governments play an important role in encouraging and fostering innovation, including direct governance for key research areas and indirect mechanisms including taxation. Governments understand this encouragement has direct economic consequences as well as social benefits, which exceed private benefits. Collaboration between public and private enterprises further spreads benefit and ensure greater likelihood of success.

Despite this recognition, the complex nature of oncology requires both direct and indirect measures. Using only prescriptive and coercive regulations may be cumbersome, expensive and inefficient, whilst output- or performance-based regulations may have more likelihood of success. Tax incentives via R&D credits may be targeted to serve specific objectives, whilst enhanced market exclusivity periods may encourage intellectual creativity. Particularly in oncology research each player only has a portion the knowledge required for presenting new solutions, leading to ideally open access requirements. This presents the need for new model developments in oncology R&D to encourage innovation, leading to new treatments more quickly.

In Europe, the EC has recently taken steps to encourage innovation by promoting translational and transnational research, in addition to PPP, in the hopes that cooperation will prove stronger than its current fragmentation. Although not all European countries have cancer strategies in place, particularly newer members, there is focused application to improve oncology treatments and to encourage development of new ones. Despite this attention, there continues to be room for improvement in European oncology R&D. Cancer charities are a significant yet neglected source of oncology funding, their fragmentation and duplication continues to be mirrored by many national oncology organisations. Furthermore, some oncology research may not be funded due to precisely its specialisation and innovation, such as very specialised basic cellular research found in only few countries, as it does not qualify for translational or transnational funding.

In America, cancer research is less fragmented due to the umbrella organisation of the National Cancer Institute, which supports both molecular and translational research as well as increasingly encouraging PPP. However, it does suffer from state-level and indirect fragmentation (i.e. hospital research budgets), and its level of charitable oncology R&D funding is less than the EU.

Globally, it appears translational cancer research is still in its infancy, only recent programmes giving focus and direction. Likewise, PPP have room for growth and direction both in Europe and the US – which should be seen as a unique opportunity at these cross roads. Fragmentation continues, particularly at charitable level, with some negative consequences for administration costs and research duplication, but perhaps benefiting highly specialised research areas still in experimental stages.

## The Uniqueness of Rare Cancers

Rare cancers represent approximately 20% of oncology cases, including childhood cancers, each with variations in incidence, mortality and survival rates. This variability is mirrored between EU members with regards to treatment access, information availability and medical expertise. These factors present rare cancers as a unique case, requiring multidimensional action to encourage R&D, access and uptake of new treatments. Such actions include re-organising regulations, encouraging R&D through collaboration, creating consensus guidelines on multidisciplinary treatment, addressing patient treatment access, as well as improving information access for patients and health care professionals.

## Role of the Reimbursement System

Over the past decade, health care costs have increased, including drug spending although only accountable for 10%–20% of total care costs. Management of drug spending is important, particularly as regressive management may cause access, equity and health outcome issues. Appropriate pricing and reimbursement can help manage health care costs whilst concurrently encouraging innovation in R&D and treatment. A number of criteria can help achieve these goals.

First, timely treatment access is paramount, particularly for innovative drugs, and encouraged through ‘fast track’ approval and reimbursement procedures (e.g. FDA fast track process for priority drugs). Conditional reimbursement and pricing, where access is ensured whilst ‘real-world’ data collection continues, as well as physician flexibility in prescribing can further aid access and encourage innovation.

Second, reimbursement based on values, including explicit and objective assessments, is important to consider. This value should consider both societal and individual value and include comparisons to current best practice. Third, reimbursement and pricing policies should contain some degree of flexibility, where levels are adjusted as new data become available.

Fifth, collaboration should be encouraged between payers, providers and manufacturers to explore new pragmatic ways of delivering innovative value. Sixth, standard guidelines to assess drug benefits should include humanistic and patient-focused benefits such as quality of life (QoL), longer term direct cost offsets, indirect system costs and caregiver and patient benefits.

## Risk Sharing

Traditionally, payers absorb all risks associated with purchasing new medical technologies. Risk sharing attempts to redistribute the risk balance between payer and technology supplier, typically involving the supplier to provide a ‘guarantee’ relating to outcome. These outcomes could include clinical parameters, QoL, resource usage, (d) financial and economic outcomes. Although new in health care, this method is likely to gain use in the future due to total cost issues, first, for admitting new treatments onto national formularies and, second, to enabling faster uptake. In oncology in particular, this could be interesting due to limited patients carrying the same genetic tumour codes.

## Continuous Evaluation of Oncology Drugs

Ex-ante evidence is currently required to present evidence for approval and reimbursement decisions, however, sole reliance on this method ignores evidence outside the clinical phase environment. Ex-post evidence is just as important, however, in proving value of new treatments yet is widely ignored. Collection and evaluation of such data is costly and perhaps should be shared between private and public interests, yet is imperative in oncology with its heterogeneous patients.

## Optimising Resource Allocation in Health Care

Although resources are allocated mechanistically in health care, this does not guarantee optimal use – in fact evidence suggests that many health systems have room for improvement including oncology. Demand-side behaviours by both clinicians and patients, real-time information systems for payers and providers as well as system and policy performances all must be considered. Savings emerging should be re-allocated and re-invested to improve patients’ quality of care and health services.

## Conclusions

The report shows oncology R&D and treatment are on the brink of a new era, providing a unique opportunity now to redirect and refocus national, supra-national as well as regional policies and procedures that may impact oncology directly or indirectly. New models for PPP must be created, giving credence to both public and private ownership within complex and often unique diseases including cancer. Reimbursement decisions are important and can greatly impact future oncology innovation and investment and must be carefully considered prior to implementation (and monitored closely thereafter). Pricing should consider innovation and value, not just with macro-societal views but also consider micro-individual patients. The overall goal is improved patient outcomes and survival, and for oncology this means collective operation and collaboration.

## Figures and Tables

**Figure 1.1:**
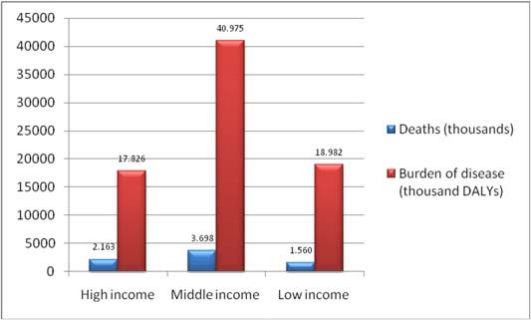
Cancer related deaths and burden of disease grouped by income per capita (2004) *Source:* [4].

**Figure 1.2:**
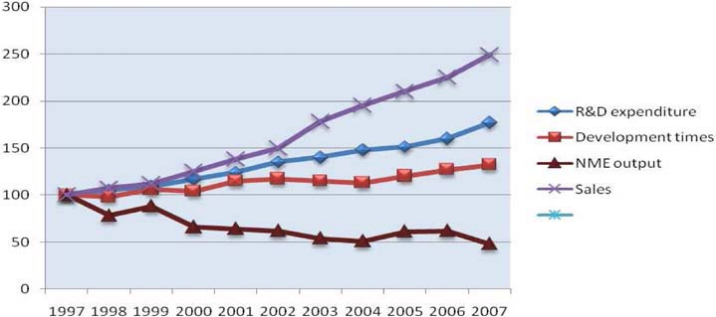
**Global R&D expenditure, development times, NME output and global pharmaceutical sales (1997–2007)** *Notes:* Each trend line has been indexed to 1997 values Development time data point for 2007 includes data from 2006 and 2007 only ***Source:*** CMR International and IMS Health

**Figure 1.3:**
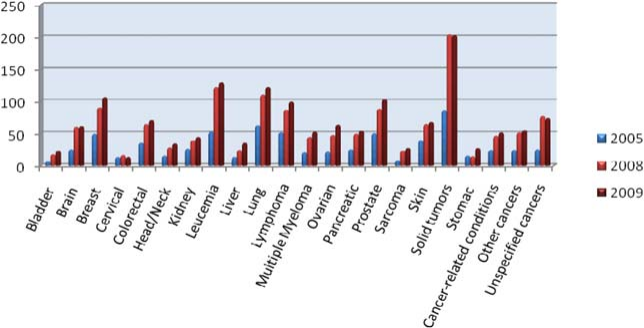
**Number of new cancer drugs in development by type of cancer.** *Note:* Some drugs are listed in more than one category. ***Source:*** PhRMA (‘New medicines in development for cancer’).

**Table 1.1:**
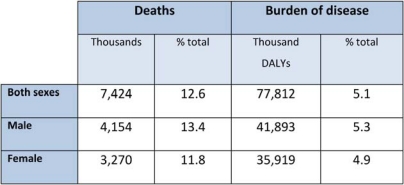
Global cancer related deaths and burden of disease by sex (2004)

*Source:* [4].

## List of Abbreviations

ABPI: Association of British Pharmaceutical Industry (UK)
ACI: Actual citation impact
ACS: American Cancer Society (USA)
AHCPR: Agency for Healthcare Research and Quality (USA)
AICR: American Institute for Cancer Research (USA)
ASMR: Amelioration du Service Medical Rendu (France)
BBSRC: Biotechnology and Biological Sciences Research Council (UK)
BCG: Bacillus Calmette-Guerin
CCLG: Children’s Cancer and Leukaemia Group
CCRA: Canadian Cancer Research Alliance (Canada)
CDC: Centers for Disease Control and Prevention (USA)
CML: Chronic myeloid leukaemia
CMR: Centre for Medicines Research
CRADA: Cooperative research and development agreements
CRUK: Cancer Research UK (UK)
CSC: Cancer stem cells
CSO: Common Scientific Outcome
DALYs: Disability adjusted life years
DC: Dendritic cell
DCE-MRI: Magnetic resonance imaging
DHHS: Department of Health and Human Services Agencies (USA)
DNA: Deoxyribonucleic acid
DOD: Department of Defense (USA)
DOE: Department of Energy (USA)
DoH: Department of Health (UK)
EC: European Commission
ECMC: Experimental Cancer Medicine Centres
EDCTP: European and Developing Countries Clinical Trials Partnership
EFPIA: European Federation of Pharmaceutical Industries and Associations
EFRR: Epidermal growth factor receptor
EFTA: European Free Trade Association
EMEA: European Medicines Agency
EPA: Environmental Protection Agency (USA)
ESRC: Economic and Social Research Council (UK)
EU: European Union
FDA: US Food and Drug Administration (USA)
FECS: Federation of European Cancer Societies
FP: Framework Programme
GAVI: Global Alliance for Vaccines and Immunisations
GDP: Gross Domestic Product
GFATM: Global Fund to Fight AIDS, Tuberculosis and Malaria
GMP: Good manufacturing process
HBV: Hepatitis B
HCFA: Health Care Financing Administration (USA)
HER2: Human epidermal growth factor receptor 2
HIV: Human immunodeficiency virus
HPV: Human papilloma virus
ICR: Initial Cancer Research Partners
ICRP: International Cancer Research Portfolio
IFN-α/γ: Interferon, alpha/gamma
IL-2: Interleukin-2
IMI: International Medicines Initiative
INCA: Institut National du Cancer (France)
IOM: Institute of Medicine
IOM: Institute of Medicine (USA)
IPR: Intellectual property rights
IPR: Intellectual property rights
ITCC: Innovative Therapies for Children with Cancer
JPMA: Japan Pharmaceutical Manufacturers Association
MA: Market Authorisation
MAb: Monoclonal antibodies
MDR: Multi-drug resistance
MEPS: US Medical Expenditure Panel (USA)
MRC: Medical Research Council (UK)
NASA: National Agency for Space Exploration (USA)
NCE: New chemical entity
NCI: National Cancer Institute (USA)
NCRI: National Cancer Research Institute (UK)
NDA: New drug applications
NGO: Non governmental organisations
NHS: National Health Service (UK)
NIH: National Institute of Health (NIH)
NK: Natural killer
NME: New molecular entity
NSCLC: Non-small cell lung cancer
NSF: National Science Foundation (USA)
P CG: % of papers on clinical guidelines
P GOV: % of papers on government policy
P MED: % of papers in mass media
P TB: % of papers in text-books
PCI: Potential citation impact
PET: Positron emission tomography
PhRMA: Pharmaceutical Research and Manufacturers of America
PIP: Paediatric investigation plan
PR: % of reviews
QA: Quality assurance
QC: Quality control
R&D: Research and Development
RaDiUS: RAND Corporation’s Research and Development in the US database
RFO: Research funding organisations
RL j: Research level journals
RL p: Research level titles
RNA: Ribonucleic acid
ROI: Return on investment
RoW: Rest of world
SENDO: Southern Europe New Drug Organisation
SFOP: French Society of Paediatric Oncology
SIOPE: International Society of Paediatric Oncology
SmPC: Summary of Product Characteristics
SPC: Supplementary protection certificate
SSRI: Serotonin re-uptake inhibitors
SWOT: Strengths, Weaknesses, Opportunities and Threats
TCA: Tricyclic anti-depressants
TILs: Tumour infiltrating lymphocytes
TNF-Î±: Tumour necrosis factor, alpha
TRAP: Target related affinity profiling
UJ: Utility of journals
USA: United States of America
USDA: US Department of Agriculture (USA)
USTPO: US Patent and Trademark Office (USA)
VA: Department for Veterns’ Affairs (USA)
VEGF: Vascular endothelial growth factor
WHO: World Health Organisation
WoS: Web of Science
