# Supplementary material for: The Role of Funding and Policies on Innovation in Cancer Drug Development
Source: Ecancermedicalscience. 2010 Feb 3;4:164. doi: 10.3332/ecancer.2010.164 (PMC3234026; doi:10.3332/ecancer.2010.164)
Supplement: Related texts

# Sub article: 1. Background and Objectives

**Published:** 2010-02-03

## 1. 1. The Burden of Cancer

### 1.1.1. Risk factors, incidence and mortality

The aging of population and lifestyle factors such as obesity, physical inactivity, alcohol consumption, rising number of female smokers and lower rates reproduction, along with genetic susceptibility are among the most important underlying reasons for the increasing cancer incidence in industrialised nations [1,2]. However, the burden of cancer is no longer limited to developed countries. On top of the growing risks of poor diet, tobacco, alcohol and industrial exposures, the less developed world is already burdened with cancers related with infectious agents [3] such as *Helicobacter pylori*, human immunodeficiency virus (HIV), hepatitis B (HBV), human papilloma virus (HPV) and others [2]. Even if the total burden of cancer remains highest in wealthy countries, developing countries are closing the gap rapidly.

Advances in diagnostic methods, surgical techniques, radiotherapy, innovative vaccines and drug treatments have contributed to improved outcomes, particularly for patients suffering from the most ordinary cancers such as prostate, breast, colorectal and, more recently, lung. Thus, mortality rates have stabilised in some populations (e.g. Europe) [1].

Still, new cancer cases were estimated at 11.47 million worldwide in 2004, whilst cancer accounted for 7.42 million deaths that same year (Table 1.1, Figure 1.1) [4]. These figures could reach 27 million cases by 2030 with 16 million deaths [5], making cancer as cause of death the fastest increasing rate globally, and in some countries already the primary cause of adult mortality (the United Kingdom, the Netherlands) [4]. In most high-income countries, cancer is the second highest common cause of death after cardiovascular disease, with lung, colorectal, breast and stomach cancers together accounting for 13% of total mortality in 2004 [4].

### 1.1.2. Prevalence and direct/indirect costs

Cancer prevalence refers to the burden of disease in a population and is associated with the survival of cancer patients. In terms of total disease burden, malignant neoplasms accounted for 14.6%, 7.2% and 2.3% of disability adjusted life years (DALYs) (‘healthy’ years lost) in high-, middle- and low-income countries, respectively, in 2004 (4) (Table 1.1, Figure 1.1). In the EU25 and the USA, cancer ranks third behind mental and cardiovascular disease in relation to DALYs lost whilst in other industrialised countries such as Australia, Japan and New Zealand, cancer ranks second relative to DALYs lost, following mental illnesses (1).

Despite the rising burden that cancer poses, health spending related to treatment of cancer patients does not reflect this trend. Based on 2004 total health expenditure figures from OECD Health Data, cancer care seems to account approximately for 6.6% (on average) of total direct health care expenditures in most developed countries [1]. Medical treatments for cancer account for 10%-20% of cancer expenditure – primarily as inpatient hospital care – and 5% of total pharmaceutical expenditures [1].

Indirect costs associated with inability to work account for a large proportion of the total cost that cancer imposes on society. Relevant studies demonstrate that indirect costs range between 65%–85% of total costs [6–8].

### 1.1.3. Advancement in cancer medical treatments

Various treatment methods exist today for cancer, including surgery, classical chemotherapy (i.e. agents that inhibit cancer growth such as alkylating agents and anti-metabolics [9,10]), radiotherapy and an increasing number of ‘targeted’ drugs against hormone, and growth factor receptors as well as cell-signalling pathways.

Cancer drugs are often introduced into clinical management in late-stage patients [1]. Efficacy in early-stage disease often translates to greater success rates when the drug is combined with surgery and/or radiotherapy [1]. Multiple drug regimens are the backbone of treatment and the newer generation of cancer drugs promise reductions in toxicity, improved tolerability and, in the case of orally delivered medicines, economic benefits and increased patient satisfaction by out-of-hospital and in-community treatment delivery.

The analysis of tumour gene/protein expression profiles, as well as other technologies such as circulating cancer cells, has driven the ‘translational’ science of prognostic and predictive biomarkers. In the latter case, such markers can help predict whether a tumour is likely to respond to a certain treatment, the so-called personalised medicine. However, progress in genomics and proteomics has also revealed that most tumours are in practice genetically unique and highly complex. Laboratory and clinical development of these new biomarkers along with the next generation of cancer drugs is extraordinarily complex. As a result, the already costly and timely research and development (R&D) process in cancer drug development becomes even more challenging.

## 1.2. The R&D Process

### 1.2.1. General trends

Recent advances in genomics, proteomics and computational power present new ways of understanding the inner workings of human disease at molecular level, making discovering and developing safe and effective drugs challenging as well as promising.

Scientists in government, academic, not-for-profit research institutions and the pharmaceutical sector contribute to basic research in order to understand the disease and choose a target molecule. In general, it takes about 10–15 years to develop one new medicine from the time of discovery to when it is available for treating patients. Moreover, substantial research has been carried out on estimating the costs of drug development either generally [11] or according to therapeutic area [12], and, although there is controversy around the use of single numbers [13], it is clear that it takes a large, lengthy effort to get one medicine to patients. In 2005, the average cost of developing drugs against cancer was estimated to be 20% higher (€964 million) than the mean cost of developing a new molecular entity (NME) (€803 million) [11]. This number incorporates the cost of failure: For every 5000–10,000 compounds that enter the R&D pipeline, ultimately only one receives approval [14].

### 1.2.2. Cancer R&D

Over 50 years ago when cancer was described for the first time as a genetic disease, hopes for early diagnosis and targeted treatments rose. However, the progress in genomic technologies and fundamental cancer biology has unravelled a complexity among cancers practically making each tumour’s genetic fingerprint unique [15]. Therefore, it is of no surprise that oncology R&D has the lowest success rate (and, by implication, the highest cost) of any therapeutic area in the pharmaceutical discovery and development, making the R&D process even more challenging for a number of reasons. Indeed, in the case of cancer, when a molecule enters clinical trials, there is only a 5% probability that it will turn out to be a commercially viable product [16].

There are further R&D issues unique to oncology. First, instead of healthy volunteers, patient populations who have practically failed all other treatments participate in Phase I clinical trials. This imposes a major burden on the assessment of the safety and efficacy of the compound, as well as the identification of relevant biomarkers. A Phase II trial, where specific cancer types are being selected and dosage is determined, is often more enlightening. Second, contrasting most diseases, cancer is a set of proliferative diseases representing a variety of different specific conditions. Third, there are huge differences among cancer patients due to the unique genetic fingerprints that almost any tumour has, making inter-patient heterogeneity a major challenge. Finally, even if the drug makes it to Phase III, cancer patients are normally treated with multiple drugs at the same time, thus the standard of care has to be adapted and enhanced to add the new drug to it. Perhaps, it is not surprising that cancer drugs often fail in Phase III, which is the most costly part of drug development programme.

The current knowledge of the biology of cancer targets and their significance in the disease process is undoubtedly deficient and major problems remain in how to deliver many anti-cancer agents that *in vitro* are effective. What seems to become increasingly likely is that there will be a shift from defining a cancer by site [17–19] (i.e. malignancies originating from the same organ system are grouped together as one single disease, receiving basically the same treatment) to identifying similar therapeutic target cancers that, regardless of whether they arise in the same locations or not, share alterations of the same genes. Thus, successful cancer drug discovery will require, apart from finding the best medicine for a certain target, also identifying the patient population whose tumours actually carry the relevant genetic alteration, leading to both individualised diagnosis and personalised therapy.

The issue arising here is that treating cancer as a collection of ‘orphan diseases’ could lead to the creation of smaller markets, inapt to the traditional ‘best-seller’ model of discovering and developing new cancer drugs [20]. Indeed, some pharmaceutical companies have been reluctant to invest in early basic science and pre-clinical research and development, given the limited revenue prospects of such a business model.

In the end, it seems that the overabundance of these potential targets are major scientific hurdles to issues of drug delivery, target appraisal and confirmations and potential medicines manufacturing and effectiveness enhancement. Furthermore, the development of new therapeutic strategies is too much for industry, government or universities to do separately. Collaboration appears to be the key to facilitate the discovery and development of effective new cancer drugs and optimise their application.

## 1.3. Trends in R&D Spending and Output by the Private Sector

### 1.3.1 Aggregate R&D trends in pharmaceutical and biotechnology industries

Whilst total pharmaceutical R&D expenditure^a^ has increased and sales^b^ have grown, revenue keeps on relying on a small number of molecules reflecting an ongoing drop in productivity (Figure 1.2).

R&D costs continue to rise with development, now corresponding to approximately one third of all spending. Development times also continue to grow during all phases of development. Discovery and regulatory times have not marked any significant change during the past 5 years and are not expected to do so in the years to come [21]. Still, there is an obvious trend in some companies to invest more in early development in order to avoid the massive cost of late-phase failure.

On the other hand, success rates are not improving as only 20% of molecules entering Phase II will be marketed. Biotechnology-derived and self-originated substances do have, however, a slightly better chance of success relative to chemical and in-licensed entities [19]. Consequently, the number of NME launches dipped to a new 20 year low in 2007, despite the encouraging signs in 2005. In fact, during the 1997–2007 period, the introduction of NME dropped by 50%. Yet, biotechnology products have accounted for almost a quarter of all NME launches [19].

The molecules currently in development are mainly looking at therapeutic areas with high value and significant level of unmet medical need [13,20]. The underlying rationale is that although molecules with a new action mode are associated with a significant success risk, at the same time they offer the greatest opportunity for innovative and high-value medicinal products [22].

### 1.3.2. Cancer R&D trends

As previously been described, R&D in the area of oncology is particularly challenging. However, oncology-related R&D is booming, and this is shown by the enormous share of oncology compounds in the pharmaceutical sector’s clinical pipeline. In the USA alone, there are more than 800 new compounds in development for cancer in 2009, compared with 750 in 2008 and just under 400 in 2005^c^ (Figure 1.3).

This reality reflects the focus of R&D investments towards therapeutic areas and technologies of greatest opportunity (perceived as a combination of low-generic penetration and high price levels), associated with the highest unmet medical need. Hence, the opportunity associated with a potential success cancels out the fact that attrition rates among cancer products are as high as 95%.

## 1.4. Aim of This Report

The global organisation and funding of cancer research follows many different models. The global flow of knowledge, innovation, research and development has dramatically increased the complexity of cancer research. Since the mid-1950s cancer drug development has become the dominant area of cancer research. The clinical need to find drugs to prevent cancer, suppress recurrences (adjuvant), downstage tumours for surgery (neoadjuvant), treat metastatic disease and palliate has driven the development of new molecular entities, be they chemical or biological.

However, the vast complexity of this nexus coupled to the widely different paradigms that appear to operate across Europe, USA and the Far East do not easily lend themselves to strategic analysis. In particular questions arise as to:

- what models of funding and management have, or have not been successful;
- what is the most efficient, creative and innovative model for public-industry cooperation and collaboration; and
- what policies should we be developing to support drug development in cancer, and to whom should these be directed (government, NGOs, etc).

In this context, the aim of this report is to map out the current funding and management structures for cancer drug R&D in Europe and the USA, with particular reference to the public-private interplay, and following a review of current strategies put forward recommendations to improve cancer drug innovation.

The report focuses on the USA, Germany, UK, Italy, France, the Netherlands, Sweden and Spain primarily, but also includes evidence from EU27 and Canada, where it is available. Although the main focus is on adults, the report includes paediatric oncology as drug development in this area is now a critical public policy issue.

The detailed objectives of the research presented in the report are fivefold:

First, to map current funding, peer review and management of cancer drug development. Through a mixture of questionnaire- and interview-based techniques, the current funding in oncology, peer review and management practices is mapped for the countries in question. This encompasses pre-clinical drug development, early phase clinical trials through to pivotal Phase III, although in the latter case this is assumed to be entirely within industry. The report covers both New Chemical Entities and Biologicals. This addresses the question as to who funds what and how. Within the institutional part of this mapping exercise the report drills down into the availability (or otherwise) of key platform technologies and infrastructure for the pre-clinical and clinical aspects.

Second, the report conducts and presents a high-resolution bibliometric analysis of outputs in drug development with a view to obtaining a better understanding of the public private mix in cancer research activity. Apart from early phase clinical trials where we know publications (outputs) do not reflect activity, the research presented in this report uses bibliometrics as a means of understanding current and past state of cancer drug development. The research uses major databases and constructs with key drug names to analyse the trends in output and impact by country, institution and even researcher. It examines how models of partnership (institution-to-institution and institution-industry) have changed and also at the patterns of funders over time. Such changes can then be reviewed in the context of the impact of national policies.

Third, to investigate the cumulative life-time funding of specific cancer drugs. The question of how and who funds cancer drug development can most effectively be answered by looking at the cumulative lifetime from inception/discovery (NME) to clinic. Taking a representative sample of current cancer drugs, the report looks at their funding histories from inception to market.

Fourth, to review the current public policy affecting cancer drug development. There is substantial literature on the generic process of drug development (essentially data documents) and specific policy issues (e.g. Intellectual Property) but few, which are either disease-specific or take a horizontal approach to the affect of policies (i.e. how different policies interact with each other in a cumulative manner). This report reviews five streams of current public policy, which affect cancer drug development both in Europe and the USA, notably (a) policies affecting public R&D investment in cancer research, particularly at Member State & Institutional (University-Hospital complex) level and differing polices around public-private ventures; (b) transnational investment polices focusing on funding by bodies such as the EU Commission compared to the US NCI; (c) regulatory policies, specifically the clinical trials directive; (d) tax and IPR policies and (e) the likely impact of drug reimbursement policies on the development of cancer drugs.

Finally, to propose polices to support further cancer drug R&D. This objective has been informed by the emerging evidence in this report as well as by key opinion leaders (senior clinicians) in cancer drug discovery and development with a view to proposing key public policy measures to support innovation in European cancer drug development.

Chapter 2 places cancer research in context by providing a historical background to cancer drug development; Chapter 3 presents the public and private trends in cancer drug research and development; Chapter 4 presents the results of the investigation into the cancer research activity in both the public and private sectors, whereas Chapter 5 builds on the senior clinician survey to propose policies to support cancer drug R&D. Chapter 6 debates the issues surrounding public policies affecting cancer drug development. Finally, Chapter 7 draws the main conclusions and considers the policy implications.

## References

[1] Jonsson B, Wilking N (2007). Annals Oncol.

[2] American Cancer Society (2009). Cancer Facts and Figures.

[3] Kanavos P (2006). The rising burden of cancer in the developing world. Annals Oncol.

[4] World Health Organisation (2008). The Global Burden of Disease: 2004 update.

[5] Boyle P, Levin B (2008). World Cancer Report 2008.

[6] World Health Organisation Priority medicines for Europe and the world.

[7] Brown M, Lipscomb J, Snyder C (2001). The burden of illness of cancer: economic cost and quality of life. Annu Rev Public Health.

[8] (2000). Disease-specific Estimates of Direct and Indirect Costs of Illness and NIH Support.

[9] Goodman LS, Wintrobe MM, Dameshek W (1946). Nitrogen mustard therapy. Use of methyl bis (B-chloroethyl) amine hydrochloride and tris (B-chloroethyl) amine hydrochloride for Hodgkin’s disease lymphosarcoma, leukemia, certain allied and miscellaneous disorders. JAMA.

[10] Farber S, Diamond LK, Mercer RD (1948). Temporary remissions in acute leukemia in children produced by folic acid antagonist, 4-aminopteroyl-glutamic acid (aminopterin). NEJM.

[11] DiMasi JA, Hansen RW, Grabowski HG (2003). The price of innovation: new estimates of drug development costs. J Health Econ.

[12] DiMasi JA (1995). R&D costs for new drugs by therapeutic category: a study of the US pharmaceutical industry. PharmacoEconomics.

[13] Adams CP, Brantner VV (2006). Estimating the cost of new drug development: is it really 802 million dollars?. Health Aff (Millwood).

[14] PhRMA (2007). Drug Discovery and Development: Understanding the R&D process Pharmaceutical Research and Manufacturers of America.

[15] Lengauer C, Diaz LA, Saha S (2005). Cancer drug discovery through collaboration. Nature Reviews, Drug Discovery.

[16] Clinton P (2007). **R&D Innovation: An Answer to Cancer (interview of Joseph Bolen** Millennium CSO and chairman of the 12th Annual World Congress on Drug Discovery and Development of Innovative Therapeutics in 2007). http://pharmexec.findpharma.com/pharmexec/PE+Features/RampD-Innovation-An-Answer-to-Cancer/ArticleStandard/Article/detail/463103.

[17] Bardelli A (2003). Mutational analysis of the tyrosine kinome in collateral cancers. Science.

[18] Lynch TJ (2004). Activating mutations in the epidermal growth factor receptor underlying responsiveness of non-small-cell lung cancer to gefitinib. NEJM.

[19] Paez JG (2004). EGFR mutations in lung cancer: correlation with clinical response to gefitinib therapy. Science.

[20] Booth B, Glassman R, Ma P (2003). Oncology’s trials. Nature Reviews. Drug Discovery.

[21] CMR International 2009 R&D compendium.

[22] Russell M, Gjaja M, Lawyer P (2008). The future of Pharma: adjusting the Pharma R&D Model. Boston Consulting Group, April 2008 in the PAREXEL’s Bio/Pharmaceutical R&D Statistical Sourcebook.

# Sub article: 2. The History and Science of Cancer Drug Development

**Published:** 2010-02-03

This chapter provides a brief journey through the major historical developments in European cancer research and a discussion of the key areas of current cancer drug discovery – new chemical entities and biologicals (immunotherapy).

## 2.1. An European History of Cancer Research

Europe’s seminal contributions to the milestones of cancer research are many and can be traced back to 1889 when Dr. Stephen Paget, a London Surgeon, developed the ‘seed and soil’ hypothesis of metastasis.

The prevailing view at that time was that cancer cells spread through the blood or lymph and could take up residence in any tissue. If this had been true, metastases would have shown a random distribution to other organs. Paget thought otherwise. ‘When a plant goes to seed, its seeds are carried in all directions. But they can only live and grow if they fall on congenial soil...’ he wrote (one of the wonderful things about research in this era was the use of the natural world as an unlimited source of metaphor and analogy, sadly lost in today’s prosaic research culture). Paget examined nearly a thousand cases and found that specific tumours metastasised consistently to particular organs.

Although this view was challenged by James Ewing, who gave his name to a type of soft tissue cancer, or sarcoma, claiming instead that metastases settled in the first organ they reached as they spread through the vasculature, Paget was to be proved correct in 1980 by Isaiah Fidler and Ian Hart working at the MD Anderson Cancer Center at the University of Texas.

Almost simultaneously with Paget in 1890, just a few years after the discovery of chromosomes, David P. Hansemann, a pathologist-in-training with Rudolph Virchow in Berlin, produced a theory of the pathogenesis of cancer. This included a key concept that the first change that occurs in cancer is an alteration of the hereditary material of a normal cell at the site where the cancerous process begins. In the process of linking cancer to chromosomal material, Hansemann coined the terms ‘anaplasia’^d^ and ‘dedifferentiation’.^e^ These terms have remained the basis of descriptive terms concerning the microscopic appearances of tumours ever since.

The great German tradition in cancer research continued with people such as Theodor Heinrich Boveri (1862–1915), a German zoologist. In his work with sea urchins, Boveri showed that it was necessary to have all chromosomes present in order for proper embryonic development to take place. His other discovery was the centrosome (1888), which he described as the special organ of cell division. He also reasoned that cancer begins with a single cell, in which the make-up of the chromosomes is scrambled, causing the cells to divide uncontrollably.

It was Paul Ehrlich who was to make the link between the immune system and cancer, suggesting that for the latter to survive the former had to be suppressed. Paul Ehrlich, who won the 1908 Nobel Prize in physiology and medicine, also predicted autoimmunity calling it ‘***horror autotoxicus’***. He coined the term ‘chemotherapy’ and popularised the concept of a ‘magic bullet’.

However, one should not view Europe’s role in turning back the tide of cancer as an isolated one. Then, as now, research was a complex dance over distance and time. Europe’s great contributions are intimately intermingled with those in other countries and continents.

Europe has also laid the foundations of many other domains of cancer research. The most important discovery in the history of cancer epidemiology was the carcinogenic effect of tobacco. The pivotal studies begun by Sir Austin Bradford Hill and Sir Richard Doll, and later with Sir Richard Peto, were to provide the springboard for five decades of research on both sides of the Atlantic.

In surgery there have been many seminal contributions by the European cancer research community. Umberto Veronesi, an Italian Surgeon and Oncologist, was the founder of breast-conserving surgery, inventing the technique of quadrantectomy, which challenged the idea, then dominant among surgeons, that cancers could be treated only with aggressive surgery.

Europe has been at the forefront of treating bowel cancer through surgical advances – from 1908 when Ernst Miles first described the abdominoperineal resection, to the first description of total mesorectal excision by Bill Heald and colleagues in 1982. This gave rise to clinical trials in Scandinavian countries that were to change global clinical practice.

The recent breakthrough in controlling cervical cancer through the use of a vaccine directed against certain types of human papilloma virus (HPV) rests on the work of Harald zur Hausen, who was first to show that the papilloma virus was the most significant cause of this cancer. In turn, that work owed much to the groundbreaking research begun in 1910 by Peyton Rous who first discovered tumour viruses.

Research into the molecular and cellular biology of cancer has provided remarkable insights into the molecular basis of cancer, such as disordered cell proliferation, disturbed differentiation and altered cell survival, and disruption of normal tissue, invasion and metastasis. New discoveries in the molecular oncology of tumours in the last few decades have led to major improvements in cancer therapy. In the middle of the 20th century an improved molecular classification of malignant lymphomas paved the way for individualised therapy in cancer. The treatment based on these molecular classifications resulted in higher response rates and improved survival of patients with malignant lymphomas. One of the most prominent scientists involved in this molecular pathology research and one of the authors of the new Kiel classification of lymphomas was Karl Lennert, a German Pathologist.

The field of breast cancer, the most frequent cancer in women, has seen many new developments based on the European research shared with other countries. Pivotal experiments performed in the late 1950s and early 1960s, primarily in the laboratories of Gerald Mueller and Elwood Jensen in Germany and the USA, set the stage for the development of hormonal therapy in hormone-responsive breast cancer. Acknowledgement of hormone receptors as one of the major biological determinants of breast cancer was actually one of the first discoveries that enabled the most effective strategies in the treatment of cancer, which is targeted therapy. Hormonal therapy with tamoxifen was the first individualised, targeted therapy in the history of cancer therapy.

Nowadays, breast cancer can be divided into hormone-receptor-positive and hormonereceptor-negative tumours, with treatment being substantially different in these two distinct diseases. Based on the largest meta-analysis in cancer care, undertaken at Oxford by Sir Richard Peto and his co-workers in the Early Breast Cancer Trialists’ Collaborative Group, a vast amount of knowledge on the best possible adjuvant systemic therapy in hormonepositive and hormone-negative breast cancer was accumulated. Their work confirmed that adjuvant chemotherapy reduced the rate of recurrence by 33% and the rate of breast cancer death by 17%, saving thousands of lives of women with breast cancer. The same was true for hormonal therapy in hormone-responsive breast cancer, in which adjuvant hormonal therapy with tamoxifen was found to reduce the rate of recurrence by 41% and the rate of breast cancer death by 34%, according to the data from the meta-analysis. Adjuvant systemic therapy, in addition to surgery, radiotherapy and screening programmes has been responsible for major declines in breast cancer mortality during the last two decades in the USA and Europe.

In 1971 when Richard Nixon proclaimed war on cancer ‘a quick and decisive victory was predicted’.^f^ However, despite the expenditure of billions of pounds and some real improvements in survival, especially in paediatric oncology, the morbidity and mortality associated with cancer remains high. Although the majority of cancer treatments are due to the surgical scalpel and radiotherapy it is advances in chemotherapy that hold the key to controlling and ultimately defeating cancer [1].

## 2.2. New Paradigms in Chemotherapy

The problem with conventional cytotoxic chemotherapy is rather obvious – the therapeutic window is narrow and only partially effective. This leads to poor efficacy and tolerability, as well as the development of serious adverse reactions. Combination chemotherapy has been the intellectual development to address the former issue but at the expense of tolerability and toxicity. Chemotherapy that targets all abnormal cells whilst sparing normal tissues has seemed a distant prospect, but now the ability to probe the most intimate details of the cell through the application of genomic and proteomic technologies has the potential to fulfil this dream. At the heart of this is the vision that new chemotherapy can be developed to selectively target cancer [2]. The new paradigm couples technology, such as robotic high-throughput screening, combinatorial chemistry, structural biology and molecular modelling with new insights into the pathophysiology of cancer and therefore new therapeutic strategies, for example the role of new blood vessel formation for metastatic disease and development of anti-vascular and angiogenic agents [3]. There have been encouraging signs that this approach may work. A signal transduction inhibitor (Imatinib) that selectively targets the abnormal BCR-abl fusion protein (Philadelphia chromosome product) that drives Chronic Myeloid Leukaemia, has demonstrated remarkable efficacy, with little toxicity. The more that we learn about cancer biology the more approaches present themselves – growth factor receptors, immune system modulation, cellular matrix, targeting of proliferation, migration and survival (apoptosis).

Natural product drugs that continue to play the dominant role in armoury of therapeutic options are also complimenting designer chemotherapy. Some of today’s most clinically successful chemotherapies are derived from nature – paclitaxel, vincristine, vinorelbine and analogs of camptothecin [4]. In fact, in the last 50 years only the rational structural design of 5-fluorouracil by Heidelberger has bucked the serendipity trend [5]. However, since the early 1990s this has changed dramatically with agents designed against rational targets – cetuximab, bevacizumab, to name but two. The hope is that efficacy can be enhanced by combining these cytotoxics with the newer targeted agents. Furthermore, it has become apparent that individual responses – efficacy and toxicity – are in part determined by genetically determined factors. This has led to the emerging disciplines of pharmacogenomics and pharmacogenetics that, using emerging technologies such as single-nucleotide polymorphism typing, aim to tailor chemotherapy [6]. However, this is still a very nascent field that has yet to be fully validated.

The past 10 years has seen a paradigm shift in cancer drug development away from direct acting anti-cancer agents, be they antibodies, novel signal transduction inhibitors or conventional cytotoxics, which have been at the heart of chemotherapeutic strategies for more than 40 years. Conventional wisdom underpinned by solid evidence from randomised controlled trials dictates that direct tumour cytotoxicity is an effective strategy. However, in spite of success in curing a variety of cancers from paediatric to adult with modest gains in the adjuvant/neoadjuvant setting for a subset of patients as well as much needed palliation in other settings, the fact remains that new strategies, or combinations of strategies, are needed to deal with advanced, metastatic disease.

## 2.3. Attacking Cancer

Cancer cells, despite their escape from normal intra- and extra-cellular controls, are still highly dependent on interactions between their cell surface receptors and other cells (cell-to-cell adhesion), growth factors, cytokines, hormones and elements of the extracellular matrix. Furthermore, they must continue to evade immune detection and, beyond a certain size, need to stimulate new blood vessel growth – angiogenesis. Targeting the world around the cancer cell is role of indirect acting anti-cancer agents.

Remarkably, targeting the tumour blood vessels as a therapeutic option was first suggested some 20 years ago by Juliana Denekamp and colleagues at the Gray Laboratory writing in the *British Journal of Cancer*. Aided by quantum leaps in technological development, particularly in real-time imaging of vascular flow and function through such techniques as dynamic contrast-enhanced – magnetic resonance imaging (DCE-MRI), positron emission tomography (PET) and laser doppler flowmetry, targeting angiogenesis has been a leading research area. Two broad fronts have been engaged. One approach targets the intracellular protein tubulin and has the dual attraction of acting as a mitotic poison for tumour cells and an antivascular agent. Various novel candidates are in development, and early phase clinical development has been completed for combretastatin A4 phosphate [7].

The other front has been to exploit the differential expression of endothelial surface proteins. Various approaches are being trailed, including immunotoxins and targeted gene therapy. Multiple mode-of-action agents^g^ are also being examined. One of the most promising agents to demonstrate exceptional antivascular properties is a low-molecular weight compound termed DMXAA, derived from the non-steriodal anti-inflammatory flavone acetic acid [8]. This is currently the focus of a number of studies. More advanced and well-validated approaches have been through the targeting of blood vessel growth factor receptors utilising recombinant humanised monoclonal antibodies or small molecule inhibitors.^h^

The dual finding that many solid cancers not only have deregulated signalling pathways but also trigger new blood vessel formation by the cancers suggests a need for combination direct/indirect strategy. One such new molecular entity that takes this approach is a pyrrolo-pyrimidine derivative (AEE788), and dual inhibitor of both these pathways, which has reported activity in pre-clinical models, particularly head and neck squamous cell carcinoma, a tumour notoriously resistant to treatment.^i^ Although exciting science, it remains to be seen whether these ‘2-in-1’ agents are better than combining two different classes of agents, for example antibody against VEGF and tyrosine kinase inhibitor for EGFR or vice-versa. Interestingly, it is not just novel compounds with dual mechanisms-of-action (indirect & direct acting) that are attracting interest. Thalidomide appears to exert its anti-cancer activity through numerous actions via growth factors tumour blood vessels and cytokine modulation.

Broadly speaking, immuno modulation^j^ has not proved itself yet to be the success that William Coley’s seminal work in the 19th century suggested it could be. Coley, a New York Surgeon demonstrated clinical remission in advanced cancer patients treated with an mixture of inactivated ***S. pyogens*** and ***Serratia marcesens***, work that was not reported until after his death by the careful scholarship of his daughter Helen Nauts [9]. Modulation of innate and adaptive immunity has been a complex and difficult task. One approach has been to target cytokines^k^. A number of these molecules with a variety of immuno-modulating properties are currently being assessed in clinical trials with some (e.g. IL-2, IFN-α/γ, TNF-α) at late stage development [10]. Many cytokines have also been shown to be important survival factors for various tumours, for example IL-6 for breast and prostate cancers. IL-6 has also been shown to enhance pancreatic cancer survival, cell migration and proliferation in the presence of HER2 inhibition. Such insights are vital for guiding potential combination therapies, in this case for instance this work suggests that without IL-6 inhibition any HER2 targeted therapy, for example trastuzumab (Herceptin) would be at the very best ineffective. A dual indirect/direct acting strategy is also being employed by the generation of antibodies fused to cytokines [11]. Recombinant cytokines are also being combined with other indirect acting immunological therapies including cancer vaccines [12].

Numerous approaches have been utilised in developing cancer vaccines from tumour antigens to naked DNA or RNA [13]. Despite early disappointment that vaccines failed to demonstrate clinical efficacy, promising immunostimulation has been achieved, and new approaches such as DNA vaccines, particularly if used in combination therapies, have the most realistic prospect of success. Likewise, promising early results with dendritic cell vaccines (DC’s are essential for the induction of adaptive immunity) have not been replicated in larger trials. Part of the problem may lie in DCs’ interactions with growth factors [14]. Either such inhibitory networks need to be circumvented or inhibited by targeting the relevant growth factor.

Anti-cancer agents targeting the tumour environment are essential for a number of reasons, namely the ability of cell-cell and of cell-matrix interactions to:

- enable immune evasion;
- act as proliferation and survival signals;
- enhance migration (metastasis);
- enable tumours to acquire multi-drug resistance (MDR).

Part of the problem with targeting the tumour environment has been the difficulty in identifying key, rate-limiting factors, and therefore potential targets that support cancer cell proliferation and survival. Even when such factors are identified, there remains the difficulty of tumour selectivity in any therapy designed against a component of the normal extracellular matrix – the tissue architecture in which normal cells function. However, there are encouraging signs of progress in identifying novel targets in the extracellular matrix that surrounds cancer cells and in further determination of their pathophysiological role. Spangaletti and colleagues have recently demonstrated the importance of a basement membrane organising protein, ***Sparc***, secreted from stromal cells, for the immune protection and development of new blood vessel formation for breast cancer cells [15].

The observation that the extracellular matrix surrounding tumour cells can protect against inhibition with new chemical entities targeting signalling pathways suggests, again, a need for dual direct/indirect anti-cancer therapies. The multi-factorial nature of drug resistance will also require combination therapies that can target a variety of environmental factors from extracellular matrix proteins to key growth factors and cytokines [16].

Development of novel screening methodologies, such as target-related affinity profiling (TRAP), have also led to further developments towards first-in-man trials, in this case the identification of novel small molecule lead compounds against targets implicated in promoting tumour proliferation and survival.^l^ Another major area of research has been in preventing cancer cell migration/invasion through the use of matrix metalloproteinase inhibitors such as marimastat, and the Bayer and Bristol-Mayers-Squibb compounds BAY12- 9566 and BMS275291. The universally negative results to these compounds to date indicate a deficiency in our knowledge about the actual pathophysiological of migration/invasion. More research is needed to understand the complex interplay between tissue architecture, secreted growth factors, non-cancerous and cancerous cells if rationale combination strategies are to be formulated.

Will these strategies be a solution? By themselves it is unlikely, but in combination (e.g. targeting various in-direct domains – angiogenesis, growth factors, etc. or with direct acting agents) there is real potential. Substantial difficulties remain – resistance secondary to plasticity in the various signalling pathways, the potential for significant side-effects, insufficiently ‘potent’ anti-cancer activity and the difficulty of designing agents with sufficient selectivity, as well as the perennial problem of delivery.

In his concluding remarks to the 12th Annual Pezcoller Symposium, Ed Harlow commented that it would be important to utilise complex read outs to spread information on how different actions interact in a cell as well as the combined expertise of different laboratories; this was just to address single-cancer cell signalling pathways [17]. In fact, integrating anti-cancer strategies is an order of magnitude greater in complexity for dissecting biological crosstalk and key interactions compared to the level of single cancer cell and its signalling pathways. There remains much to understand mechanistically about tumour-environment interactions, but the goal must be to try out these combinations in imaginative and perhaps even in empirical manners. There is now good evidence that tumour responses are a group phenomenon rather than the summed responses of individual cells to injury. Thus the classic description of the bystander effect in radiation oncology may be relevant to chemotherapeutic strategies where indirect and direct acting anti-cancer agents are combined to substantially enlarge the direct injury to the tumour cells.

How is this likely to be best achieved? Clearly, the parallel investigation of cancer biology and selection of new indirect/direct-acting combination strategies requires integrated clinical studies that maximise pharmacological, cellular biological and molecular pathological information capture. The ability to take time and explore unusual or counter-intuitive avenues will be essential. Strong academic centres supported by high-quality organisational structures funding specific projects underpinned by long-term programmatic funding are the ideal environment to investigate these complex areas [18]. This strength has already led to substantial biotech spin-offs and provides a rich environment within which public-private partnerships can work to tackle these therapeutic challenges.

## 2.4. Biologicals for Cancer Therapy

The last decade has seen a surge of research and development focused on biologicals, which must be considered now as being one of the hottest areas for anti-cancer drug discovery.

Last year was the celebration of the 100th anniversary of the ‘birth’ of immunology, following the Nobel Prize Award to Paul Ehrlich and Elie Metchnikoff. Ehrlich was the first to demonstrate humoral adaptive immunity (the antibody arm of the immune system) whilst Metchnikoff’s discoveries of the critical host-defence of phagocytosis, notably the engulfment of cellular debris and pathogens, made him the father of cellular innate immunity. As in all matters of science, progress since these heady days have not been smooth. A chemical explanation for Ehrlich’s discoveries was sought, but quickly came to a dead end. It took Frank Burnett’s and David Talmage’s theory of clonal selection to bring matters back on track, but it would not be until 1939 that Susumu Tonegawa would solve antibody diversity, using the then newly acquired tools of molecular biology, and so set in motion developments, which were to lead to today’s great array of therapeutic antibodies. On the other hand, it took nearly a 100 years to fully understand what Metchnikoff started that non-specific cellular and specific humoural system are complimentary and of equal importance [19].

Immunotherapy to treat cancer also had the good fortune to find one William Coley, then at the hospital destined to become the Memorial Sloan-Kettering, who first used bacterial toxins to induce responses in sarcoma [20]. Whilst extraordinary progress has been made in the development of cancer biologics as well as their application in other diseases (e.g. in monoclonal antibodies – both therapeutic and diagnostic; therapeutic interferon’s and interleukins and human growth factors for supportive care), disappointments and set-backs still fog this therapeutic domain. Despite, or perhaps because of these issues, immunotherapy generates huge excitement.

The recent TGN1412 (anti-CD28 monoclonal) clinical trial for certain types of blood cancers gave an unwelcome insight into how much and yet how little we really understand about the human immune system. With so much work carried out on animal models it is now becoming clear that the only realistic model is man. As Adrian Hayday and colleagues point out we do not even have a good physiological definition of a healthy human immune system [21]. From an evolutionary standpoint, it is perhaps no surprise that as a species we seem to be so very immunologically different. The hominin lineage (the evolutionary line from which Homo Sapiens descended) has penetrated every ecological land niche on this planet and immunological plasticity has been mandatory. Indeed, from the 30 or so extant animal phyla examined by comparative immunology, it is now clear that there has been a huge acquisition of immuno-complexity, leading to totally unforeseeable alternatives in immune receptor diversification and systems interaction. In light of this knowledge, developing complex immunotherapeutics is inevitably an exercise in stochastic experimentation rather than rationale development.

In an excellent article, Antony Melcher, Peter Selby and colleagues set out the state of knowledge in how the human immune system does (or not) respond to cancer [22]. Whilst studies of tumour-infiltrating lymphocytes (TILs) provide a strong case for immunosurveillance of established cancer, precious little is known about the ability of the immune system to detect and clear pre-clinical tumours. Animal model(s) have suggested a role for NK, NK T cells, alpha/beta T cells as well as gamma/delta T cells. New concepts – such as immunoediting composed of three phases of elimination, equilibrium, escape – if supported by further *in vivo* studies would greatly aid many avenues of immunotherapy development.

In the same vein, work over the last 5 years has placed the relationship between the emerging tumour and stromal tissue centre stage. This complex and dynamic process injects a new paradigm into the overall theory of immunoediting by seeing immune-cancer interactions across the spectrum from quiescence to low-level inflammation and to full blown acute reactions. Specific studies on how tumour cells interact and exploit complement open up new theoretical avenues for therapy.

Part of the translation problem lies in the fact that some of the ‘basic’ knowledge about the human immune system is lacking. The question that has exercised researchers is how to address this. whilst ‘big science’ is always controversial, the jury appears to be coalescing around the idea that a human immunological genome project is needed to establish essential genetic road maps [23]. The obvious problem is that this would still be a long way off from understanding the 4-D immuno-oncology system (the interaction between the immune system and the initiation and development of cancer), but in order for the systems biologists to really understand such a problem perhaps the time has arrived to launch such an initiative. Such a project, properly integrated with some of the key global programmes in cancer immunotherapy R&D, might well fill in key lacunae in our basic understanding.

Broadly, the efforts to treat cancer with immunotherapy have included the use of cytokines (e.g. IL-2 to treat malignant melanoma), adjuvants^m^ (e.g. BCG to reduce recurrence of bladder cancer), the far less successful attempts utilising vaccines and other approaches. The crowning achievement has been the use of humanised monoclonal antibodies (MAb) that continue to be developed into new therapeutic niches, for example by the harnessing idiotypic networks to elicit tumour-antigen specific immune responses. Moreover, MAb make commercial sense with studies, showing a higher success rate than small molecules (14% compared to 10% between 1990–2007). In an attempt to leverage the success of trastuzumab and bevacizumab pharma continues to re-stock its immunotherapy pipeline with biotech specialising in humanised antibodies (MAb). Unfortunately, however, many cancers develop resistance to MAb, or are refractory from the outset, which has spurred a raft of work into immunotoxins. These immunoconjugates, notably processes where an antibody is bound to a novel ‘warhead’, are now being developed to use a wide range of ‘warheads’, including chemotherapeutic agents, radioisotopes, enzymes or toxins [24]. Some immunotoxins, for example IL-13 and EGFR targeted agents, are now in Phase III clinical trials (both against glioblastomas^n^), but the majority remain at an early stage of development.

One of the major issues is, whereas haematological malignancies appear to be responsive, solid tumours are not, probably due to a combination of immune system impairment secondary to previous therapy and the lack of accessibility of the immunotoxin. One fascinating approach to tackle solid tumours is by targeting cancer stem cells (CSC) with MAb. This exciting area suggests that the major cause of conventional treatment failure is an inability to kill off the CSC, which then give rise to a highly aggressive and resistant population [25]. Three approaches are now in pre-clinical development – OMP-21M8 against multiple solid tumours, RAV17/RAV18 against prostate and colon and ARH460-16-2 against leukaemia, breast, colon and prostate. All these biologicals utilise novel and complex mechanisms of action to target cancer stem cells.

Beyond the enhancement of MAb immunotherapy using toxin conjugates, one of the key areas for development is their combination with chemoradiotherapy. In the case of radiotherapy, the evidence that it can promote sufficient ‘danger’ signals to enhance immunotherapy, particularly, lymphocyte trafficking, is thin. However, there is 20 years old data, which suggest that such an effect may be possible [26]. The need now is to follow this up with specific studies. The ability of chemotherapy to augment immunotherapy is highly attractive. There are numerous ways in which this could, from a mechanistic standpoint, be achieved but the myelosuppressive (reduction in bone marrow activity) nature of many regimens coupled with many tumour kill mechanisms often generating either weak danger signals for immunotherapy and/or tolerating effects is a serious challenge. Two notable recent failures, both in lung cancer, provide salutary lessons. The first was the failure of a MAb bevacizumab & EGFR inhibitor erlotinib combination to arrest non-small cell lung cancer (NSCLC), and second, the poor showing of a heat killed suspension of mycobacterium vaccae (SRL172) again in a Phase III against NSCLC. There were tantalising indications of activity in both cases, particularly an enhancement of response rate and median progression free survival in the first combination. However, the effect may well have been diluted out by NSLC heterogeneity as well as an enhancement of survival from additional therapies after each progression.

Two clear lessons come out of these experiences. The first is the need to be much more selective about patient selection criteria and tumour type, although this does then beg the question as to the commercial viability if indications become increasingly narrow, and the second is the need to rebuild and maintain the immune system if immunotherapy is to be an effective add-on to conventional chemotherapy [27]. Furthermore, defining immunogenic regimens and schedules will be key to taking this approach forward; for example temozolomide is known to be a powerful inhibitor of the immune system and gemcitabine depletes B-cells and so would be a poor choice for combination therapy with a biological.

There are four important issues to consider in designing an effective cancer vaccine: how to prevent immune evasion, how to avoid autoimmune pathology, how to stimulate an effective anti-tumour response, and finally, how to identify potent tumour rejection antigens [28]. Sadly, few have made it through to the clinic and when one takes the most cursory look at the range of approaches it is clear that this field is full of complexity. One of the main approaches has been to engage cell-mediated immunity through either using isolated antigen presenting cells or attempting to stimulate them ***in vivo***. Thwarted by the relative recent finding of active immunosuppression within the tumour microenvironment, a whole range of new strategies have emerged to circumvent this. Such complexity and inherent development risk has meant that fewer than one fifth of oncology biologic therapeutics in pipeline are vaccines. The upside is the creativity of approaches in both early and late stage development. The majority of late stage clinical trial promise is focused on either whole-cell-based autologous or allogeneic tumour cells (off the shelf) approaches, but the great range, outcome uncertainty and increasing costs make this a high-risk area. Delivery has also been a key technical challenge. As Freda Stevenson and colleagues discuss in the context of DNA vaccines, this has been a challenging area, but novel approaches such as electroporation, namely using electric charge to ‘force’ the uptake of DNA, may solve the delivery issue [29]. In summary, cancer vaccine development has had a many false starts but creative approaches and new technologies certainly increase the chance that a winner(s) will be found in the next 5 years.

Our next exploration perhaps gives some idea of the vast range and complexity of cancer immunotherapy. It is arguably one of the most challenging approaches to cancer immunotherapy and yet is utterly absorbing science. Tumour-targeted oncolytic viruses (virotherapy with replication-selective viruses) have a range of important features that make them a viable immunotherapeutic option, in particular the potential for selective replication in tumours to increase the therapeutic index, the lack of cross-resistance with conventional chemoradiation (viruses kill by a numerous other mechanisms) and the ability to circumvent either tumour or iatrogenic-induced immunosuppression [30]. The technical challenges to achieve tumour-selective virus replication are focused around four areas – limiting the uptake of viruses into tumour cells whilst ablating the uptake into normal cell populations, deletion of gene functions that are critical for replication in normal cells, limiting expression of the E1A gene product to tumour tissues, or using a virus, for example reovirus that is inherently tumour selective. A whole slew of approaches using adenoviruses, herpesvirus, vaccinia virus, reovirus, etc. are in pre-clinical development. Importantly, for such a diverse and complex area, a well-characterised and quantitated virus ***dl***1520 ONYX- 015 has provided important proof-of-concept data in an early clinical trial setting. Whilst much has been achieved in this area, for example demonstrating the feasibility of virus delivery through the blood stream to tumours, major barriers such as the lack of model systems and potency issues remain.

Finally, there are a whole range of novel approaches to immunotherapy, which are not easily categorised. Immunostimulation as a way of directly killing tumour cells and/or indirectly improving chemoradiation effects is being explored. Rationally designed approaches utilising MAb have been proposed despite the recent toxicities seen with the use of the ‘super-agonist’ anti-CD28 MAb (TGN1412) serving as a sobering lesson in the need for careful and cautious research and development. However, for cancer, drug development biologicals remain a rich and increasingly diverse source for new approaches.

## 2.5. Concluding Remarks

It has been barely 50 years since the cancer clinician had but a handful of chemotherapeutic agents with which to tackle the huge range and diversity of cancers. There has been an extraordinary explosion in both our understanding of cancer as a disease and in the evolution of new molecular entities. Research and development of anti-cancer agents has become a highly complex, globalised endeavour, and whereas once the private and public sector ploughed separate paths the needs of cancer science and the proliferation of the anti-cancer pipeline have slowly but inextricably pulled these ‘two cultures’ together.

This Chapter has provided some sense of the complex science that underpins cancer drug development. Clearly, this will increase with the molecular stratification of cancers, and novel prognostic/predictive biomarkers. Policies that understand and evolve complex systemic approaches to research organisation and management will be essential to deal with the broad church of cancer drug development. The classical linear pathways of R&D are redundant and a new paradigm is required, one that harnesses the strengths and opportunities of both private and public sector.

## References

[1] Cufer T, Sullivan R, Coleman MP, Alexe D-M, Albrecht T, McKee M (2008). Researching cancer. Responding to the challenge of cancer in Europe.

[2] Gelmon KA (1999). Anticancer agents targeting signalling molecules and cancer cell environment: challenges for drug development?. J NCI.

[3] Hayes AJ, Li LY, Lippman ME (1999). Antivascular therapy: a new approach to cancer treatment. BMJ.

[4] Pezzuto JM (1997). Plant-derived anticancer agents. Biochem Pharm.

[5] Heidelberger C (1957). Fluorinated pyrimidines, a new class of tumor inhibitory compounds. Nature.

[6] Sadee W (1999). Pharmacogenomics. BMJ.

[7] Stratford M (2000). Phase I pharmacokinetic and toxicity study of weekly intravenous combretastatin A4 phosphate. Clin. Cancer Res.

[8] Baguley BC (2003). Antivascular therapy of cancer: DMXAA. Lancet Oncol.

[9] Nauts H, Fowler G, Bogatko F (1953). A review of the influence of bacterial infection and of bacterial products (Coley’s toxins) on malignant tumours in man: a critical analysis of 30 inoperable cases treated by Coley’s mixed toxins, in which diagnosis was confirmed by microscopic examination selected for special study. Acta Med Scand.

[10] Dranoff G (2004). Cytokines in cancer pathogenesis and cancer therapy. Nature Reviews Cancer.

[11] Davis CB, Gillies SD (2003). Immunocytokines: amplification of anti-cancer immunity. Cancer Immunol. Immunother.

[12] Indar AA, Maxwell-Armstrong CA (2003). Active specific immunotherapy for colorectal cancer. Expert Rev Anticancer Ther.

[13] Lysaght J, Todryk S (2003). Developments in cancer vaccination. Curr Opin Investig Drugs.

[14] Kobie JJ (2003). TGFb inhibits the antigen-presenting functions and antitumor activity of dendritic cell vaccines. Cancer Res.

[15] Sangaletti S (2003). Leukocytes rather than tumour-produced SPARC determines stroma and collagen type IV deposition in mammary carcinoma. J Exp Med.

[16] Shain KH, Dalton WS (2001). Cell adhesion is a key determinant in de novo MDR: new targets for the prevention of acquired MDR. Mol Cancer Ther.

[17] Mihich E, Harlow E (2000). Signalling cross-talk in cancer cells. Cancer Res.

[18] Newell DR (2003). Professor Tom Connors and the development of novel cancer therapies by the phase I/II clinical trials committee of Cancer Research UK. BJC.

[19] Kaufmann SH (2008). Immunology’s foundation: the 100-year anniversary of the Nobel Prize to Paul Ehrlich and Elie Metchnikoff. Nat Immunol.

[20] Coley WB (1891). II Contribution to the Knowledge of Sarcoma. Ann Surg.

[21] Hayday (2008). Assessing the status of human immunology. Nat Immunol.

[22] Prestwich RJ, Errington F, Hatfield P, Merrick AE, Ilett EJ, Selby PJ, Melcher AA (2008). The immune system–is it relevant to cancer development, progression and treatment?. Clin Oncol (R Coll Radiol).

[23] Heng TS, Painter MW (2008). The Immunological Genome Project: networks of gene expression in immune cells. Nat Immunol.

[24] Pastan I, Hassan R, Fitzgerald DJ, Kreitman RJ (2006). Immunotoxin therapy of cancer. Nat Rev Cancer.

[25] Dick JE (2009). Looking ahead in cancer stem cell research. Nat Biotechnol.

[26] Rosenberg SA, Spiess P, Lafreniere R (1986). A new approach to the adoptive immunotherapy of cancer with tumor-infiltrating lymphocytes. Science.

[27] Vuk-Pavlovic S (2008). Rebuilding immunity in cancer patients. Blood Cells Mol Dis.

[28] Gilboa E (2004). The promise of cancer vaccines. Nat Rev Cancer.

[29] Rice J, Ottensmeier CH, Stevenson FK (2008). DNA vaccines: precision tools for activating effective immunity against cancer. Nat Rev Cancer.

[30] Hawkins L, Lemoine N, Kim D (2002). Oncolytic biotherapy: a novel therapeutic platform. Lancet Oncol.

# Sub article: 3. Public and Private Funding for Cancer Drug Research and Development

**Published:** 2010-02-03

## 3.1. Background and Objectives

In policy terms, critical issues around sustainability, productivity and patient impact of cancer drug development have never been and will not simply be a product of industry. With such an extensive portfolio of present and future cancer medicines, the funding contribution of the public sector is absolutely vital. However, public policy decisions around investment in cancer research need to be anchored in evidence, and one of the necessary key understandings is the source and flow of capital and revenue to support the themes or domains of research.

A significant number of publications to date on the funding of cancer drug development and indeed other disease-specific domains have focused on the funding by industry [1,2]. Although there is controversy around the use of single numbers [3], there is currently a much clearer picture of the expenditure by industry on the full development costs of cancer drug development for policy making. Yet, annualised figures for contribution from the private sector to cancer drug development specifically are also missing.

A major issue lies in the fact that there are almost no reliable estimates of what the public sector (philanthropic and federal / governmental spending) contributes to cancer drug development. Broad-brush policy research has addressed some aspects of public sector investment, for example the US Institutive of Medicine (IOM) review of public sector cancer funding from the late 1990s [4], the mapping of drug development expenditure for public sector at national level in the United Kingdom [5] and an updated map of European / USA public sector spend on general cancer research [6], which provide useful denominators. However, data on how these funds do (or do not) interconnect with private sector (industry) funding or, indeed, from whom this funding flows and how, are not available (Figure 3.1).

The public sector is both source and sink for a huge range of research activities in cancer drug development. Furthermore, it is the public sector, which mostly trains and hosts today’s and tomorrow’s drug development research faculty, both clinical and non-clinical. Broadly speaking, there are two major sources of public sector funding for cancer drug development, first, philanthropic (which can again be sub-divided into endowed charities, e.g. the Wellcome Trust, and annual fundraisers, such as Cancer Research UK) and, second, federal (through either federal funding organisations or ministries, e.g. BMBF in Germany, or as general infrastructure funding to host institutions – hospitals/universities sector). In the latter case, increasingly, major cancer centres and other host institutions are generating their own sources of revenue and capital for cancer research.

Understanding public and private spending on cancer drug development either to support direct research costs or through general infrastructural funding is essential so as to build a coherent picture of the long-term health and stability of cancer research and to understand the future of cancer drug development.

The objectives of this chapter are to:

1. understand which major public sector research funding organisations are supporting cancer drug development and how this is aggregated by country and region;
2. compare public sector investment by country and region, compare and highlight which region(s)/countries have committed public sector support for cancer drug development and which have not. How does this heterogeneity translate to national and regional strategies in this area, for example is the European Innovative Medicines Initiative building on strong foundations?
3. explore whether and how cancer research funding flows directly to projects or indirectly through general infrastructure funds;
4. quantify the relative contributions of individual countries and understand how this compares with their overall cancer research spends;
5. understand the breakdown of public sector funding by the highest common scientific outline level, for example cancer treatment development relative to spending in other areas, for example fundamental cancer biology;
6. outline the relative contributions of charities and NGOs versus governments;
7. distinguish between the contributions to cancer R&D by political grouping in Europe, that is EU member states, accession countries and European Commission;
8. estimate the annual direct spend by major pharmaceutical companies, stripping away associated costs up- and downstream, in order to provide a more complete picture of available funding sources;
9. explore the public-private interface (Figure 3.1) and how funding flows to cancer drug development along this critical boundary;
10. discuss how the sources and sinks of funding compare between countries, and whether there are policy learning points from those deemed ‘successful’;
11. determine what trends emerge in terms of overall ‘financial commitment’ to cancer drug development research, by using high-resolution studies of cancer centres, and the research portfolios of the United Kingdom, USA and Canada.

## 3.2. Methods

In seeking to extract data from both public sector and industry organisations as to their funding of cancer research and cancer drug development, a survey of public and private sector spending on cancer drug research and development was conducted with a view to obtaining hard data on monies expended on research. Furthermore, in order to provide background information as well as to compare the present results to those of previous studies, a literature review was also carried out.

### 3.2.1. Surveying public sector spending on cancer drug research and development

The inclusion criteria for this part of the survey related to (a) the choice of country, (b) defining and contacting individual Research Funding Organisations (RFOs) for cancer (c) classifying the categories of spending for the identified organisations into recognisable groups to enable comparisons and (d) distinguishing between direct and indirect cancer funding.

With regards to country selection, all EU27 Member States together with European Free Trade Association (EFTA) countries, Israel and Turkey were included as part of the ‘Europe’ region to reflect the breadth of public sector collaborations between countries. Such a broad number would also allow a sub-analysis of different R&D systems. The USA was also included in the analysis.

With regards to RFO for cancer, they were defined as those public sector bodies with spending over one million Euros per annum, as below that level there were estimated to be more than 1500 smaller charities in EU15 alone. These organisations were classified according to whether they were a ‘federal’ (governmental) funder or philanthropic. Federal funders could be either ministries or arms-length government funding bodies, whereas philanthropic organisations would be either annual fundraisers or endowed charities. The identification of the organisations was based on broad studies of the public funders of cancer research across Europe [7], the USA [4] and, more recently, Canada.

Definitions for categories of spending, for example cancer control, biology, treatment were derived from the International Cancer Research Portfolio (ICRP) definitions. The ICRP enables direct access to cancer research information. Cancer research funders from several countries have joined in a partnership to classify their research portfolios in a common manner. Using the Common Scientific Outline (CSO) as a unified classification system, the ICRP allows users to search and view cancer research in a variety of ways, including by type of cancer, by area of research and by funding organisation.

A distinction was also made between direct and indirect cancer research funding. Direct cancer research funding was defined as funding originating from RFO to specific host institutions in the form of grants. It does not include educational grants, non-research staff salaries, physical plant improvements, spending on advocacy and service delivery. Indirect cancer research funding is funding derived from general taxation allocated to support host institution infrastructure and is usually given as a block general grant by government authorities.

A standard procedure was followed for surveying the RFO for self-reported spending on cancer drug development involving five distinct steps, as follows:

First, letters were sent to the Directors/CEO’s of each RFO requesting funding data for 2007/08. The letters provided a full explanation of the project. As most RFOs are obliged to provide public figures for spending and had been cultivated, there were no outright rejections.

Second, RFO websites were interrogated to ascertain whether self-reported figures matched published figures.

Third, when received, the requested financial information was reviewed and crosschecked. Guidelines were established to help with this quantification and were followed through the entire data collection and data entry phases. For instance, if a funding organisation reported spending levels between two amounts, the higher amount was always used. Any RFO reporting spend in currencies other than Euro had the reported amount of spend converted using the web site www.xe.com, all currencies were converted within two days of receipt of the information.

Fourth, the United States spending on cancer drug development was based on RFOs identified previously [8]. Many RFOs in the USA, such as the Department of Defense and the National Cancer Institute, report their cancer research expenditure in annual reports and a breakdown of spend by specific intervention, that is cancer drug development. Furthermore, the RAND Corporation’s RaDiUS (Research and Development in the United States) database was also interrogated [9]. The database identifies by agency all intramural and extramural projects or tasks in which the search criteria appear in the title or abstract.

The following terms were used during the relevant search of the database: (a) cancer drug development and (b) new active substance, new molecular entity (including biologicals and new chemical entities as cross check MESH terms). These were believed to have the widest possible chance of collecting all relevant spending, without including projects out of the scope of interest of this survey. The abstracts of the projects were reviewed to extract projects that were not focused on cancer drug development (such as when cancer was only listed as criteria for exclusion in the study).

Fifth, certain cancer RFOs in the USA, United Kingdom and Canada belong to the ICRP, a very high resolution coding of spending against domains of cancer research. This database was interrogated for project spending levels/activity by the main funders of these countries.

### 3.2.2. Private sector contribution

Finally, in order to provide a more complete picture and given the fact that the private sector, that is pharmaceutical and biotechnology firms, are major investors on cancer research and particularly on cancer drugs, we also gave estimates for the annual direct spend by major pharmaceutical companies [7].

## 3.3. Findings

The challenging and complex task of developing new ground-breaking medicinal therapies for cancer relies on a collective and multifaceted effort of both public and private investment into cancer R&D.

Funding for cancer research comes from a complex network of private and public organisations and from a broad church of commercial enterprises to philanthropic causes.

Understanding ‘source to sink’ for cancer research funding is essential for the development of institutional and national policymaking. In cancer drug development, there are three broad domains for this funding:

1. The basic research that underpins the discovery of new targets and molecular entities against these targets, as well as the fundamental biological processes that drive cancer
2. The clinical development phase, including biomarker and associated translational research.
3. The postmarketing phase as new drugs are further developed in new regimens, in combination with radiotherapy or against new indications.

### 3.3.1. Public (non-commercial) funding

The majority of cancer R&D globally is carried out within the USA and the EU. Research can be funded publicly by the national governments or regional authorities (e.g. ‘Communidad Autonoma de Madrid), by various charities and NGOs, or by supranational organisations such as the European Commission within the EU. Funding deriving from cancer-specific organisations is considered as ***direct*** funding and can be either governmental, flowing through either federal funding organisations or ministries (e.g. BMBF in Germany, INSERM in France and the Netherlands Genomics Initiative), or philanthropic including charities such as the ‘Wellcome Trust’ (UK), the ‘Ligue Nationale contre le cancer’ (France) and annual fundraisers such as ‘Cancer Research UK’ and the Dutch Cancer Society. On the other hand, funding flowing through from general taxation usually as general infrastructure funding to host institutions – hospital and/or university sector is considered to be ***indirect***. In the latter case, increasingly, major cancer centres and other host institutions are generating their own sources of revenue and capital for cancer research.

In this section, the presentation of the leading cancer research organisations identified and included in the review will be followed by a series of metrics aiming to capture the performance of individual countries from different perspectives. The metrics used are (a) absolute direct public expenditure (investment) on cancer research and drug development, (b) proportion of total public investment on cancer research that goes into drug development, (c) a comparison between the absolute direct and indirect investment on cancer R&D in Europe and in the United States, (d) per capita direct expenditure (investment) in cancer research and development and (e) cancer R&D spend as a percentage of GDP.

#### (I) Research funding organisations

The inclusion criteria for this part of the survey with regards to:

1. ***Country selection*** included the USA and as part of the ‘Europe’ region all EU27 member states together with European Free Trade Association (EFTA) countries, Israel and Turkey to reflect the breadth of public sector collaborations between countries. It is noted that from now on – and unless stated otherwise – the term ‘Europe’ will refer to EU27 member states, the EFTA countries (i.e. Iceland, Norway and Switzerland), the EU candidate countries (i.e. Turkey) and the associate states (i.e. Israel). The EU Commission – and any funding deriving from it – will not be included in that term.
2. ***Research Funding Organisations*** (RFO) for cancer included all those public sector bodies with spending over one million Euros per annum and were classified according to whether they were a ‘federal’ (governmental) funder, such as ministries or arms-length government funding bodies, or philanthropic, such as annual fundraisers or endowed charities;
3. ***Definitions for categories of spending***, for example cancer control, biology, treatment were derived from the International Cancer Research Portfolio definitions classifying the categories of spending for the identified organisations into recognisable groups to enable comparisons and
4. ***Distinguishing between direct and indirect cancer funding***, included as direct cancer research funding the funding originating from RFO to specific host institutions in the form of grants. It does not include educational grants, non-research staff salaries, physical plant improvements, spending on advocacy and service delivery. As indirect cancer research funding was included the funding that derived from general taxation and is allocated to support host institution infrastructure and is usually given as a block general grant by government authorities.

In total, 153 non-commercial RFO that satisfied the above criteria were identified across European countries and 21 in the United States and were included in the analysis (Figure 3.2).

Apart from these 153 RFOs numerous, other smaller charities exist but were not included in the survey as their annual spending was less than one million Euros. For instance, it has been estimated that more than 1500 such charities are operating in EU15 alone. Their large number and their relative small direct contribution to cancer research made their inclusion inefficient, if not impossible. Although, this omission may lead to a slight underestimation of the overall level of public funding on cancer research, the goals of the analysis (i.e. to built a coherent picture of the long-term health and stability of cancer research and to understand the future of cancer drug development) are still achieved.

It must also be acknowledged that part of the federal funding is indirect, in the sense spending on hospitals/universities, and even though it is used for cancer research it is not explicitly earmarked. As a result, the survey mainly addresses direct cancer research investment and there may be underestimations on the level of indirect contributions.

Finally, funding flowing in from the European Commission is likely to be underrepresented, as the annual average during Framework Programme 6 was used, and it is expected that the Framework Programme 7 average will be higher.

#### (II) Summary of policies and organisations supporting cancer drug research and development in Europe and North America

##### The European Union

The EC has a number of programmes to help encourage cancer drug R&D. The primary overall research support programme is the Framework Programme (FP), which provides funds and support to all areas of research activity in Europe [10]. Cancer research is one of those activities, channeled into the ‘Combating Cancer’ initiative. Previous reports have criticised EU cancer research with its fragmentation and diversity, as well as different support mechanisms and funding bodies. Recent action has been proposed from the EC with ‘Action Against Cancer’, with financial support from the FP, as well as further support from the European Research Areas (ERA) whose objectives include forming partnerships between EU, national and regional research programmes, activities and policies [11]. As a result, the previous FP6 (2002–2006) invested some €480 million to 108 transnational cancer research projects, whilst the current FP7 (2007–2013) has to date invested €265 million to 65 projects and 700 research groups (one-third to large transnational projects), with more monies allocated in the future (Table 3.1).

Box 3.1: **Examples of Transnational Cancer Research Projects in the EU under FP7 [17]**

GENINCA: to examine cancer genome instability, in the hopes of leading to novel targeted cellular cancer treatments (11 partners in eight countries; €3 million 2008–2011).

ERA-NET: to increase cooperation and coordination of research, including cancer research, activities throughout the EU by networking national and regional level activities and mutual opening of national and regional programmes (€2 million 2009).

INFLA-CARE: to develop innovative anti-inflammatory strategies and novel agents for cancer prevention and treatment by studying inflammation-driven cancer (20 partners in nine countries; €12 million 2009–2014).

ADAMANT: to generate superior anti-cancer agents relying on antibody-based delivery of cytotoxics, radionuclides or immunostimulatory cytokines to vascular tumour antigens or tumour cell membranes (€3 million; eight partners in six countries).

The FP6 promoted research activities with long-term impact, which strengthened Europe’s general scientific and technological basis. In particular, funds were invested where European cooperation was seen to have significant benefit, striving for EU level research with many areas and levels of integration. This integration and broader longitudinal view were new compared to previous FPs. The FP7 continues along this same principle, with even greater interest in EU level cooperation and coordination of research activities.

Due to this transnational focus, many of the previous FP6 and current FP7 funded projects encompass many countries and research institutes (Box 3.1). Previous FP5 (1998–2002) research focused on molecular mechanisms underlying cancer, whilst FP6 and FP7 focus on translational research, to bring basic knowledge into practice more quickly (Boxes 3.1, 3.2). The FP6 funded EUROCAN+ PLUS project (€5 million budget) aimed to improve EU research coordination, bringing together the Karolinska Institute, Institute Gustave Roussy, Instituto Europeo di Oncologia, ministries and foundations. Further support for research coordination is brought by the ERA Networking project (ERA-Net), focusing on coordinating national and regional research organisations.

Box 3.2. **Examples of FP6 Transnational Cancer Research Projects in the EU, under Framework Programme 6 (FP6). aa, [12,13]**

EUROCAN+PLUS: to coordinate European national cancer research activities after initial consultation found significant fragmentation, poor leadership and poor sustainability as major barriers to innovative cancer research. Research duplication, wasting time and money, limited intellectual concentration, poor communication and tensions between funders and researchers were seen as additional obstacles. The project recommended to creation of an independent European Cancer Initiative (ECI) assuming responsibility for:

- stimulating innovative cancer research and facilitate processes;
- common voice for cancer research;
- interface between public and private cancer stakeholders;
- develop solutions to eliminate collaboration and coordination barriers;
- regulatory and legal issues.

This ECI should have a platform for translational research encompassing:

- coordination between basic, clinical and epidemiological approaches;
- formal cooperation agreements between cancer centres and research laboratories throughout EU;
- networking between EU funding bodies.

Creation of the ECI has yet to be developed (Aug 2009).

ATTACK: to improve engineered T-cell function and perform pre-clinical trials, with the potential application of gene therapy treatment (17 partners in eight countries; €11.9 million 2005–2010).

CANCER IMMUNOTHERAPY: to develop a therapeutic cancer vaccine with defined tumour antigens, refining vaccinations and combining vaccines with other cancer treatments (22 partners in eight countries; €12.2 million 2006–2010).

TRANSBIG: to develop individualised breast cancer treatment, to facilitate translational breast cancer research and to organise a clinical trial examining tumour genetic signature for use in targeted treatments (40 partners in 22 countries, €7 million 2004–2011).

Another major EC programme, which encourages cancer drug research and development, is the Innovative Medicines Initiative (IMI), a public-private partnership focused on speeding up the process of drug discovery and treatment [14]. This initiative is a collaboration between the EC and the European Federation of Pharmaceutical Industries and Associations (EFPIA), with the EC supporting research and development for Academic as well as Small to Medium sized enterprises, whilst Large Biopharma fund themselves, with equal support from each partner. The IMI has a new focus, the Joint Technology Initiative, to support public-private research cooperation for faster development of new medicines by addressing key bottlenecks in the drug development process (Table 3.1) [15,16]. The IMI is managed by an autonomous body in Brussels, with half the governing board composed of EC members and half by industry, with industry setting the first round of priorities.

Box 3.3: **Examples of National Cancer Research Projects Supported by NCI (USA) [22]**

- Molecular Targets Development Program identifies and evaluates potential molecular targets for drug development.
- Chemical Biology Consortium accelerates discovery and development of first-in-class targeted therapies, choosing high-risk targets of low interest to pharma industry
- Rapid Access to Intervention Development (1998) creates bridges between academic discovery and clinical trials, as well as supporting investigations into orphan diseases. To date, RAID has supported 133 projects, resulting in 21 small molecule and 25 biological investigation new drug approvals.
- Phase 0 clinical trial development, where low doses of investigational drugs are given to few patients in order to determine whether further investigation is worthwhile.
- Advanced Technology Partnerships Initiative (ATPI) new research facility to encourage collaborations between public, private and academic stakeholders.
- Office of Biorepositories and Biospecimen Research to ensure high-quality human specimens available for research and the creation of a non-profit national biobank.
- Action as broker between various public and private investors in clinical trials (Life Sciences Consortium) to ensure speedy contract negotiations (which can cost companies greater than $1 million in delays and take between 180–300 days). The LSC has created a Master Agreement template in 2008 (freely available online) to minimise contract delays due to language, now supported by the Department of Justice

A new initiative, European Action Against Rare Cancers, has recently been announced by the EC, as part of their ‘Action Against Cancer’ initiative [18]. This focus will be on rare cancers, both in prevention and treatment, with goals to be determined in the Fall of 2009. All but the major five cancers (breast, colorectal, prostate, lung and bladder cancers) are classified as rare disease [19].

Another organisation in Europe is the European Organisation for Research and Treatment in Cancer, whose aim is to stimulate translational and clinical research in Europe. This organisation is funded via various cancer charities in Europe, the FP, industry, and the NCI, primarily for clinical trials.

##### The USA

The National Cancer Institute (NCI) in the United States is the main source of cancer research funds in America. The NCI is part of the National Institute of Health and the US Department of Health and Human Resources, established in 1937 as part of the National Cancer Institute Act, and receives its funds from the US Congress [20]. The 2008 budget included a 1% increase from 2007, and 43% was allocated to 5380 Research Project Grants and 15% to intramural research (Table 3.1) [21]. The NCI has a specific drug development platform, which supports a variety of drug development initiatives, from speedier entry into the marketplace to robotic high-throughput screening allowing fast biochemical, genetic and pharmaceutical testing (Box 3.3).

The NCI also supports international research via its International Portfolio, supporting cancer research activity in both developed, transitional and developing countries [22]. The activities range from acting as liaison between entities, supporting training and education, organising global clinical trials and collecting information about experimental drugs and protocols with global collaborators (Box 3.4).

Box 3.4: **Examples of activities supported by the NCI International Portfolio [22]**

- German trial of radioimmunotherapy for NHL, the radioisotope bismuth-213 was provided by the NCI through a Material Transfer Agreement
- National Cancer Institute of Canada (NCIC) participates in the NCI Clinical Trials Cooperative Group Program. Currently, more than 90% of investigational drugs shipped internationally by the NCI Cancer Therapy Evaluation Program are sent to Canada – some of these are included in Phase II trials, the only country to do so outside the United States.
- International clinical trial, led by the NCIC Clinical Trials Group, on letrozole (Femara-Novartis) to reduce the risk of breast cancer recurrence. Patients registered in the trial come from: Canada, the United Staes, United Kingdom, Belgium, Ireland, Italy, Poland, Portugal and Switzerland.
- Collection of more than 50,000 plant specimens from Africa, Madagascar, Central and South America, Southeast Asia for the NCI Developmental Therapeutics Program.
- Support to International Center for Studies of Traditional Chinese Medicine, partnering the University of Texas with Fudan University in Shanghai.
- Support for the AIDS Malignancy Consortium to pursue clinical trials in resource poor

Further support for cancer drug research in the United States is given by the Foundation for the National Institutes of Health, established by the US Congress to support the National Institute of Health via public-private partnership. It is a non-profit organisation raising private funds to support NIH’s public actions, including drug research and development.

##### The United Kingdom

The main cancer research agency in the United Kingdom is the National Cancer Research Institute (NCRI), whose new focus in medicine development is translational research (Box 3.5). The NCRI works with industry, developing relationships and collaboration with private enterprise. A number of companies are involved in various NCRI boards, and the NCRI portfolio now includes 34 clinical trails with 17 different companies with likely increases in the future.

The Medical Research Council (MRC) in the United Kingdom is an additional source of cancer drug development funded by the Department for Innovation, Universities and Skills [23]. The MRC works with the National Institute for Health Research, as well as having close ties with industry, recently increasing expert industry board members. It has recently joined with Cancer Research UK, the Wellcome Trust and University of College London to form the UK CMR International, bringing academics, clinicians and industry together to build a worldclass cancer research institute (building complete 2014) [24]. Their Drug Discovery Group (DDG) includes cancer research as a main focus and includes therapeutic antibodies (Therapeutic Antibody Group) and kinase programme (Protein Phosphorylation Unit) research. They also collaborate internationally with other EU and global cancer research agencies.

##### Canada

In Canada, the two main cancer research bodies are the Canadian Foundation for Innovation and Canadian Institute of Health Research [27]. Together, they fund more than 50% of cancer research, the remainder funded by 33 different agencies, including provincial, federal, voluntary and multi-funded organisations, which together invested $390 million (CDN) in 2006. The Canadian Foundation for Innovation is an independent organisation created by the Government of Canada to fund research infrastructure in order to assist universities, hospitals and non-profit research agencies in performing excellent quality research, including cancer research [28]. One such project is the Canada-California Strategic Innovation Partnership Initiative, a public-private partnership, targeting cancer stem cell research.

Box 3.5: **Examples of UK Cancer Research Activities supported by the NCRI [25,26], a**

Oncology Information exchange (ONIX): launched in July 2009, which allows the public, scientists and physicians to search and access global online research data in order to encourage the flow of cancer research information between vested bodies (£2.5 million, 2003–2010).

National Cancer Research Network (NCRN): supports cancer clinical trials in the National Health Service (NHS) to improve coordination, integration, quality, inclusiveness and speed of cancer research. Currently, 11.2% of cancer patients are now entered into clinical trials, now supported by 33 Cancer Research Networks that are integrated with Cancer Services Network (£150 million, 2001–2011).

Experimental Cancer Medicine Centres (ECMC): aims to expand the experimental cancer medicine portfolio and to attract industry-sponsored experimental cancer medicine. It is funded by the Department of Health and led by Cancer Research UK. It consists of 17 centres, and 2 in development, throughout the United Kingdom, with each centre receiving £2.5 million annually (£35 million, 2007–2012)

Confederation of Cancer Biobanks (onCore UK): systematic collection of cancer biosamples organised by the Department of Health, Cancer Research UK and Medical Research Council (£5 million, 2008).

The Canadian Institute of Health Research is composed of 13 institutes, the Institute for Cancer Research (ICR) being relevant [29]. They support the Canadian Clinical Trials Group, which carries out oncology clinical trials across Canada via 90 member institutions (Phase IIII). In addition, they began the Strategic Training Initiative in Health Research (STIHR), which supports research training as well as cross-collaboration with other disciplines.

##### Germany

The main German cancer research organisation is the German Cancer Research Centre (Deutsches Krebsforschungszentrum, DKFZ), which is the largest biomedical research institute in Germany, and focus on cancer research as well as patient information services [30]. The centre is funded primarily by the government, both federal and state, and donations.

##### France

France has two main cancer research organisations, the National Institute for Cancer (Institut National du Cancer, INCA) and the Association for Cancer Research (Association pour la Rescherche sur le Cancer, L’ARC) [31]. INCA has two main goals to develop cancer expertise and to develop scientific oncology programmes, in order to address public health, quality of care, information dissemination and conduct scientific research [32]. Financing comes from public-private partnerships, government and public sources. L’ARC has supported more than 7500 research projects over the past 6 years, primarily on cellular and clinical research.

##### Other

Other organisations in Europe include the Danish Cancer Society, Swedish Cancer Society, Finnish Cancer Organisation, Spanish Cancer Association, Italian Medical Oncology Association, Norwegian Cancer Society, Swiss Institute for Cancer Research, Dutch Cancer Institute (NKI), and a number of others.

Also, very important to consider are cancer charities, which contribute significant private funds and vision to cancer research. As these monies can be significant, and in many instances greater than national cancer research funding, their role in providing advances in cancer treatment cannot be ignored. In some instances, funds are allocated outside of the home country’s charity, whilst others are allocated only to national research.

##### Overall

The diversity of organisations associated with cancer research is large, often with significant overlap or duplicity in purpose. Most organisations are specific only to a certain country (i.e. Danish Cancer Society) or region within a country, whilst others are true only to specific disease (i.e. Children’s Oncology Group Soft Tissue Sarcoma Committee) even as far as a specific disease in a region of the country (i.e. Quebec Breast Cancer Foundation). It is estimated there are more than 120 cancer organisations in Europe, Canada and the United States that participate partially or solely in cancer research.

Although this diverse representation of research, as well as national or regional, aspects are understandable, the result may be duplication and fragmentation. Administrative costs are duplicated, available funds for similar purposes are split and the cancer research ‘voice’ may be weakened. The Framework Programme in Europe seeks to rectify this somewhat, however, has been criticised for adding yet another layer of cancer research bureaucracy [33,34]. Recently, it has been discussed that EU level leadership on cancer research needs to be improved and strengthened as well as improve partnership with industry in order to foster innovation [35].

Furthermore, differences in national laws can provide a further barrier to performing transnational research [36]. For example, tissue sample banking in one country may be ‘opted in’ whilst another ‘opted out’. Data protection and anonymity is another hurdle, particularly where long-term follow-up is necessary. Even the designation of medical specialties is universal, there are a number of countries without the medical oncology specialty (falling under internal medicine domain). The sparse combination of research and medical speciality (MD, PhD) is also a barrier to cancer research. Although in the United States this combination exists, in Europe this combination is rare.

The EU introduced a Clinical Trials Directive in 2004, designed to harmonise clinical trials in Europe; however, it has been criticised for increasing the bureaucracy and creating another barrier for clinical trials to occur in Europe. In the United Kingdom, it has been held responsible for decreasing clinical trials activity [37], whilst in Europe overall it is a factor in longer trial regulatory procedures [38]. Particularly for childhood cancers, it has been seen as responsible for difficulties in recruitment, increased insurance costs and difficulties between countries in interpretation and direction of trials [39]. It will be reviewed in 2010.

#### (III) Variation in spending by nation

##### (a) Direct cancer research funding

The 153 European public funding organisations spent altogether €2.79 billion on oncology research during 2006–2007. Moreover, the 21 US public funding organisations spent €5.8 billion during the same period (Figure 3.3).

It is, therefore, obvious that the USA dominates in terms of absolute direct expenditure, investing in cancer research more than double the combined investment of European countries. Still, it must be noted that the European figure is likely to be an underestimate as it does not include EU funding by bodies such as the European Commission estimated to have invested on average €90 million per annum during FP6.

The results further suggest that there is a wide variation in direct funding among different nations. In Europe, the highest single source of public funding was the United Kingdom with €1.1 billion expenditure. Germany, France and Italy also invested significant amounts, which when put together, amounted the same as the United Kingdom. From the countries included in the survey only Malta reported no direct investment in cancer research, whilst Bulgaria failed to report on any expenditure.

Another interesting finding is that drug development accounts for a relatively large part of direct public investment (Table 3.2). However, this excludes late stage clinical development and, furthermore, much of this funding is focused on pre-clinical development.

##### (b) Indirect cancer research funding

From previous funding surveys [7] it was clear that, particularly in Europe, significant levels of support for cancer research flow from general taxation into R&D support for hospitals/universities. Through the interrogation of major EU countries and reverse engineering using bibliometrics, the level of this ‘indirect’ funding supporting cancer drug development in the public sector both in Europe and the USA was estimated for 2007 (Figure 3.4).

##### (c) Per capita direct spending for cancer research

Reviewing total public sector direct funding per capita^o^ demonstrated substantial direct funding by the USA (€19/capita) and the United Kingdom (€18/capita) (Figure 3.5). Scandinavian countries and the Netherlands are also strong supporters of cancer research with Sweden, the Netherlands and Norway investing €12.1, €8.8 and €7.2 per capita, respectively. In absolute terms, apart from leading US and UK funding, substantial European funding derives from remaining large Western European countries (Germany, France, Italy and Spain) (Table 3.3).

Regarding drug development, the United States and the United Kingdom remain leaders, both investing more than €5 per capita for drug development. Additionally, eight more European countries spend at least one Euro per capita on drug development (Table 3.4). In absolute terms, large Western European countries dominate in drug development funding as they did in the case of overall cancer research.

The USA and United Kingdom have a long history of public sector support of cancer drug development (Table 3.5). In the USA, this has been in place since the early days of the National Cancer Institute (NCI) and has formed a major backbone of its strategy through a variety of funding streams both direct project related and infrastructural. In the UK cancer drug development in the public sector has enjoyed a major philanthropic backer and, more recently, increased governmental support through the creation of the Experimental Cancer Medicine Centres initiative (ECMC)^p^. However, apart from the USA and the United Kingdom who appear to have a national strategy on cancer drug development, overall the levels of funding per capita do not appear to equate with any coherent national strategy on cancer drug development, rather these levels reflect an *ad hoc* approach by governmental and philanthropic funders.

When averages are examined, it becomes apparent that Europe fails to match the public funding levels of the United States in cancer research and development. Indeed, the average per capita spend on total cancer research across the entire Europe was €3.45 whilst the per capita spent in the United States was €19.34. However, this gap is reduced to threefold if the United States spending is compared with the spending of the EU15 countries only (average per capita spend €5.64).

Compared to 2004 funding levels [1] Europe spend per capita on average in 2007 34.7% more whilst the United States 9.7% more revealing an obvious trend for Europe to close the existing gap with the US (Figure 3.6).

##### (d) Direct spending for cancer research as a percentage of GDP

A similar pattern is evident when one reviews cancer research spend relative to GDP (Figure 3.7). The average cancer research spend for ‘Europe’ was 0.0143% of GDP, which is a decrease of 19.2% from 2004 figures (average spend was 0.0177%). The average cancer research spend in the USA was 0.061% of GDP in 2007, representing an approximate 10% increase more than 2004. The European spend is driven by the United Kingdom (0.072 of GDP on cancer research), followed by Sweden (0.048% of GDP).

In the case of drug development, again the United Kingdom and the United States are investing a larger part of their GDP than the rest of the countries in the survey.

##### (e) Cumulative spending for cancer drug R&D

Total public sector support from all research funding organisations and through hospitals/universities (indirect) identified by this survey for cancer drug development in Europe and the USA was estimated to be €2.8 Billion in 2007/2008 (Figure 3.8).

Adding indirect spend for Europe as a whole dramatically closes the ‘gap’ with the US public sector funding of cancer drug development (Figures 3.9, 3.10). In effect, when both direct and indirect funding is taken into account, Europe spends 0,011% of GDP into cancer drug development compared to 0018% by the USA and €3.64 per capita compared to €5.74 by the USA.

##### (f) Spending by CSO in Europe

In order to compare and contrast research portfolios of public (i.e. NGOs and governmental) research organisations using a certified vocabulary, the Common Scientific Outline (CSO) [40] was used quantify cancer research expenditure. The CSO is a classification system, originally validated by the International Cancer Research Portfolio (ICRP) [41] and now in use by the USA, United Kingdom and Canada [27] that is used to classify spending around seven broad areas of scientific interest in cancer research.

One hundred and two research funding organisations were interrogated across Europe, of which 47 returned usable data of self-reported percentage breakdown of their annual spending on cancer research by the highest common scientific outline level.

Briefly, €750.6 million was spent on cancer research by both charities and governmental research agencies, from which €557.1 million was placed in CSO categories (Figure 3.11).

This dataset found a strong and growing commitment by European funders to supporting cancer drug development. In nearly all projects there was a complex mixture of funding sources supporting various components of the research project, whether laboratory based or clinical. Whilst distinct funding streams do exist from research funding organisations, once these resources hit the front line then research groups and centres utilise both private and public financing in a mixed economy to deliver on the group’s or centres goals.

A high-resolution analysis of all projects falling under cancer drug development (including associated biomarker studies) has also been derived from the ICRP database. Figure 3.11 shows the distribution of active drug development projects (2007/08) by research funder in the USA, United Kingdom and Canada.^q^

In total, these public sector major RFOs in three countries are currently supporting 5105 cancer drug development projects (591 projects by 14 Canadian RFOs; 455 projects by 9 UK RFO’s and 4059 projects by 5 USA RFO’s) (Figure 3.12).

Using the ICRP database, a high-resolution study of the major European Cancer centres involved in drug development has also been conducted to assess Institutional level spend on cancer drug development relative to other domains of cancer research.

Fundamental biology and research into treatment, of which cancer drug development accounts for around 82% of the total spend, dominate the research funding allocations by this sample of major European Cancer Research Centers (Figure 3.13). Research into cancer drug development is a significant aspect of all those centres’ portfolio and across research domains we have estimated from this high-level coding that circa 36% of all cancer research activity in major European Cancer Centers is focused on cancer drug development.

##### (g) Spending by charities versus governments

There are a very large number of charities supporting cancer research worldwide. Some charitable organisations focus on specific types of cancer and give priority in funding research related to this specific cancer. Examples of charities falling into that category included in this survey are ‘Associazione Italiana contro le Leucemie, Linfomi e Mieloma’ and ‘Associazione Italiana per la Lotta al Neuroblastoma’ in Italy, which focus on Leukemia, Lymphoma, Myeloma and Neuroblastoma, ‘Breakthrough Breast Cancer’, ‘Breast Cancer Campaign’ and ‘Leukemia Research Fund’ in the United Kingdom and ‘Prostate Cancer foundation’, ‘The Leukemia and Lymphoma Society’ and ‘The Komen Breast Cancer Foundation’ in the USA.

These charities are usually smaller and do not fund in absolute terms as much as the ones that focus on cancer broadly. For instance, the American Cancer Society, The American Institute for Cancer Research, Cancer Research UK, the Belgian Federation Against Cancer, the Danish Cancer Society, the ‘Association pour la Reserche contre le Cancer’ and the ‘Ligue Nationale contre le Cancer’ in France, the Irish Cancer Society, ‘Associazione Italiana per la Ricera sul Cancro (AIRC) in Italy, the Dutch Cancer Society and the BMBF in Germany are organisations that fund significant amounts in overall cancer research.

In addition, some charities do not focus specifically on cancer as they finance a broad variety of medical research. However, some invest significant amounts on cancer research such as the Wellcome Trust42 that represents the largest charity in the United Kingdom and the ‘Fundacioìn la Caixa’ in Spain.

In the USA, governmental sources of funding for cancer development dominate mostly through the National Cancer Institute (NCI) and NIH Research Project Grant Program (RO1) grants from the National Institutes of Health (NIH). On the other hand, Europe is actively supported by the philanthropic sector.

In this respect, some countries have well-developed philanthropic and governmental funders for public sector drug development both in terms of number of funding organisations and in absolute investment, for example the United Kingdom, the Netherlands, France and Germany. However, most countries are heavily reliant on the private sector to support both individual projects and some basic infrastructure within the hospitals/universities sector.

In particular, whilst philanthropic funders support a wide range of cancer drug development projects, there appears to be a relative deficit of funding from governmental sources in Europe (Figure 3.14). In fact, more than 50% of non-commercial funding in Europe derives from the philanthropic sector.

It should also be mentioned that in Europe, for six countries (i.e. Bulgaria, Estonia, Latvia, Lithuania, Romania and Slovenia), there were no philanthropic funding organisations for oncology research. In contrast, in Cyprus, there was no government R&D funding organisation for cancer.

##### (h) Spending by political EU membership status

Current European Union member states (EU27) contributed more than €2693 million to non-commercial cancer research. The accession countries contributed only for the 0.75% of the total expenditure in Europe. The European Commission (EC), according to the per annum average during FP6, contributed €90 million to cancer research, although this number may be underestimating the actual support as cancer research is being funded by other, indirect streams from the EC (Figure 3.15).

### 3.3.2. Private sector cancer funding organisations

#### [I] Types of private funding for cancer research

Private/commercial funding for cancer research derives from the industry (Pharmaceutical and Biomedical/Biopharmaceutical Industry). Information on industry-related funding is difficult to determine partly because of proprietary concerns. Moreover, it is said that marketing and administration costs are often included into the reported R&D expenditure [8]. The private funding focuses primarily on drug development [43].

#### [II] Commercial expenditure on cancer research

Various publications have estimated commercial expenditure on cancer R&D using a variety of sources. In general, there are three different ways to calculate worldwide private R&D spending:

1. Based on development cost of a new chemical entity (NCE).
2. Based on total R&D expenditure weighted by share of oncology drugs.
3. Total oncology and immunomodulant R&D expenditure (e.g. according to CMR International).

Because the variety of accounting methods used as well as the inclusion of ‘marketing’ expenditure as part of these estimates. it has been notoriously difficult to estimate the true level of disease-specific expenditure.

One novel way to estimate direct cancer-related expenditure using bibliometrics is by assigning cost per unit of research based on outputs, that is the proportion of each company’s published papers that are in cancer research (Figure 3.16).

Stated R&D expenditure from annual reports is considered to be the total R&D cost for each major pharmaceutical company. However, one issue with this method is that it is likely to underestimate the true activity of a company in certain areas from which publications do not adequately reflect output, for example early phase clinical trials. We can correct for this relative underestimation to give a broad picture of direct spend on cancer drug R&D.

Total expenditure by 17 major pharmaceutical companies was almost €3095 million (2004 figures, reported in 2008). By separating the US-based from the Europe-based companies, we can get an idea of what proportion of private spending flows from each area (Figure 3.17). In fact, 59% of the total private cancer R&D funding derived from Europe-based companies.

### 3.3.3. Public-private partnerships in cancer drug development

Public-private partnership is strong in both Europe and the USA, particularly in the former this is growing. Philanthropic and federal funders must recognise and reward these joint enterprises, particularly in light of the growing complexity and number of the cancer drug pipeline and associated predictive markers.

The figure below (Figure 3.18) gives a ‘snapshot’ of recent annual spending from which we can assess the relative amount of public-private collaboration. Although this only looks at one metric (i.e. outputs), it nevertheless provides an indicator of the current health of private-public collaboration in cancer drug development. These measure real collaborations, that is joint intellectual input, but do not take into account projects for which industry have provided basic infrastructure funding to research units, unrestricted educational grants or in-kind support (e.g. free-drug supply). All these activities could legitimately be put into the public-private ‘partnership’ category. Furthermore, an analysis of the projects detailed in Figure 3.12 has also been used to estimate the degree to which industry and the public sector already cooperate on cancer drug development.

Looking at trends, whilst the USA and the Rest of the World have been relatively static since the mid 1990s, Europe has seen substantial growth in its private-public joint research projects on cancer drugs. We have also found some evidence (from interviews with key opinion leaders) to support the fact that the practice of unrestricted grant support appears a more widespread practice in Europe than the USA.

## 3.4. Discussion

The burden of cancer has broad consequences that go beyond individuals and their families to the health care systems and economies of individual countries. Therefore, as human lives are increasingly burdened by cancer, the fight against the disease relies on both a strong privately and publicly funded research base.

Understanding public and private spending on cancer drug research and development, either to support direct research costs or through general infrastructural funding, is essential to build a coherent picture of the long-term health and stability of cancer research and to understand the future of cancer drug development. Any public policy decisions regarding the setting of priorities and investment in cancer research need to be anchored in evidence, and one of the necessary key understandings is the source and flow of funds to support research presented in the previous section. Moreover, comparable and reliable data on ongoing research activity are essential in order to promote and coordinate strategic planning of cancer research both at national and international levels.

This section discusses the motives behind public cancer research funding and identifies the emerging trends in terms of overall ‘financial commitment’ to cancer drug development research. In parallel, it explores how the sources and sinks of funding compare between countries and whether there are policy learning points from those deemed as successful. Moreover, the role of the private sector, its partnership with the public sector, and the national/supranational interface are underlined. Finally, it discusses whether a clear picture is emerging in forming policy options by highlighting the lack of data, the gaps and the limitations in our understanding of cancer research funding.

### 3.4.1. Public sector funding

#### [I] Motivation for publicly funded research

In general, there are two main kinds of motivation for publicly funded research:

The first is the *economic motivation* that derives from the market failures that accompany the production of new knowledge. Specifically, the difficulty in establishing property rights within the process of producing scientific and technological advancements makes the engagement to such research unattractive to profit-seeking investments [44,45]. Simply put the probability of spillover effects along with the high risk of failure and the timely process of creating knowledge are often responsible that basic research is often a poor target for private investors.

On the other hand, **micro**-level empirical evidence [46–50] has shown that the public rate of return on investment in research to produce such knowledge tends to be many multiples of the rates for private investors due to positive spillovers to consumers and competitors. **Macro**-level evidence [51,52] suggests that the growth rate of the economy’s productivity is associated with the creation and adoption of innovative technology.

Consequently, private investment in types of scientific R&D, such as medical and biomedical research, is not expected to reach the optimal societal level and therefore, public contribution is necessary. One form of intervention is the establishment of firm intellectual property rights in the form of patents, copyrights and market exclusivity periods. Tax subsidies of private research are also common, but such instruments may often be ineffective as it is hard to target basic-discovery research for which there is underinvestment unless such incentives are targeted accordingly. As a result, applied development research that could anyway attract enough private funds also winds up being subsidised.

The above discussion leads to the conclusion that the most reliable tool to assure socially beneficial levels of R&D is direct funding. Long-term funding programmes could also contribute to the stability of research levels regardless of business cycles in industry or the economy. In addition, provision of research infrastructure and high-quality training for the next generation of scientists could lead to further beneficial effects [53].

The other type of motivation for public funding of research stems from *political and social* reasons. Historically, it has been more likely that political concerns such as national esteem and defence agenda have been the key incentives of public funding for scientific research rather than economic ones. Furthermore, social needs have been another catalysing driver of public investment in R&D. Diseases such as cancer affect millions of people every year, place a significant burden on society and, in consequence, the public demand for the alleviation of such disease-related burden puts a lot of pressure on governments.

A good example is the NIH in the United States that has been the key player in the fundamental progress of bioscience, representing the public motivations through its system of 25 institutes organised around ‘body systems’ (such as the National Lung Institute), and illnesses (such as the National Cancer Institute). The proportions of the NIH budget devoted to each health condition reflect that condition’s relative threat to the US nation. Still, in harmony with Vannevar Bush’s vision (Box 3.1), an extensive peer review system is employed to allocate each institute’s resources to researchers and research projects (Box 3.6). Bush’s vision of course is just one of the many different ways the US government can and does fund scientific research, as shown in Figure 3.19.

Box 3.6 **The Vannevar Bush vision of national science policy**

- In 1941, Vannevar Bush, former Dean of Massachusetts Institute of Technology School of Engineering and president of the Carnegie Foundation, was assigned the management of the Office of Scientific Research and Development by President Roosevelt. Bush successfully coordinated the application of academic knowledge to support the war effort leading – among other – to the mass production of penicillin, the manufacturing of a feasible synthetic rubber and the atomic bomb.
- In an attempt to assure the harnessing of such results in the post-war era, Vannevar Bush described in his influential Carnegie Foundation report ‘Science: The Endless Frontier’ the establishment of a permanent federal science policy.
- According to his vision, basic scientific research including medicine, physical science and life sciences would be carried out by both state and academic scientists and would be supported by federal funds.
- Bush rejected ‘economic’ motivation and adopted the humanistic argument that excessive political or commercial direction of scientific research would inhibit the scientists’ curiosity motives, which he believed to be the most effective. Thus, he proposed that scientists would be in charge of the fund allocation through the establishment a peer review system.
- To date, the purest application of his ideal is considered the National Science Foundation, founded in 1950. However, the NHI is itself an example of an – at least partial – application of his vision.

***Source:*** [53,54].

#### [II] National and supra-national spending

The USA has a well-established national cancer strategy, making the USA the largest single source of funds invested in cancer research globally (Box 3.7). The direct public sector spending in 2007 was €5.8 billion, whilst indirect public spending was estimated at €477 million. In addition, US-based pharmaceutical companies invested €1.3 billion in cancer R&D. All these figures together result in €7.06 billion of cancer research funding in 2007.

In Europe, the public sector invested directly €2.79 billion in cancer research in 2007. Adding to that the €90 million of European Commission Funding, the €1.8 billion spent on cancer R&D by Europe-based pharmaceutical firms and taking into account the indirect funding of €1.6 billion that flows through hospitals/universities, it is clear that a strategic plan to coordinate the allocation of these significant funds is indispensable. The above figures result in a grand total of €6.28 billion for cancer research funding in 2007, which is comparable (although slightly inferior) to the sum invested in the USA.

The findings of this survey brought to the surface three major facts:

First, there are prevailing gaps in funding between Europe and USA as well as among European countries and especially between EU Member States themselves. In Europe, most of the funds are raised and invested within EU15 Member States. Particularly, the United Kingdom, Germany, France and Italy dominate cancer research investment in absolute terms. Yet, in terms of spend per capita or spend as a percentage of GDP, apart from the United Kingdom that supports cancer R&D with outstanding amounts of funds (Box 3.8), the other major funders were Sweden and the Netherlands.

The disparities in national spending are even more apparent in the case of the new Member States that joined the EU during the 2004 and 2007 enlargements. These countries limit their activities primarily to prevention and awareness programmes (e.g. tobacco control). The need for further political commitment on cancer research as well as the development of a specific cancer policy framework is highlighted in order for the whole enlarged Europe to meet – even with some delay – the target of increasing science and technology research spending to 3% of the EU’s GDP set at the Lisbon European Council of March 2000 [56].

On the other hand, countries such as the USA, the United Kingdom and Sweden spend a significant proportion of their GDP in cancer research (i.e. 0.061%, 0.072% and 0.048% of GDP, respectively), which demonstrates that cancer ranks highly among their sociopolitical priorities. Despite the discrepancies among European nations, Europe on average increased in 2007 the per capita investment in cancer research by 34.7% since 2004, compared to a just 9.7% increase by the USA, illustrating a desire for Europe to close the existing gap with the USA.

Second, the weight that nations place on cancer drug development and, therefore, into more applied clinical research, varies significantly. The evidence suggests that public funding primarily focuses on basic rather than applied research, as the latter is more likely to attract private funds.

The third issue has to do with cancer research funding at the EU level. The average annual spend was estimated at €90 million during FP6. It is probably the case that this amount has been insufficient to cover the research needs of cancer^r^ [57]. However, FP7 (2007/2013) has already been launched and with a cancer research budget set at €5,984 million [58], it is expected to improve both the existing cancer research effort and its clinical applications.

Box 3.7 **Public Funding Sources for Cancer Research in the United States**

In 1971, the National Cancer Act launched the War on Cancer, a disease that apart from causing suffering and deaths among the Americans also has significant economic costs. The War on Cancer greatly amplified the priority of cancer research in the federal budget, and the Act set up a model of public-private collaboration built around a national net of research laboratories and cancer centres.

The advances in cancer research that followed led to improved screening methods and better treatments, which along with the greater understanding of risk factors such as smoking, resulted in bringing to an end the increasing rate of cancer mortality in 1991.

Today, the USA leads cancer research and drug development and is the largest single funding source in the world. Indeed, in 2007 €5.8 billion were directly invested in cancer research just by the non-commercial sector, reflecting the USA’s well-established national cancer strategy.

Most of the funding for cancer research derives from *federal sources*:

- ***The National Cancer Institute*** (NCI), established in 1937, is the first categorical institute of the National Institutes of Health and also its biggest one. Although the NCI is not the only NIH funder of cancer research, it is the largest one and almost all funds dedicated to cancer R&D derive from it. Its annual strategic research and academic research are openly published but NCI also supports drug development research. The NCI’s role in R&D includes: (1) the integration of discovery activities through inter-disciplinary partnerships; (2) the speeding up of innovations and provision of technology that will facilitate accomplishments of translational research and (3) the measuring of the application of knowledge deriving from these innovations in cancer care, for instance in clinics or public health programmes.
- ***Other National Institutes of Health*** also support cancer research. The National Lung and Blood Institute, for example contribute through its agendas for lung and blood diseases respectively. The National Institute’s of Environmental Health Sciences research on biological responses to environmental agents also includes cancer. As cancer concerns all organs and systems and every age group, each NHI institute and the majority of centres contribute to some cancer-related research.
- ***Other Department of Health and Human Services Agencies*** (DHHS) such as the Centers for Disease Control and Prevention (CDC), the Agency for Healthcare Research and Quality (AHCPR) and the Health Care Financing Administration (HCFA) spend significant amounts on cancer-related research.
- ***Other Federal Agencies*** also fund cancer R&D projects. For example, the Department of Defense (DOD) has become a major source of funds in recent years for research on breast, ovarian and prostate cancer. Moreover, the Department’s of Veterans Affairs (VA) research initiatives focus on cancer, among other on chronic diseases and conditions that affect veterans. Agencies such as the Environmental Protection Agency (EPA), the Department of Energy (DOE), NASA, the National Science Foundation (NSF) and Department of Agriculture (USDA) also fund some cancer research, but their contribution is relatively small.

Public funding also streams from *non-profit organisations* such as independent endowments and funds, corporate giving foundations and community-based donors. Funding deriving from such organisations is tracked by the Foundation Center.

- The ***Howard Hughes Medical Institute*** is a strong supporter of the biomedical research and especially cancer-related research.
- ***Voluntary Health organisations*** such as the American Cancer Society (ACS), the American Institute for Cancer Research (AICR), the Cancer Research Foundation of America and the National Foundation for Cancer Research support the fight against cancer by collecting donations from the general public and by funding apart from basic cancer research, treatment services and prevention programs. Similarly, charities that focus on specific types of cancer such as the Leukemia Society of America (LSA), the Association for the Cure of the Cancer of the Prostate (CaP CURE) and the Komen Breast Cancer Foundation, contribute a great deal in cancer research projects.

Finally, several *State Governments* directly support financially cancer centres or have established cancer research programs.

*Source:* Sources of Research Funding in the US/concerning Trends and Outcomes for National Institutes of Health Funding of Cancer Research

During the FP6 and the first two calls of FP7, around €750 million was allocated to cancer research. On 26 June 2009, an EC Communication underlined that most research in the field of cancer is carried out at Member State level, and hence, is ‘fragmented’ [59]. In this direction, the Communication proposed an ‘European Partnership for Action against Cancer’, which apart from public health measures (e.g. screening) and good treatment practices recommends additional coordination and collaboration in cancer research. With the objective to achieve coordination of one third of cancer research from all funding sources by 2013, the Commission proposed the creation of a large stakeholder forum to undertake the work of the Partnership that will include all kinds of organisations, in the 3rd quarter of 2009. The stakeholder working groups will be based on the following areas of action: prevention, healthcare, research and information, and their work will be coordinated by a ‘steering’ group.

As part of the governmental funding is indirect (i.e. flows through hospitals/universities) although used for cancer research, it is not clearly earmarked. In consequence, this survey primarily addresses direct cancer research investment and, therefore, there may be underestimates of the level of indirect contributions. Moreover, there are likely further underestimates of the contribution of several smaller charities with annual spending of less than one million euros. For instance, it has been estimated that more than 1500 such charities are operating in EU15 alone. Furthermore, the contribution of European umbrella bodies (e.g. the Federation of European Cancer Societies, FECS), relevant patient groups and policy initiatives for cancer research such as EUSTIR and EURoCAN+Plus^s^ that have some involvement in cancer research, has also been underestimated.

#### [III] Role of indirect and philanthropic funding

In the USA, the NCI alone spent €3.58 billion on cancer research in 2007, an amount accounting for 62% of the total direct public sector spent in the USA. This is reflecting the fact that for the USA cancer research is a long-established priority, supported mainly by a centralist financing model. On the other hand, in Europe, cancer research represents a different priority for different countries, whilst its funding is widespread across numerous diverse funding sources.

Specifically, in Europe, significant funding supports indirectly cancer research flowing through academia and health care systems (e.g. only 3.2% of cancer drug development research comes from indirect sources in the USA, compared to 44.7% for Europe). Hence, taking additional indirect investment into account, Europe as a whole considerably closes the gap with US public sector funding of cancer drug development (i.e. 0.011% of European GDP goes into cancer drug development compared to 0.018% of the USA GDP).

Box 3.8 **Cancer research funding in the United Kingdom**

Cancer is major health condition in the United Kingdom and as the population ages and lives longer; it is becoming increasingly a greater burden to the National Health System (NHS).

In 2007, the United Kingdom’s non-commercial sector invested €1.1 million in cancer research, an amount that corresponds to more than one third of Europe’s overall investment. This is consistent with the fact that cancer has long been a national priority for the United Kingdom.

In the United Kingdom, cancer research is supported by several different *government agencies*, most of which fund medical research in general, rather than having a cancer-specific focus. The most important funders among these include the *Medical Research Council* (MRC)^1^, the Department of Health (DoH), the Biotechnology and Biological Sciences Research Council (BBSRC), The Economic and Social Research Council (ESRC), the Northern Ireland HPSS R&D and the Wales Office of R&D.

Furthermore, what distinguishes the United Kingdom from most other countries is the fact that it has an exceptionally large *charity sector*. We have indeed estimated that about 250 different charities support cancer research in the United Kingdom:

- ***Cancer Research UK****2*, the result of merging the Imperial Cancer Research Fund and the Cancer Research Campaign, is the largest non-government cancer research organisation worldwide. Cancer Research UK collaborates with other charities, public research organisations and the pharmaceutical industry to better understand the disease, to improve prevention, diagnosis and treatment of cancer and to keep cancer a top priority of the national health agenda. During 2007–2008, Cancer Research UK raised £477 million and spent on cancer research a record of £333 million.
- Other important NGO’s that invest at least £1 million on cancer research in the United Kingdom include: the Association for International Cancer Research, the Breakthrough Breast Cancer, the Leukemia Research Fund, the Ludwig Institute for Cancer Research, the Macmillan Cancer Support, the Marie Curie Cancer Care, Tenovus, Roy Castle Lung Cancer Foundation, Children with Leukemia and Yorkshire Cancer Research .

As a response to the need for coordination of cancer research and collaboration among the numerous governmental, non-governmental and private sector funders, the *National Cancer Research Institute* (NCRI)^3^ was founded in 2001. NCRI brings together the 20 largest of the charity and government funders, along with industry represented by the Association of the British Pharmaceutical Industry (ABPI). NCRI’s role consists in maintaining a strategic overview of cancer research in the United Kingdom and in encouraging greater consistency among the major funders. In order to do so, NCRI maintains a Cancer Research Database, develops research schemes, assists the organisation of clinical trials on experimental cancer drugs and develops research facilities and resources (e.g. data management using IT).

Furthermore, a great deal of cancer research relies on charitable organisations’ support in Europe. Indeed, direct non-commercial spend is almost equally shared between governmental (€298.7 million) and philanthropic (€301 million) organisations. In contrast, in the USA only 13.76% of non-commercial cancer research comes directly from charitable funders.

For some EU countries (e.g. Sweden and Hungary) the fact that this balance does not exist and charitable funding is dominating reveals the different philanthropy patterns that exist among countries 60. Charitable fundraising relies on altruism, often associated with uncertainty, but is quite significant in some cases and is an additional source to governmental and industry funding [61]. Ideally, there could be a closer collaboration and coordination between governmental and charitable funders. Although a politically challenging goal [62], steps towards establishing this funding model have already been established both in the USA and in Europe with the founding of ‘umbrella’ organisations such as the C-Change^t^ (USA), the NCRI (UK) and INCa (France).^u^

As the strategies of the charitable organisations were beyond the scope of this review, the possibility that several fundraising organisations supporting cancer care delivery may not have been taken into account exists. Moreover, EU Commission funding was not included in the calculations of cancer research spending for Europe as a whole neither in absolute terms, nor per capita, nor as a percentage of GDP. Therefore, it is likely that the gap between Europe and USA is overestimated.

#### [IV] CSO coding system: National strategic planning with international context

In order to ensure progress in cancer research, international cooperation is essential. Recognising that, in September 2000, several international cancer funding organisations decided to use a common scientific outline, known as the Common Scientific Outline (CSO), to code their research portfolios. The Initial Cancer Research (ICR) Partners, formerly known as the CSO Partners, were US and UK organisations such as the US NCI and Cancer Research UK. In 2007, the Canadian Cancer Research Alliance (CCRA), which represents key government and non-governmental cancer funding organisations, joined the ICR Partners.^v^

The CSO makes it possible to compare and contrast the research portfolios of multiple organisations and provides the information needed to improve coordination among research organisations. To facilitate information sharing between ICR Partners, the International Cancer Research Portfolio (ICRP), an information database on ongoing cancer research funded by the ICR Partners was created. The ICRP public website can provide contacts for multidisciplinary research and partnerships between researchers conducting similar work, in addition to inform internal and joint policies of cancer funding organisations and government/policy officials and to set directions for future research efforts.

### 3.4.2. Private sector funding

Private (i.e. commercial) sector cancer research funders, which are represented by the pharmaceutical and the biotechnology industries, are estimated to invest around €6 billion annually worldwide [63], an amount that accounts for one quarter of total global research expenditure and that is mainly invested in the USA and Europe [64].

Pharmaceutical companies seem to account for the great majority of total private expenditure. Indeed, the research budget of the pharmaceutical firms has increased steeply (approximately by 13% annually) since 1970 [65]. Furthermore, it has been estimated in previous studies, using 2004 data, that 7% to 12% of the total industry R&D expenditure is dedicated to cancer research [7,50]. Taking into account that oncology drug sales account for 5% of total drug sales and that 15% of total cancer drug sales are reinvested in R&D compared to 10% of global drug sales, the increasing interest of pharmaceutical manufacturers to invest in oncology is further reflected [19].

Despite the increase of R&D expenditures across the industry, the amount of New Drug Applications (NDAs) has stayed flat during the 30 last years, reflecting an almost 10-fold drop in R&D productivity [51,53]. The recent overall drop in the number of New Chemical Entities (NCEs) approved by the US Food and Drug Administration (FDA) each year is a key challenge for the pharmaceutical industry [7]. Given also that many patents are about to expire, the problem becomes bigger, as many firms do not have adequate effective new medicines to balance this loss [56].

Traditionally, large pharmaceutical manufacturers used to conduct in-house discovery research, designed their own clinical trials, manufactured their own drugs and were in charge for their sales and distribution channels [66]. In response to the challenges discussed above, the industry has recently experienced a major transformation. A growing number of new drugs derive from in-licensing rather than in-house research. For instance in 2002, 40% of the big pharmaceutical companies’ pipelines originated from in-licensed resources, in contrast to merely 16% in 1980 [67]. This highlights the changing dynamics in R&D and, probably, also underscores the need for a different R&D model that makes better use of technological advances from a wider pool of knowledge.

Box 3.9 **Cancer research through Public-Private Collaboration in the USA**

- ***Big biopharmaceutical companies*** are the principal source of R&D funding for innovative drugs, both for projects in their own laboratories as well as for research licensed from other sectors.
- ***Smaller companies*** also drive innovation, conducting basic research, drug discovery, pre-clinical experiments and, in some cases, clinical trials.
- The ***National Institutes of Health*** (NIH) provides leadership and funding support to universities, medical schools, research centres and other non-profit institutions and stimulates basic research and early stage development of technologies that enable further targeted drug discovery and development.
- The ***National Cancer Institute*** (NCI) often seeks ***Cooperative Research and Development Agreements (CRADAs)*** with pharmaceutical or biotechnology companies. The goals of the CRADAs commonly include the rapid publication of research results and timely commercialisation of products, diagnostics and treatments resulting from the research. The CRADA Collaborators usually have the option to negotiate the terms of an exclusive or nonexclusive commercialisation license to subject inventions arising under the CRADAs.

*Sources:* See [14] in Chapter 1.

Cooperative Research and Development Agreements (CRADAs): http://www.usbr.gov/research/techtransfer/together/crada/whatcrada.html

The fact that increasingly big pharmaceutical companies invest in other start-up biomedical/biotechnology companies is also an interesting trend (e.g. the Novartis Venture Fund was launched in 1996 and Lilly Ventures in 2001) [68]. The finding that key venture investment prospects keep emerging when the vertically integrated pharmaceutical industry is considered from the perspective of the potential of developing large horizontal players, can explain this trend [57].

Comparing Europe’s private sector to USA, it seems that when the geographical origin of industry’s publications is taken into account, the cancer research activity between the two areas is balanced. Indeed, Europe accounted for 45.9% of total pharmaceutical cancer research spend in 2004, despite the established opinion that Europe is weaker in attracting industry research funds [51].

Finally, it must be noted that the significant contribution of the private sector may be underestimated in this review due to the lack of direct industry data on cancer research spend and our approximation through bibliometric exercises. In addition, data were limited to major pharmaceutical companies, and despite the fact that they account for the overwhelming majority of private funding, the contribution of small and medium firms as well as biotechnology companies was not taken into account.

### 3.4.3. PPPs in cancer drug development

Modern drug discovery has become the product of collaboration. Many sectors contribute, particularly in building the basic science foundations, as both public and private organisations play unique but increasingly interdependent roles in translating basic research into medicine (Box 3.9).

#### [I] Industry and academia collaboration against cancer

Increasingly research policy has been directed to supporting the transfer of technology from knowledge generating organisations in the public sector (e.g. universities) to firms through the establishment of cooperative links. Rather than feeling challenged, the industry welcomes academic contribution in identifying and validating early drug targets, as this can lead to reduction of corporate risk and to a smoother drug development operational process. The potential of closer collaboration among universities, biotechnology and pharmaceutical companies, could lead to universities conducting basic research, biotechnology firms developing technology and chemistry, whilst pharmaceutical firms would be in charge of developing clinical trials. Hence, it is not surprising that discovery centres have been established close to major academic research hubs. For instance, Novartis and Merck funded in 2002 drug discovery centres in Boston, USA, not far from the Harvard and MIT Universities [69,70].

#### [II] Integration of public and private investment

As a result of the high sociopolitical priority given to health by countries such as the USA, the United Kingdom and Sweden, total research and development budgets are increasing whilst emphasising cost-effective innovations. At the same time, the industry is more and more utilising research funded directly from public sources and cooperating with public research institutions [71].

Over half of the significant cancer research activity conducted both in the USA and in Europe is the product of partnerships with the public sector, a trend that has been increasing [53]. In fact, recently, the majority of cancer research funding policies have accentuated the public-private partnership path. However, as EU funds are often being joined with industry, there are concerns that ‘if all increases in EU cancer research funding go this way Europe’s intrinsic creativity would be distorted by encouraging subsidy-seeking behaviour and essential areas of public health relevant to cancer, but not amenable to a business approach would remain orphans’ [53].

## 3.5. Concluding Remarks

Cancer drug research and development is a multi-faceted activity that seeks to relieve the burden of more than 15 million people estimated to face the disease by 2020. It comprises not only the discovery and development of new drugs but also in the enhancement of current therapies, the improvement patient quality of life and the prevention of the disease.

As research activity and high-quality health care delivery are linked, intensive research and drug development is likely to have a positive effect on the overall care, and therefore, the quality of life of cancer patients. Triggering cancer research funding and creating the necessary infrastructure to conduct research can also make a country more attractive to research and contribute to the prevention and likely reversal of ‘brain drain’.

This survey identified 174 major public funding sources across Europe and USA, which were estimated to have invested €8.6 billion in cancer research in 2007. Adding in the private sector contributions, that are estimated to be in the region of €6 billion, it becomes clear that significant amounts are spent on cancer research. Still, the private sector’s contribution is both an approximation and an underestimate as important components could not be included due to lack of data.

Whilst the contribution of the non-profit philanthropic sector is growing, some EU Member States still fail to provide adequate governmental funding. This is of some concern in the case of countries that can afford higher levels of cancer research funding and that have the required research workforce.

Public-private partnerships are also strong and growing in both Europe and the USA, and public funders should recognise and support these joint efforts, given the increasing complexity and quantity of the oncology drug pipeline.

However, difficult policy decisions need to be made on what research will be funded given the need to prioritise and optimise resources.

Despite the likelihood of any omissions and over- or underestimations, this section has mapped the diverse funding sources of cancer R&D and underlined that bureaucracy is often a major threat to this complex, multi-faceted, but important effort. In the face of the steps that have been taken recently in promoting cancer research, more countries need to recognise cancer prevention as a high national priority and to engage in international collaboration and coordination strategies.

## Figures and Tables

**Figure 3.1:** **The public-private interface in cancer drug research and development.** ***Source:*** The authors.

**Figure 3.2:** **Number of public sector funding organisations by country.** ***Source:*** The authors.

**Figure 3.3:** **Direct cancer drug R&D (Spending on log scale).** ***Source:*** The authors.

**Figure 3.4:** Estimated indirect cancer R&D funding.

**Figure 3.5:** **Cancer R&D direct spending (€) per capita.** ***Source:*** The authors.

**Figure 3.6:** **Direct cancer R&D spending per capita, 2004 versus 2007 (€).** ***Source:*** ECRM (2004 data) and the Authors (2007 data).

**Figure 3.7:** **Cancer drug R&D and cancer R&D direct spending, % of GDP.** ***Source:*** The authors.

**Figure 3.8:** **Route of cancer drug R&D funding.** ***Source:*** The authors.

**Figure 3.9:** **Cancer drug R&D spend per capita (€).** ***Source:*** The authors.

**Figure 3.10:** **Cancer drug R&D spend, % of GDP.** ***Source:*** The authors.

**Figure 3.11:** **European cancer R&D spend by CSO category.** ***Source:*** The authors.

**Figure 3.12:** **Number of active cancer drug development projects by funder, grouped by country (log scale).** ***Source:*** The authors.

**Figure 3.13:** **Scatter plot showing percentage of spend according to domain of research (by CSO category) for a sample of European Cancer Centres (*n*= 20).** ***Source:*** The authors.

**Figure 3.14:** **Drug R&D funding by charities and government (2007).** ***Source:*** The authors.

**Figure 3.15:** **Percent of direct spend by political group in Europe (2007).** ***Source:*** The authors.

**Figure 3.16:** Private cancer drug development spend: major pharmaceutical companies (2004/ Phase III)* *Note:* *The figure does not include all industry (e.g. SME and biotechnology). ***Source:*** [7].

**Figure 3.17:** **Private cancer R&D funding by company origin.** ***Source:*** The authors.

**Figure 3.18:** **Cancer drug development projects with joint private-public funding (%) (2007–2008).** ***Source:*** The authors.

**Figure 3.19:** **Interaction between basic versus applied science and between market versus government.** *Source:* Adapted from [**51**].

**Table 3.1:** Synopsis of policies and programmes encouraging cancer drug development globally

**Table 3.2:** Drug R&D as percentage of total direct spending

***Source:*** The authors.

**Table 3.3:** Top-10 public spenders on cancer R&D per capita versus absolute spending

***Source:*** The authors.

**Table 3.4:** Top-10 public spenders on cancer drug R&D per capita versus absolute spend

***Source:*** The authors.

**Table 3.5:** Main funding sources of Europe’s top funding countries (public sector)

***Source:*** The authors from National Sources.

## Footnotes

1 See: www.mrc.gov.uk

2 See: www.cancerresearchuk.org

3 See: www.ncri.org.uk

## References

[1] DiMasi JA, Hansen RW, Grabowski HG (2003). The price of innovation: new estimates of drug development costs. J Health Econ.

[2] DiMasi JA (1995). R&D costs for new drugs by therapeutic category: a study of the US pharmaceutical industry. PharmacoEconomics.

[3] Adams CP, Brantner VV (2006). Estimating the cost of new drug development: is it really 802 million dollars?. Health Aff (Millwood).

[4] (1997). Report on the Sources of Cancer Research Funding in the United States, prepared for the National Cancer Policy Board, Institute of Medicine.

[5] UK Clinical Research Collaboration http://www.ukcrc.org/PDF/UKCRC_Health_Research_Analysis_May_06.pdf.

[6] Eckhouse S, Lewison G, Sullivan R (2008). Trends in the global funding and activity of cancer research. Mol Oncol.

[7] Eckhouse S, Lewison G, Sullivan R (2007). Investment and Outputs of Cancer Research: from the Public Sector to Industry/The Second Cancer Research Funding Survey. European Cancer Research Managers Forum.

[8] McGeary M, Burstein M (1999). Sources Of Cancer Research Funding in the United States. Prepared for the National Cancer Policy Board.

[9] See https://radius.rand.org

[10] Combating cancer http://cordis.europa.eu/lifescihealth/cancer/home.htm.

[11] (2009). Action against cancer: a European Partnership briefing.

[12] EC (2008). Cancer Research: projects funded under the 6th Framework Programme.

[13] EurocanPlus (2008). Feasibility study for coordination of national cancer research activities. Summary Report.

[14] IMI http://imi.europa.eu/index_en.html.

[15] EFPIA Press Release (2009). IMI: €246 million to support PP research cooperation for a fast development of better medicines. Brussels.

[16] IMI Research Agenda (2008). Creating biomedical R&D leadership for Europe to benefit patients and society.

[17] See http://ec.europa.eu/research/fp7/index_en.cfm?pg=health

[18] www.rarecancers.eu. Accessed Aug 2009

[19] McCabe C, Bergmann L, Bosanquet N (2009). Market and patient access to new oncology products in Europe: a current, multidisciplinary perspective. Annals Oncol.

[20] NCI FactSheet (2009). Cancer research funding. Version 1.1.

[21] NCI Fiscal Year 2008 Annual Budget

[22] NCI (2006). Addressing the global challenge to cancer. NIH.

[23] Medical Research Council (2008). Annual report and accounts, 2007–2008. House of Commons.

[24] CMR International

[25] National Cancer Research Institute (2008). NCRI Strategic Plan, 2008–2013.

[26] NCRI Analysis of Cancer Research Portfolio, 2002–2006.

[27] Canadian Cancer Research Alliance (2008). Cancer research investment in Canada, 2006.

[28] Canadian Foundation for Innovation (2008). Annual Report, 2007–2008.

[29] CIHR – ICR http://www.cihr-irsc.gc.ca/e/12407.html.

[30] DKFZ http://www.dkfz.de/en/index.html.

[31] Association for Cancer Research (2008). Annual Report, 2008.

[32] National Institute for Cancer (2008). Annual Report.

[33] Fricker J (2007). Cancer research funding in Europe. Mol. Oncol.

[34] Sullivan R (2007). The good, the bad, and the ugly: effect of regulations on cancer research. Lancet Oncol.

[35] Andrejevs G, Celis JE, Guidi G (2009). Tackling cancer in the EU: The role of innovation. Mol. Oncol.

[36] Fricker J Translational cancer research in Europe. Mol. Oncol.

[37] Gulland A (2009). Number of global clinical trials done in UK fell by two thirds after EU directive. BMJ.

[38] Heerspick L, Dobre D, Hillege HL (2008). Does the European clinical trials directive really improve clinical trial approval time. Br J Clin Pharmacol.

[39] Pritchard-Jones K (2008). SIOP Europe. Clinical trials for children with cancer in Europe – still a long way from harmonization. A report from SIOP Europe. Eur J Cancer.

[40] Common Scientific Outline http://researchportfolio.cancer.gov/cso.html.

[41] Common Scientific Outline, International Cancer Research Portfolio http://www.cancerportfolio.org/index.jsp.

[42] Wellcome Trust http://www.wellcome.ac.uk/.

[43] Lengauer C, Diaz LA, Saha S (2005). Cancer drug discovery through collaboration. Nature Reviews, Drug Discovery.

[44] Nelson RR (1959). The simple economics of basic scientific research. J Pol Econ.

[45] Arrow KJ (1962). Economic Welfare and the Allocation of Resources fir Invention” in the “Rate and Direction of Inventive Activity”.

[46] Mansfield E (1977). The Production and Application of New Industrial Technology.

[47] Scherer FM (1982). Inter-Industry Technology Flows and Productivity Growth. Rev Econ Stats.

[48] Griliches Z (1992). The search for R&D spillovers. Scand J Econ.

[49] Hall BH, Smith BR, Barfield CE (1996). Private and Social Returns to Research and Development in Technology R&D and the Economy.

[50] Jones CI, Williams JC (1998). Measuring the social return to R$D. Quart J Econ.

[51] Ruttan VW (2001). Technology, Growth and Development: An Induced Innovation Perspective.

[52] Steil B, Victor DG, Nelson R (2002). Technological Innovation and Economic Performance.

[53] Lawlor MS (2003).

[54] Bush V (1945). Science the Endless Frontier: A Report to the President By the Director of the Office of Scientific Research and Development.

[55] Cancer Research UK full Annual Review 2007/2008 http://www.cancerresearchuk.org/aboutus/whoweare/ourreportsandaccounts/annual_review_2007_08/.

[56] European Economic and Social Committee (2004). Building our common future; Policy changes and budgetary means of the enlarged Union 2007–2013.

[57] Eckhouse S, Sullivan R (2006). A survey of public funding of cancer research in the European Union. PloS Med.

[58] Presentation Dr Octavi Quintana Trias (Director, DG Research) (2006). Biotechnology for Health Future Perspectives. EC-US Task Force for Biotechnology.

[59] Commission of the European Communities (2009). Communication from the Commission to the European Parliament, the Council, The European Economic and Social Committee and the Committee of the Regions on Action Against Cancer: European Partnership.

[60] Wright K (2000). Charitable Change: creating a new culture of giving for Britain. LSE Magazine.

[61] Humphries N (1997). Varieties of altruism and the common ground between them. Soc Res.

[62] Wagstaff A (2005). On the road to a single European cancer society. Cancer World.

[63] Jonsson B, Wilking N (2007). Annals Oncol.

[64] CMR International (2005–2006).

[65] Kola I, Landis J (2004). Can the pharmaceutical industry reduce attrition rates?. Nature Rev, Drug Discov.

[66] Howe T (2003).

[67] Booth B, Zemmel R (2004). Prospects for productivity. Nature Reviews, Drug Discovery.

[68] Tolchin E (2004). Pharmaceutical giants fund-start-ups. Drug Discov Devel.

[69] MIT press release (2002). Novartis opens drug research center in MIT’s Tech Square. http://web.mit.edu/newsoffice/2002/novartis.html.

[70] Smaglik P (2002). Boston: a Magnetic hub. Naturejobs.

[71] OECD (2004). Science and Innovation Policy: Key challenges and Opportunities.

# Sub article: 4. Research Activity in Drug Development: A Bibliometric Analysis

**Published:** 2010-02-03

## 4.1. Background and Objectives

Traditionally, policy analysis of cancer drug discovery and development have relied on qualitative interview-based methodologies and quantitative data gathered from sales. The use of bibliometrics is a novel way to gather objective intelligence on research domains and the funding of these domains [1].

Research into the causes, prevention, diagnosis and treatment of cancer is a US$ 17 billion global enterprise, encompassing basic research on genetics and cell science, epidemiology, diagnostic tools and procedures, as well as the three main treatment paths of surgery, chemotherapy and radiotherapy. Each year, about 40,000 papers relevant to cancer research are published in scientific journals, providing a rich source of intelligence on who, what and where particular domains and types of research in cancer are being conducted [2]. Publications of research findings can be used as a proxy indicator, enabling examination of geographical distribution of cancer research activity, its characteristics (which manifestations of cancer? which approaches to tackling the disease? patient- or laboratory-based?), and whether these correlate with the burden of disease. Transnational comparisons may reveal that particular countries are under-researching cancer or certain aspects of the disease.

The funding of research is not always top-down, as driven by clear policy from government in response to a perceived need, but may also be bottom-up, especially where research projects are proposed by investigators and then selected for funding after a peer-review process. The motivation here may be intellectual, or it can be the personal experience of individual researchers whose family members or friends may have succumbed to a particular manifestation of the disease. Current methodologies to estimate research expenditure do not, as a rule take into account the complexity of funding sources and so given figures are often nothing more than guess-estimates. The use of bibliometrics provides an objective method for describing the funders of cancer research. That cancer research covers such a huge range of scientific endeavour, from the social to natural sciences, makes the task of developing realistic measures of the state and funding of global cancer research complex [3]. In this chapter, we provide the first application of bibliometrics to the study of cancer drug development.

The broad objectives of this chapter are as follows:

1. To study the trends in global cancer drug discovery and development, including a focus on 19 specific anti-cancer drugs chosen as a surrogate subset to cover specific time periods, types of anti-cancer drugs (new chemical entities and biologcals) and sources.
2. To understand the relationship(s) between research activity and site-specific Disability Adjusted Life Years (DALYs) are specific areas being under (or over) represented?
3. To review the trends in drug development research in terms of their research level, geography (who, where) and funding mix.

With regards to the first objective, apart from early phase clinical trials where we know publications (outputs) do not reflect activity, bibliometrics provides an objective window on the current and past state of cancer drug development. With access to the major databases we are able, using algorithms developed with key drug names, to analyse the trends in output and impact by country, institution and even researcher. We can look at how models of partnership (institution-to-institution and institution-industry) have changed and also at the patterns of funders over time. Such changes can then be reviewed in the context of the impact of national policies. This objective will focus on those cancer drugs with an MA and depending on complexity may also sample a cross-section of those NME’s that failed to make it to market.

With regards to the second and third objectives, the question of how and who funds cancer drug development can most effectively be answered by looking at the cumulative lifetime from incept (NME) to clinic. We know that this R&D lifetime is a complex interplay between many different countries, funders and people. Taking a representative sample of current cancer drugs, we will look at their funding histories from inception to market.

## 4.2. Methods

### 4.2.1. Focus of study

This study focused on a set of anti-cancer drugs composed of 19 new molecular entities, together with trigraph codes used in this chapter to designate them in the tables and figures (Table 4.1). These drugs were chosen, using statistical methods, as a representative group for drug development as a whole to cover new chemical entities (including endocrine therapies) and biologicals as well as to cover three distinct periods of cancer drug research.

The main information gathered was:

1. the numbers of papers from 1963 to 2009 for each of the 19 drugs published in the Web of Science (WoS);
2. their research levels (on a scale from clinical to basic);
3. their geographical origins (USA, Europe, Rest of the World);
4. the cancer site(s), for example breast, lung, for which the drug was being investigated;
5. the funding sources for the papers.

Data were also obtained on the total numbers of cancer research papers from 1984–2008 and on the numbers of such papers concerned with named drugs from a list of 150 (including pre-clinical designations) (Table 4.2).

### 4.2.2. Selection of papers

Articles, notes until 1996 and reviews were identified from the WoS from 1963 to mid-2009, using search statements looking for the selected drugs (Table 4.1) appearing in the paper title. Bibliographic details (authors, title, document type, language, source, addresses) of all such papers were downloaded to an MS Excel spreadsheet.

Subsequently, titles were filtered to allow all papers involving each of the 19 drugs to be marked with a ‘1’ in a separate column of the spreadsheet. The numbers of papers ranged from 10,299 for cisplatin (CIS) to 83 for lapitinib (LAP); early years (from 1963) contained only papers concerning vinblastine (VBL) and vincristine (VCR), whereas papers on sunitinib (SUN) appeared only from 2003. For drugs with longer histories, the papers were divided into five quintiles based on publication years, but the quintile sizes varied covering different numbers of years^w^.

Next, papers were examined for funding acknowledgements. The intention was to select reasonable size samples permitting a valid analysis for each drug and to uniformly cover the time frame in which papers appeared. Due to large variations in paper numbers per drug, more than two orders of magnitude, it was decided to make the sample sizes proportional to the cube roots of the paper numbers, then to make a selection of an equal number of randomly chosen papers per year if available (although in early years, very often all the papers were needed for the sample). The sample sizes varied from 360 for CIS to 72 for LAP, and the number of papers per year varied from 21 for bevacuzimab (BEV) to 4 for exemestane (EXE) and VBL.

### 4.2.3. Comparison with outputs of all cancer research and all drugs

The WoS was searched separately to determine the numbers of cancer research papers each year from 1994–2008 (15 years) by means of a filter, labelled ONCOL, consisting of two parts: a list of specialist cancer journals (e.g. British Journal of Cancer, Cancer, Leukemia) and a list of cancer title words (e.g., adenoma^x^, BRCA1, carcino^*^, daunorubicin, EBV), and papers were selected if they were in one of the specialist journals, or contained one of the list of title words, or both. The filter was developed by Dr. Lynne Davies of Cancer Research UK, and recently updated to include new drugs used only for cancer as well as genes pre-disposing to cancer. Its specificity (precision) and sensitivity (recall) were both close to 0.95. A subset of these papers was also identified having as a title word one of a list of 150 drugs used for cancer treatment (Table 4.2) – some are also used for other indications. There were 46,796 papers and analysed by year, by country (leading 15 countries whose papers were in the original set, Table 4.3) and also by cancer manifestation (site), leading 16 listed by WHO in its burden of disease statistics (Table 4.4).

Cancer represents over 16% of the estimated overall disease burden measured in DALYs in the 12 developed industrialised countries (Table 4.3) (excluding China, India and South Korea), but accounts for 5% of the world disease burden (Table 4.4). Lung cancer has the highest cancer burden in both DALYs and deaths, followed at some distance by stomach, liver, colorectal and breast cancer, although the latter receives much more publicity than the others [4].

The 15 leading countries vary in degree of overall cancer burden (Table 4.3). The proportion of world mean values for each country is cross-matrixed (Table 4.3, DALY %) with the proportion of each cancer site (Table 4.4, % DALYs) (Table 4.5). Particularly high values are tinted pink and grey whilst or particularly low values are tinted bright or light green. Some cancer types are relatively uncommon in high-income countries, such as cervical, leukaemia, liver, mouth/head and neck, oesophageal and stomach cancer (except in the Far East); others are more common such as bladder, colorectal, melanoma (especially in Australia), pancreatic, prostate and uterine cancer. Breast and lung cancer are a major burden everywhere, lung cancer especially in North America and Greece, although breast cancer is low in Korea and China.

### 4.2.4. Research levels

For each year, the ratio of papers outputs of the major 19 selected drugs was compared with total oncology research and total cancer ***drug-related*** papers. The latter tally was broken down by country and by cancer site to provide information on how cancer drug research overall is conducted. This was determined in two ways: by analysis of the actual titles of the papers, and by an analysis of the titles of all the papers published in the same journal for the relevant period. The procedure has been described earlier [2], based on the presence of more than 100 ‘clinical’ and more than 100 ‘basic’ words in the titles of papers. Examples include clinical words (abdominal, breast, child, depression, elderly) and basic words (apoptosis, binding, channel, DNA, embryos, folding, gene).

Groups of papers were allocated a mean research level on a scale from 1 = clinical observation to 4 = basic research, both on the basis of their titles (RL p) and their journals (RL j). Comparison of these two mean values showed whether the papers were published in more clinical or more basic journals. The determination of values of RL p and RL j for the different quintiles for each drug also allowed time variations to be seen. (For the ensemble of papers, the tendency was for them to become more clinical, but this was not so for all drugs.)

An additional indicator of research type was information on whether the paper was a report of a clinical trial, and if so, whether Phase I, Phase II or Phase III. This was obtained from the paper titles. Although most used Roman numerals, a few used Arabic numerals instead to indicate the phase number; also some papers described Phase I/II or II/III trials and thus allocated to both groups.

### 4.2.5. Geographical analysis

A special macro allowed for an analysis of the addresses on the papers and for each country’s contribution to be determined on a fractional count basis^y^. For some of the analysis, countries were divided into three groups: the USA, Europe (consisting of the EU27 Member States, plus Iceland, Norway, Switzerland) (EUR30), and the Rest of the World (RoW) dominated mainly by Japan, but also included Canada, South Korea, China, Australia and India. The contribution of the latter group normally increased over the five quintiles, but not always. For each of the 19 selected drugs, the geographical percentage distribution of papers in the five quintiles was determined, although for drugs with rather few papers these values often moved erratically.

In addition, the contribution of each of the 15 selected countries was determined for the whole set of papers and also for the individual drugs. International collaboration between the selected countries was calculated and presented as each country’s preference (or lack of it) for each other country, based on that country’s percentage presence in the set of papers.

### 4.2.6. Cancer manifestations and disease burden

The papers for the 19 selected cancer drugs was filtered to identify those papers concerned with one (or more) of the main 16 cancer sites (Table 4.4). For each cancer site, a set of title words and journal name strings was developed by Professor Sullivan, and applied to the file by means of another special macro written by Philip Roe. This listed in a single column the manifestation(s) investigated in each paper; about half the papers did not mention any of them. Each of these sub-filters was also prepared in the format used in the WoS and used to determine how many of all the drug-related cancer papers were directed to each cancer site, year by year. This allowed time trends to be seen after normalisation for the overall numbers of cancer research papers.

Comparisons were also made between the amount of research effort on all cancer drugs, on the 19 selected cancer drugs, and on the worldwide burden of disease of the 16 cancer sites. Some cancer sites appeared under-researched in relation to their burden; however, this may mean that other treatments were used (surgery and radiotherapy) including adjuvant.

### 4.2.7. Funding of cancer drug research

Funding of papers for each 19 drugs was also determined for a sample of papers. The methodology for this process has been established for many years and found that the number of such financial acknowledgements plays a major role in determining whether the papers will be published in high impact journals, in turn receiving many citations [5,6].

Acknowledgements were recorded as three codes: first, a ***trigraph*** denoting the individual funding body (e.g. MRC = UK Medical Research Council; CUK = Cancer Research UK); second, a ***digraph*** denoting the category (government, private-non-profit, industry, international and sub-categories of each of these) and third, the ***ISO digraph*** denoting the country (EU for the European Union, XN for international bodies). Previous funding analyses to date have been for papers from individual countries with distinctions made between country funding from government or charities and that from abroad. In this study, the focus was on individual drugs and their development over time so this distinction was dropped, and the analysis focussed simply on whether the funding sources were governmental, private-non-profit, commercial or international – or none. The latter is not uncommon in medical research, particularly for clinical work; in Europe such papers would normally be funded indirectly by the state either through the higher education or the hospital system. We also investigated the extent to which pharmaceutical and biotech companies other than that associated with the initial marketing of a drug were involved in supporting both early and later research.

In addition to recording all funding for a sample of the papers, addresses of all papers were searched for the presence of the company responsible for first marketing of each of the 19 drugs using company names, trigraph codes and search strings (Table 4.6). Twenty-six pharmaceutical manufacturers were identified in this way, 12 of which were responsible for developing the 19 drugs under investigation. A sub-set of papers from the year of marketing approval and previous years for each drug was separately analysed to test the hypothesis that the company developing the drug would be the only one supporting such research intra-murally during that time.

## 4.3. Results

### 4.3.1. Volume of cancer drug paper outputs

The number of papers for the 19 selected drugs increased rapidly over time, both because of the general rise in drug-related research and because new drugs have been successively added to the portfolio (Figure 4.1). The proportion of the 19 drugs in total cancer research and has remained relatively stable with a slight upward trend (Figure 4.2).

Papers on the 19 selected drugs increased from an average of 2.7% of all oncology in 1994–1998 to 3.6% in 2004–2008, whereas overall drug papers have only increased from 6.9% to 7.8% over the same period. The numbers of papers per 19 drugs are presented on a logarithmic scale (some double counting occurred: the sum of individual totals is 30,635, 5.4% more than the total number of papers in the file) (Figure 4.3).

The overall distribution of papers for the 15 nations, per integer and fractional count bases, found integer counts exceeding fractional counts due to international collaboration – particularly high for Australia and Sweden, but rather low for Japan, Korea, Greece and India (Figure 4.4). Language of the papers is predominantly English, with major European languages decreasing over time (Table 4.7).

### 4.3.2. National involvement in cancer drug research

#### [I] International collaboration

It is possible to determine how much the different countries value research participation in relation to their international collaboration in total, best calculated on a fractional count basis (Table 4.8). This is not symmetrical on a fractional count basis, although it would be if calculated on an integer count basis.

For example, Canada contributed 104 papers to the US integer count of 10,430, whereas the US contributed 163 papers to the Canadian integer count of 1090, with 309 papers with both Canadian and US authors. Since Canada’s fractional count contribution to the total paper set was 2.77%, or 4.13% if the US presence accounting for 32.9% was neglected, thus contributing 0.0413 x the foreign contribution to US papers (10430 – 9452 = 978), or 40 papers. Its actual contribution was almost 2.6 times this estimate, showing that Canadian scientists are very much preferred international partners for US researchers, statistically highly significant. The situation for Japan is the reverse, with only 86 contributions to US output compared with an expectation, on the basis of its percentage presence in the world less the USA, of 154 papers. On the other hand, Chinese scientists are somewhat over-selected (by x 1.2) by US researchers, showing that its policy of openness has achieved results.

The matrix of values of observed to expected contributions shows that international collaboration is still firmly based on geographical proximity, linguistic and cultural ties, though intra-European collaboration is of major significance (Table 4.9). Thus Canada and the USA favour each other, although the USA also has good links with Japanese and Indian scientists. Three far Eastern countries (China, Japan and Korea) all give above-average preference to each other, and Korea (but not others) to India. Within Europe, the Netherlands and the United Kingdom appear to play important roles in collaboration, and there are strong reciprocal links between the United Kingdom and Australia. Perhaps surprisingly, India prefers Germany to the United Kingdom among European countries (this has been observed in other fields), but its preferred partner is Australia.

#### [II] Countries involvement in research for 19 selected drugs

Table 4.10 shows that there is a big variation in the relative emphasis the different countries place on research in each of the 19 drugs. This pattern is seen more readily if the values of observed expected paper counts are calculated. For example, the expected UK output of papers on alemtuzumab (ALE) is 447 x the UK overall fractional percentage presence (7.06%) = 31.6, whereas the actual output was 143, showing a relative concentration of 143/31.6 = x 4.53. Naturally, there are also drugs where the relative concentration of the United Kingdom is less than unity. Table 4.11 shows the values, with the cells tinted to reveal the ones where the values depart from unity, up- or downwards.

Almost all the 19 drugs have countries with a particular interest in them, highlighted in bright green, or for some in pale green, and conversely most of the countries have done more work than average on some selected drugs. We note that two of the three drugs developed by AstraZeneca (anastrozole and tamoxifen) and the one developed by SmithKline Beecham (lapatinib) all have a strong UK presence; the one developed by Aventis (docetaxel) has a strong French presence and the one developed by Schering AG (temozolomide) has a strong German presence.

Data were also obtained on outputs of the 15 leading countries for all cancer drugs in the WoS in 1994–2008, presented as integer counts. Table 4.12 shows the comparison between their percentage presence in this dataset with the corresponding figures for the 19 selected anti-cancer drugs in the same years.

The differences in percentage presence are mostly very small, but the UK’s presence is higher here (since 4 of the 19 drugs were developed by UK companies) and China’s is lower.

### 4.3.3. Site-specific research in cancer drug research

Most of the 19 drugs have been tested for their utility in treating many different cancer manifestations, only four of them have been tested on eight or fewer manifestations. Nevertheless, it does appear that the drugs have been tested more in some cancers than others, shown by the relative application compared with norm values of each drug and manifestation (Table 4.13).

Data were obtained on research outputs of all cancer drugs against particular cancer manifestations (1984–2008), compared to research outputs for the 19 selected drugs (Table 4.14) and also to the relative burden for the 16 cancer manifestations in 15 leading countries collectively (Figure 4.5). (The relative burden has been weighted to take account of the relative numbers of papers on cancer drugs, so that the USA has the highest weighting and India the lowest. This allows comparisons between perceived overall burden and the cancer drug research portfolio.)

Research work on the 19 selected drugs clearly concentrates on gynaecological cancers (uterine, cervical, ovarian) and to a lesser extent on breast cancer; on the other hand, there is less work on leukaemia and lymphoma drugs. The comparison with the disease burden suggests that although for many sites the amount of research is about right, it is much too low for some relatively neglected cancers, such as lung, colorectal, oesophageal and pancreatic cancer. Only leukaemia seems to be somewhat over-researched, at least in comparison with its disease burden.

#### [I] National interest in cancer drug research related to site-specific cancer burden

Further analysis was carried out, again on a fractional count basis, to see if countries with bigger relative disease burden from particular cancer sites took this into account with their work on the selected 19 cancer drugs (Tables 4.15, 4.16).

Again, the observed number of papers can be compared with the expected number on the basis of each country’s overall output and the numbers of papers concerned with each cancer manifestation (Table 4.17). Ideally, the tinting of the cells in Table 4.17 should be just the opposite of that in Table 4.5, with countries with high cancer burden of a particular cancer site carrying out similar amounts of research. The correlation for individual countries should, in theory, be higher than for all types of cancer research, as the size of the national markets for drugs to combat these cancer manifestations are well known to pharmaceutical companies. In fact, the correlation is very poor for most of the 15 countries, except for China (r2 = 0.76), suggesting there is a national cancer drug research strategy in place. Australia is fair (r2 = 0.51), mainly because the relatively greatest output is in melanoma research (x 2.8) of which Australia has high incidences (x 5.0) (Figures 4.6 and 4.7)

### 4.3.4. Clinical versus basic cancer research types

For the analysis of research levels, the papers involving each of the 19 selected drugs were divided into five (and in four cases, only four) quintiles. Overall, the papers have become slightly more clinical over time, on both as a journal and an individual paper basis (Figure 4.8).

However, the situation with individual drugs varies greatly. First, there are big differences in the mean research levels of their papers (Figure 4.9): the vinblastine papers are the most basic and those on capecitabine and bevacizumab are the most clinical, with the difference being quite large. Second, the individual papers are mostly more clinical than the journals in which they are published. Third, for a few drugs the papers become more basic with time (anastrazole and tamoxifen based on paper titles, and cisplatin and vincristine on both scales). Fourth, for some drugs the second quintile is much more clinical than the first, but then the subsequent quintiles become more basic (Figure 4.10). There is a much bigger variation between the countries in terms of the mean research level of their papers (Figure 4.11).

India and China publish the most basic papers, probably because their clinical journals are not processed for the WoS, and Greece the most clinical ones. Indian and Chinese papers are, on average, more clinical than the others in the same journals, but for most other countries the reverse is true – particularly for those doing clinical work.

#### [I] Anti-cancer drug research activity according to clinical development phase

Some of the papers referred specifically to the work being part of a clinical trial, either Phase I, II or III (occasionally combinations). The percentage of each drug’s papers that formed part of a clinical trial ranged from below 3% (tamoxifen) to almost 40% (capecitabine) (Table 4.17).

Table 4.18 shows that Phase II trials dominated, with nearly two thirds of the total. This was also true for all the individual drugs except three, although the numbers of papers were not large for these (bortezomib, lapatinib and sunitinib). Perhaps surprisingly, Phase II trials usually came first, as early as or even earlier than Phase I trials, as shown in the example time pattern of papers for carboplatin (Figure 4.12). For this drug, the number of papers peaked around 1996, decreased, then rose again to a second, smaller peak in 2006. Clinical trial papers also showed two peaks, but this second peak (2006) was higher than the first.

Different countries showed different clinical trial outputs, negatively correlated with the mean research level of their papers (Figure 4.13). Greece produced relatively the most trials and India the least. Sweden produced fewer than expected based on the research level of its papers whilst Korea rather more, largely attributable to their choice of drugs on which they concentrated their efforts (Table 4.11) – Sweden on alemtuzumab and tamoxifen (fewer than 3% of trials papers) and Korea on capecitabine and irinotecan (30%) and gefitinib (15%).

### 4.3.5. Trends in global and regional cancer drug development

There was a gradual shift over time from the USA and the EUR30 countries to the RoW (Figure 4.14), with the latter group overtaking the USA and seems likely to overtake EUR30 shortly. Its recent rise is mainly due to increasing Chinese publication outputs, showing rapid increases lately after a temporary halt from 2001–2004 (Figure 4.15). This major expansion in scientific output parallels that seen in other areas of science but not yet on the scale seen in the physical sciences [7].

The pattern shown in Figure 4.14, with decreasing presence since the 1990s of both the USA and the EUR30 countries and an increasing presence of the RoW, does not prevail for all the drugs. First, there is a big variation in the US presence from almost 60% to barely 20% (Figure 4.16, Table 4.10), similarly in EUR30 countries and RoW. Second, for two drugs the US presence has increased over time (alemtuzumab from 10% to 44%; exemestane from 6% to 22%), and for six drugs the EUR30 presence has increased (bortezomib, lapatinib, sunitinib, trastuzumab, vinblastine and vincristine). The RoW presence has almost always grown, indicating a steady shift away from the USA and Europe.

Which countries in the RoW have made the largest contribution? Table 4.18 shows the percentage presence in six quinquennia for 10 RoW countries – the six listed in Table 4.3, plus the next four (Turkey = TR, Israel = IL, Taiwan = TW and Brazil = BR) and all others.

Although Korea, China, Turkey, Taiwan and Brazil have increased their outputs both absolutely and relatively, others have declined relatively from an earlier peak. This is greatest for Israel, whose output has even declined in absolute terms from 1995–1999.

### 4.3.6. Funding of cancer drug research

The analysis of the addresses for the presence of names of pharmaceutical companies (Table 4.6) yielded their paper outputs (Table 4.19). The 12 companies developing one or more of the 19 selected drugs were involved in a total of 1295 papers; in addition, the other 14 leading companies were involved in 337 papers. In total, 1589 papers had a pharmaceutical company address (5.5%); in addition, there were papers whose addresses included smaller pharmaceutical and biotech companies not listed in Table 4.6. Each paper was examined for each of the 19 individual drugs to see what part had been played by the drugs’ developers both before and after marketing approval (Table 4.20).

Overall, the number of papers involving companies other than the primary developer exceeds those involving the drug development company, but as expected, the latter dominates in the years leading up to marketing approval. This pattern holds true for all drugs except irinotecan, tamoxifen, trastuzumab and the two longestablished Eli Lilly drugs vinblastine and vincristine. These drugs all have more papers involving other companies than the development company as do several others such as anastrozole and cisplatin.

#### [I] Funding of cancer drug research by sector

The main analysis was in terms of the four main sectors: government, private-non-profit, industry and international. For the 19 selected cancer drugs, the breakdown of financial funding was analysed (Table 4.21). The sample sizes varied by about 6:1, but the complete set of papers for the different drugs varied by more than 100:1. The tinted cells in the five right-hand columns show there is a substantial variation in the amount of support from the different sectors for the 19 drugs. Bortezomib and vinblastine receive most from government (see also the blue bars in Figure 4.17) and exemestane and anastrozole the least; there is relatively less variation in the support from private-non-profit sources although temozolomide benefits most; exemestane, followed by capecitabine and anastrozole, have the most support from industry (red bars in Figure 4.17). There is very little international support for any of the drugs. Papers with no funding acknowledgements must be in practice funded, normally they would be supported by university or hospital funds, which in Europe would come from national or regional governments.

Variation in longitudinal support for drugs with sufficient papers was completed, six drugs had 130 or more papers with funding data and chosen for a funding analysis in five quintiles (Figure 4.18). There appears to be a modest reduction in the amount of support over time, both of government and industrial funding between the first and second quintiles, though the latter increases again subsequently to about 27%. Private-non-profit funding is fairly constant averaging 39% for these six drugs.

Variation in sectoral support for papers from different countries was examined on an integer count basis for the 15 leading countries (Table 4.22, Figure 4.19). Sample sizes were not modified to take account of the much smaller numbers of papers from lesser-producing countries, for example the sample of Chinese papers is only 20, and that of Indian papers only 11, whereas there are 761 US papers.

The number of internationally funded papers is too small for the analysis to be meaningful, but there are several interesting trends. The far eastern countries (China, Japan, Korea) rely mainly on government sources with very little private-non-profit sources, whilst both China and Korea have little industry support from industry. Of the European countries, Greece and Sweden have little government support; however, Sweden together with the United Kingdom enjoys high private-non-profit funding. Germany, France and the United Kingdom have the largest proportion of support from industry, noticeably more than the USA. Canada and the USA receive more government support than any of the European countries.

Finally, we examined the sectoral support for drugs intended for use in the 16 cancer sites (Table 4.23). Data for cervical and oesophageal cancer were omitted due to insufficient papers (7 and 5, respectively). The differences are relatively minor between the different sites, particularly when accounting for small paper sample size (13 of the 16 cancer sites were less than 100 papers). Leukaemia, followed by lymphoma, gets most support from government; the situation with respect to private-non-profit funders is similar. Industry relatively favours research on the application of drugs to breast and colorectal cancer.

#### [II] Leading funders of cancer drug research

The 1953 papers whose funding had been recorded acknowledged a total of 2833 funding sources. These were tallied in order to list the leading sources of support for cancer drug research, under the three sectoral categories (Tables 4.24 and 4.25). The individual institutes of the US National Institutes of Health have been grouped together, as some authors acknowledge the institute, and some just the NIH. Similarly, acknowledgements to subsidiary companies have been grouped under the main parent company, even if it was not the owner at the time of the research, with the sole exception of Genentech, Inc., Table 4.24 shows the dominant position of the US National Institutes of Health/National Cancer Institute in funding cancer drug research, with support to over one third of the US papers.

## 4.4. Conclusions and Policy Issues

This chapter examined research papers on 19 selected cancer drugs (articles, notes and reviews) from the WoS for 1963 to mid-2009. The papers were identified via the drug names in their titles, including trade and code forms. A total of 28,752 papers were included in the analysis, after removal of ones that had been identified in error and almost all were in English.

Papers on the 19 selected drugs rose from 200 annually (1980) to 900 (1995) to more than 2000 annually by 2007–2008. The latter figure represents about 4% of all cancer research output and half of the research paper output of all 150 cancer drugs. The number of papers on the individual drugs varied from 83 for lapatinib, first marketed in 2007, to 10,299 for cisplatin from 1978. The leading countries contributing to the research were the USA (33% of fractional count total), Japan (10.6%), Italy (7.5%) and the United Kingdom (7.1%).

International collaboration has increased but is still firmly based on geographical, historical and linguistic links between nations. Thus, the USA and Canada each preferentially select co-authors in the other country, as well as between EU Member States, especially the Netherlands and the United Kingdom. A matrix of the ratios of observed to expected papers from leading 15 countries for 19 selected drugs showed most drugs having one or two countries with concentrated output yet conversely with less work on other drugs. The 19 drugs were compared against 16 leading cancer manifestations, finding some cancer sites favoured over others (e.g. tamoxifen for breast and uterine cancer, cetuximab for colorectal cancer). However, the research portfolios for these 19 drugs in 15 countries correlated very poorly with their national cancer burdens of the 16 cancer manifestations, except for China and to a lesser extent Australia.

Over time, the research levels of the papers (on a scale from clinical = 1 to basic = 4) became slightly more clinical, but not for all the 19 selected drugs (exceptions were cisplatin, vincristine, anastrazole, tamoxifen). Work on vinblastine and bortezomib was the most basic, whilst bevacizumab and capecitabine the most clinical. Papers from India and China were the most basic likely, as their national clinical journals are not covered in the WoS, and those from Spain and Greece the most clinical. About 15% of the papers described phased clinical trials, mostly Phase II. Greece produced most of such trials (34%) and India the least (2%), and negatively correlated with the research level of the countries’ papers.

Over the period of analysis, the geographical balance shifted from the USA and Europe to the RoW, particularly China, which accounted for almost 5% of global output by 2008. The US presence varied between 57% for lapatinib to 20% for exemestane and increased over time for alemtuzumab and exemestane.

In the papers’ addresses, the presence of 26 leading pharma companies including the 12 associated with development of the 19 selected drugs, occurred for 1589 papers (5.5%). Leaders were Aventis (274 papers), AstraZeneca (173) and BristolMyersSquibb (155). In the years up to and including when initial marketing approval was given, the company developing the drug dominated the output for 14 of the 19 drugs, as expected.

### 4.4.1. Policy conclusions

Modern cancer drug discovery and development ‘starts’ in the early 1970s, follows a slow trajectory, and then activity increases substantially between 1990 to 2000. From 2000 onwards, the trajectory of outputs increases dramatically, whilst the overall output of cancer drug development papers globally remains constant at just under 10%. This is entirely the result of a concomitant major increase in world cancer research activity. We found that the rate of drug development activity has substantially increased, with more publications per time period for newer agents than older ones. However, from cumulative data, we show the development of cancer drugs does not stop, even for those with more than 20 years of marketing authorisation (e.g. Cisplatin 1978, Tamxoifen 1986). The main geographic locations are the USA, Japan and Europe (primarily Italy, the United Kingdom, Germany, France) with over 98% of publications in the English language.

Trend analysis shows collaborations at the national level have remained very stable since the 1990s. International collaboration is still firmly based on geography, linguistic and cultural ties, although intra-European collaboration has grown since early 2000. Thus Canada and the USA favour each other, although the USA also has good links with Japanese and Indian scientists. The three far Eastern countries (China, Japan and Korea) all give above-average preference to each other, and Korea (but not the others) to India. Within Europe, the Netherlands and the United Kingdom appear to play important roles in collaboration, and there are strong links between the United Kingdom and Australia. Perhaps surprisingly, India prefers Germany to the United Kingdom among European countries (this has been observed in other fields as well), but its relatively preferred partner is Australia. Whilst cultural transmission of science between geographically or linguistically close countries is to be expected from previous research, a question mark has always remained about whether outreach/international policies set at the national (or supra-national) level have any affect on the direction of collaborations and cooperation. Our data on intra-European and USA-China collaboration support the premise that topdown ‘iron triangles’ can promote cooperation [8]. In the former case, the temporal concordance between the evolution of the European Research Area and the increase in intra-European cooperation in cancer drug development is strongly suggestive [9]; however, the lag time in seeing the benefits of such policies, at least in this area, is around a decade.

Apart from the Japanese focus on EGFR inhibitors, we found no specific trends or associations between geographic regions and development life cycle of specifc NMEs. It appears one or two countries appear take a ‘lead’ in research around a specific cancer drug. Up to mid-1990s, there was a strong association between location of pharmaceutical companies and research activity; however, this association has loosened with increasing numbers of NME being developed in a distributed manner. We also found, in absolute terms and contrary to ‘negitive views’ of Europe’s weakness compared to the USA [10], Europe and the USA were equal ‘intellectual’ partners in cancer drug research outputs.

In policy terms, discussion of a balanced portfolio has tended to focus either on relative investments (and activity) in specific cancer research domains (e.g. prevention, fundamental biology) or in the balance between effort allocations to different disease areas by pharma companies [11]. Relatively little has been asked about the balance in site-specific later stage cancer drug development. Using this bibliometric approach we have found an objective way to quantify the relative focus and lacunae in cancer drug development. The comparison of disease burden suggests that although for many sites the amount of research is as expected, it is too much low for some cancers such as lung, colorectal, oesophageal and pancreatic. Only leukaemia seems to be somewhat over-produced compared to its disease burden – perhaps as a result of using leukaemia as a ‘model system’ for testing many NMEs.

At the country level, we found little correlation between the burden of site-specific cancer and associated site-specific cancer drug development, confirming bottom-up market forces are the predominate drivers in most countries. Interesting exceptions are China, and less so Australia, with clear, long-term correlations between disease burden and site-specific drug development. China in particular has long pursued a central cancer drug development policy in line with its other S&T strategies [12]; however, overall, there is no evidence that either approach leads globally to better or worse outcomes in terms of site-specific development. The key policy message is that for some cancers a site-specific focus may be required.

A widely held assumption is that cancer drug discovery and development progresses through set phases of pre-clinical, mostly basic laboratory, research to eventually clinical research. Whilst this view may hold true for the absolute extremes of development, our results show cancer drug development progression mixes both basic and clinical research over time. There is no simple hand off from one type to the other at any point. Individual countries also produce either predominantly basic or clinical cancer drug development research. Thus, cancer drug development policies should cover the full spectrum research, both clinical and basic.

Cancer drug development between the 1970s and 1990s had some linearity, yet the emergence of ‘translational’ research has driven increasingly complex, multi-party collaborations and parallel research at the class as well as individual drug level [13]. Most clinical development occurs at the Phase II stage; however, the ‘clinical development’ phase continues throughout a drug’s lifespan from pre-MA onwards. Indeed, our data clearly show that individual cancer drug development never stops either clinically or pre-clinically. Unsurprisingly, Phase III (large-scale clinical trials) only make up a very small proportion of overall research activity, which has important policy implications. Cancer drugs are continually ‘in development’ against new indications in addition to being refined with new schedules, regimens, etc. The old paradigm of cancer drug development ending with its marketing authorisation is no longer valid in any way, supported by continued publication production post-marketing.

Our data support current perception that cancer drug discovery and development is entering a global phase. Outputs from the RoW (in particular China) are set to overtake those from Europe and the USA, giving some indication of the competitive nature of this area of drug development and huge opportunities for major breakthroughs. It will be essential to develop new policies containing collaboration and cooperation to bridge geographic and sociocultural gaps between investigators in different countries and regions. This is no easy task as numerous hurdles block sustainable cooperation [14]. At individual drug level, all three regions contribute in different capacities to overall output but with large variations (e.g. USA 20%–60%) and increasing RoW share as the only clear trend.

The current policy paradigm around cancer drug discovery and development views research activity progression from an exclusively private (industry)-based activity to eventually a mixed economy with both private and public investment in the post-marketing phase [15]. Our findings indicate a much more complex picture with important policy implications. Drug-by-drug basis, large variations in government, philanthropic and industry sectors support were found to contribute to overall drug development. Rationales for this variability accounting for primarily one funding source or another are often based on a drug’s life history (e.g. temozolomide primarily produced by academic). What is clear is that all three sectors are equally important in contributing to drug development. Furthermore, even the earliest development period have funding contributions eventually brought into public domain. This is an important point, as industry contribution is underestimated due to unpublished research activities. Our data clearly shows that public-private partnerships, even in early stages, is of major importance to the overall development life cycle. Federal and philanthropic funders making up public sector support are a mixture of endowed and collecting charities, as well as ministries and arms-length funding bodies. In the pre-marketing phase, development is primarily by one company, but post-marketing most of the publications cite multiple private funders, which indicate a multi-sourced flow of private capital into further development. Our data indicate public-private partnerships are now the normative model from early pre-clinical to clinical development and beyond into Phase IV.

## Figures and Tables

**Figure 4.1:** Total WoS output for 19 cancer drug research papers (3-year running means) (1970–2007)

**Figure 4.2:** Proportion of total drug (blue) and 19 drugs (red) cancer research papers of total cancer research papers

**Figure 4.3:** WoS cancer drug papers for 19 cancer drugs (1963–2009)

**Figure 4.4:** Distribution of papers in 15 countries (integer, fractional counts)

**Figure 4.5:** Cancer drug paper outputs (1994–2008) versus cancer burden of disease (% DALYs) (2004) (weighted by the countries’ presence in cancer drug research)

**Figure 4.6:** Country-specific correlation coefficient of cancer drug paper outputs versus burden for 16 cancer sites

**Figure 4.7:** China: cancer drug research output versus burden from 16 cancer sites

**Figure 4.8:** Mean research level (RL) of all cancer drug papers in five quintiles

**Figure 4.9:** Mean research level (RL): journal source (RLj) versus title source (RLp) in 19 cancer drug papers (1963–2009)

**Figure 4.10:** Average research level (RL) per time quintile for 6 cancer drugs

**Figure 4.11:** Mean research level (RL) from 15 countries in 19 cancer drug research papers per paper title (paper) and per journal source (journal)

**Figure 4.12:** Phased carboplatin clinical trials longitudinal paper outputs (3-year running means)

**Figure 4.13:** Percentage cancer drug clinical trials output per mean research level in 15 countries

**Figure 4.14:** Geographical distribution of cancer drug papers in three world regions (USA, EUR 30, RoW) (quintiles, fractional counts) (1963–2009)

**Figure 4.15:** Chinese cancer drug papers, 3-year running means (fractional counts) (1996–2008)

**Figure 4.16:** Distribution of 19 cancer drug papers by geographical region: USA, EUR30 and RoW (fractional counts)

**Figure 4.17:** Funding sources in 19 cancer drug papers (1963–2009)

**Figure 4.18:** Funding sources for 6 out of 19 cancer drugs^*^ in different time quintiles ^*^Six drugs = carboplatin, cisplatin, docetaxel, irinotecan, tamoxifen and vincristine.

**Figure 4.19:** Funding sources for cancer drug papers in 15 leading countries (1963–2009)

**Table 4.1:** Major 19 selected cancer drugs used for data collection

*Source:* The authors.

**Table 4.2:** List of all drugs listed as being approved for cancer treatment (2009)

***Source:*** The authors.

**Table 4.3:** Estimated total disease burden (DALYs) and cancer burden (DALYs, %) in 15 countries (2004)

***Source:*** The authors

**Table 4.4:** Main 16 cancer sites, disease burdens (DALYs) and mortality rates (2004)

***Source:*** See [4] in Chapter 1.

**Table 4.5:** Ratios of relative disease burden (DALYs) for 16 cancer sites to global average in 15 countries

***Notes:*** **Values over 2.0 coloured pink; values over 1.41 in light grey; values below 0.71 in light green; values below 0.5 in bright green.**

**Table 4.6:** Pharmaceutical companies involved in 19 cancer drugs: trigraph identifying codes, search strings and drug codes

**Table 4.7:** Publication languages in cancer drug research papers (1963–2009)

**Table 4.8:** Matrix of total cancer research papers per country to the 19 selected cancer drug papers per country (fractional counts)

**Table 4.9:** Matrix of ratios of observed to expected cancer drug research papers per country

**Table 4.10:** Cancer drug paper ouputs in 15 countries for 19 selected drugs (fractional counts)

**Table 4.11:** Relative research concentration of the 15 countries for 19 selected drugs (1963–2009)

**Table 4.12:** Global cancer drug research in 15 countries for all drugs (ALL) and 19 selected cancer drugs (19D) (%, integer counts) (1994–2008)

**Table 4.13:** Research paper outputs: 19 cancer drugs per top 16 cancer sites (1963–2009)

**Table 4.14:** Cancer drug research outputs in 16 cancer sites for all drugs (ALL) and 19 selected drugs (19 D) (1984–2008)

**Table 4.15:** Numbers of cancer papers (fractional counts) for the 15 leading countries for 19 selected drugs in 16 cancer sites

**Table 4.16:** Ratios of observed to expected cancer drug research paper outputs for 15 countries for 16 cancer sites

**Table 4.17:** Phased clinical trials paper outputs in 19 cancer drugs (1963–2009)

**Table 4.18:** Global percentage of 10 RoW countries in 19 cancer drug papers (fractional counts) (1980–2009)

**Table 4.19:** Intramural papers in 19 cancer drugs per phama company (1963–2009)

**Table 4.20:** Pre- and post-marketing approval paper output in 19 cancer drugs by their developing pharmaceutical company versus other companies

**Table 4.21:** Funding sources in 19 selected cancer drug papers (1963–2009)

**Table 4.22:** Funding sources for cancer drug papers in 15 countries: Ratio of percent of national papers to world mean (1963–2009)

**Table. 4.23:** Funding sources for cancer drug research papers in 16 cancer manifestations (1963–2009)

**Table 4.24:** Governmental organisations supporting cancer drug research (1963–2009)

**Table 4.25:** Non-profit organisations supporting cancer drug research (1963–2009)

## References

[1] Sullivan R (2008). Trends in the global funding and activity of cancer research. Molec Oncol.

[2] Lewison G, Paraje G (2004). The classification of biomedical journals by research level. Scientometrics.

[3] Lewison G (1996). The definition of biomedical research subfields with title keywords and application to the analysis of research outputs. Res Evaluation.

[4] Lewison G, Tootell S, Roe P, Sullivan R (2008). How do the media report cancer research? A study of the UK’s BBC website. Br J Cancer.

[5] Dawson G, Lucocq B, Cottrell R, Lewison G (1998). Mapping the Landscape: National Biomedical Research Outputs 1988–95.

[6] Lewison G (2003). The publication of cancer research papers in high impact journals. Aslib Proceedings.

[7] Shelton RD (2008). Relations between national research investment and publication output: application to an American paradox. Scientometrics.

[8] Overman S, Simonton D (1986). Iron triangles & issue network. Public Admin Rev.

[9] Jungbluth S (2007). Europe combating cancer: the European Union’s commitment to cancer research in the 6th Framework Programme. Mol Oncol.

[10] Aho Report (2006). Report of the Independent Expert Group on R&D and the Innovation Appointed Following the Hampton Court Summit and Chaired by Esko Aho, Creating an Innovative Europe.

[11] Karlberg JPE (2008). Trends in disease focus of drug development. Nature Rev Drug Disc.

[12] Cheng MH (2007). Cancer research funding in Asia. Molec Onc.

[13] Cambrosio A (2006). Mapping the emergence and development of translational cancer research. Eur J Cancer.

[14] Trivers RL (1971). The evolution of reciprocal altruism. Quart Rev Biol.

[15] Kaplan W, Laing R (2004). Priority Medicines for Europe and the World.

# Sub article: 5. Promoting and Supporting Cancer Drug R&D – Results of a Senior Scientists Survey

**Published:** 2010-02-03

## 5.1. Background and Objectives

The purpose of social and public policy in cancer drug development is to understand, evaluate, interpret and define policies that form priority areas for the future. The existing literature is replete with studies examining consumer, patient and industry perspectives on drug development policy, but a key often neglected stakeholder group are those clinicians and scientists carrying out front-line research into the next generation of anti-cancer treatments [1–3]. Background sources on public policy development in cancer and drug development per se, such as the Institute of Medicine (IOM) Forum and the Tufts Centre for the Study of Drug Development, intellectually underpin the objectives framework for this study of key opinion leader views on anti-cancer public policy.

The objective of this research was to elicit and semi-quantify the views of key clinical leaders in cancer drug development from Europe and the USA to:

- critically examine whether policies affect funding models and, if so, how;
- define the scope of public sector involvement in the process of discovery and research in cancer medicines;
- critically evaluate key environmental policies for successful cancer drug development;
- understand which policies are likely to have the greatest pay-back in providing the correct environment for R&D;
- identify the strengths and weaknesses of public-private partnership (PPP) models;
- critically examine these models in terms of the balance between policies affecting public and private sector;
- state the key policy areas for ‘success’ in anti-cancer drug development; and
- compare the relative importance of different policy areas across different domains.

## 5.2. Methodological Approach

In order to elicit the views of senior clinicians and scientists on policy issues around cancer drug development, we proceeded in three steps: first, we identified the relevant faculty; second, we developed a tool that would be used for this purpose and third, after validating it, we administered that tool to the identified faculty.

### 5.2.1. Semi-structured interviews

From October 2008 until March 2009, we carried out semi-structured interviews with 28 members of the faculty to discuss key policy areas. These interviews took place by phone or in person lasting 25 min to 1.5 h and addressed a number of key issues including a strengths, weaknesses, opportunities and threats (SWOT) analysis, new drug development models, regulatory environment and funding.

### 5.2.2. Faculty: inclusion criteria and demographics

The primary cancer drug development faculty was selected from senior clinical and non-clinical active researchers from across Europe and the USA. The inclusion criteria required they be active publishers in the last five years and regularly invited to speak at major conferences on cancer drug development (among others AACR, EORTC, NCI, AACR). The final list was reviewed for geographic, speciality and gender balance before finalisation. Questionnaires were sent to all faculty members (*n*=102) for whom verified contact details were present. In addition, the questionnaire was forwarded to an additional 16 faculty from the initial listing.

In order to ensure an accurate analysis of critical data, demographic information was elicited from the questionnaire including:

- age and gender;
- clinical/non-clinical and research domain (NCE / Biological)
- geographic location.

As the questionnaire was distributed as ‘open’, additional faculty were added if they fulfilled the criteria of geography and seniority. All responses were anonymous and faculty were provided with full details of the purpose of the questionnaire and its future use/distribution.

### 5.2.3. Questionnaire development

Between 4 December 2008 and 15 March 2009, 12 members of the selected faculty were interviewed on a one-to-one basis as part of the semi-structured interviews to build the questionnaire. This was then externally validated when in beta-version. In addition, a review of the background literature was undertaken including review of prior policy studies and public research funding organisations, this information was incorporated into the results where relevant.

Four key policy areas were identified:

1. Funding (particularly public sector) – Investment by national bodies (philanthropic and federal) as well as supra-national initiatives.
2. Environment for R&D – This included basic infrastructure (dedicated beds, laboratory space, bio-banking etc.) and intellectual environment (i.e. training and career development in early phase clinical trials and pre-clinical drug development).
3. Pubic-private interactions – What PPP models had the faculty experienced? Where the current deficiencies and how could this be rectified?
4. A variety of distinct areas that were considered essential for the ‘success’ of future cancer drug development (e.g. regulatory and drug reimbursement).

The results of the qualitative survey were discussed with members of the external advisory board and other key faculty members. Specific questions were chosen in each area and responses were built around a semi-quantitative Likert scale. A free-text response area was also provided, due to the need to broadly capture public policy views from this faculty.

The beta-version of the questionnaire was subsequently circulated to 10 randomly chosen faculty members and further evolved following feedback, including adding a demographic profile section onto the beginning of the questionnaire. The final version was checked for legibility and time-to-complete (15–20 min), and pre-letters were sent to all faculty members prior to its electronic distribution in July 2009, with a two-week deadline on completion. Follow-up notices were sent one week before deadline and additional reminder letters were sent two weeks after the deadline

### 5.2.4. Questionnaire analysis

Responses were entered into a standard excel spreadsheet and converted into a 10-point Likert scale. A Likert item is a statement in which the respondent is asked to evaluate according to any kind of subjective or objective criteria, generally, the respondent’s level of agreement or disagreement is measured. Usually five-ordered response levels are used, but in this case we used a 10-point scale to improve statistical analysis. Sub-group analysis according to demographic profiles was undertaken. In terms of the other data characteristics, there was very little difference among the scale formats with regards to variation around the mean, skewness or kurtosis.

Likert scales may be subject to distortion from several causes. Respondents may avoid using extreme response categories (central tendency bias); agree with statements as presented (acquiescence bias) or try to portray themselves or their organisation in a more favourable light (social desirability bias). We have attempted to avoid the acquiescence bias by providing a scale with equal numbers of positives and negatives. The anonymous nature of our approach also reduces the social desirability bias.

After the questionnaire was completed, each item was analysed separately, or in some cases item responses were summed to create a score for a group of items (summative scale). We considered individual Likert items as interval-level data due to the 10-point scale, equidistant adjacent pairing about a mid-category and visual analogue scale with equal spacing of response levels. Secondary analysis of data treated as ordinal was undertaken to check internal consistency.

When treated as ordinal data, Likert responses were collated into bar charts, central tendency summarised by median, mode and mean, dispersion summarised by the range across quartiles, as well as analysis using non-parametric tests (e.g. Chi-square test, Mann-Whitney test, Wilcoxon signed-rank test, Kruskal-Wallis test). Responses to several Likert questions in the questionnaire were summed when questions using the same Likert scale, defendable as an approximation to an interval scale and thus treated as interval data measuring a latent variable and subject to parametric analysis.

## 5.3. Results of the Clinician and Scientist Survey

### 5.3.1. Interviews with drug development faculty

Policy literature to date has focused almost exclusively on commercial drug development, whilst ignoring the roles of public funders and the academic community. In Europe, public funders of cancer drug development have undergone major environmental changes since the 2004 introduction of the European Clinical Trials Directive. Whilst funders and academic institutions have long conformed with the standards of ICH GCP, the new requirements for laboratory studies to be carried out to GCP (GCLP) in addition to continued issues surrounding acceptability of rodent-only toxicology, increased administration associated with Clinical Trials Authorisation process, plus requirements for all clinical trial supplies to be made to GMP standards in a licensed facility^z^ have led to perceived and real down-turns in investigator-driven cancer drug development.

In addition to the changes in regulatory and legal requirements, public funders and host institutions have also experienced major changes over the last five years in the following areas: PPP, contracts, timeliness and paediatric oncology.

#### [I] Public-private partnerships

There is not one model governing the interaction between public funders/host institutions and the myriad of pharmaceutical and biotechnology firms. In the latter, most of the faculty interviewed were clear that public funders/institutions retained substantial control over the relationship, including ownership of trial data, protocol design, trial completion and publication of results. With major pharmaceutical companies, however, the balance often tipped the other way to almost exclusively developed and run early phase clinical trials, which do not undergo the peer review mechanisms found in public funders/institutions. In some cases, the academic faculty had found the protocol development was conducted with their input, and then the trial was run in a third party country.

#### [II] Contracts

The unanimous view from interviews was a geometric progression in complexity and time-lines for arranging contracts between parties prior to beginning pre-clinical and clinical projects. This increased bureaucracy was not only confined to PPP, but also to public-public. On average, there are 6 to 11 agreements per project, all taking significant time and negotiation. Complexity increases substantially with advanced biologicals, in particular for gene therapies suffering from the ubiquitous ‘patent stacking’ problem.

#### [III] Timeliness

Across the board, it was expressed that time needed for public funders/host institutions to pursue cancer drug development in either pre-clinical or clinical phases, particularly the latter, had dramatically increased. A variety of reasons were given including contract negotiation (see above), lack of major centres with sufficient critical mass and infrastructure resources (specifically directed at larger Phase II studies), under-funding/low sourcing from public funders, and time needed to validate PK and primary/secondary PD processes.

Faculty were clear that public funders/host institutions had played a major role in cancer drug development over the last 20 years. In addition to the role of national bodies supporting pre-clinical and clinical development (e.g. UK, National Cancer Institute (NCI), Cancer Research UK (CRUK)), other organisations such as the Southern Europe New Drug Organisation (SENDO) have also been instrumental in supporting academic investigators. However, faculty also stressed this complex funding arrangements between public and commercial sources have always been around in one form or another. Whilst there might indeed be a higher attrition rate in publicly supported post-Phase I development, many of the NME’s were highly novel, providing supplemental proof-of-principle data benefiting other programmes; thus the knowledge derived from these studies was critical to future generations of drug development. Indeed, public funding was expressed as particularly important to many high-risk research areas, such as complex cell therapies, novel derived antibodies and gene therapeutics.

Faculty primarily involved with biologics research were different in this respect to their colleagues involved with NME as they considered public funding to have driven (and continuing to drive) the development of very novel biologicals. These faculty were clear this was a key area for public support, targeting truly risky innovative approaches rather than developing ‘me too’ or second-in-class medicines. Furthermore, their emphasis on proof-of-concept studies as a way to improve overall attrition rates and truly novel approaches has been supported by other studies [1]. Again, the importance of both funder and host institution support was made with notable examples of translation into major advances in cancer drug discovery (e.g. New Agents Committee of the Cancer Research Campaign, CRUK).

#### [IV] Paediatric oncology

The paediatric oncology drug development community had specific key issues. Whilst paediatric oncology outcomes have improved dramatically over the last 30 years, there remains large gaps for new medicines. The research community, through International Society of Paediatric Oncology (SIOPE) and Innovative Therapies for Children with Cancer (ITCC), has been active in promoting this area at both national and European levels for the last decade. Their work has clearly identified the major policy areas for drug development in paediatric oncology.

Each year in Europe, approximately 12,000 new childhood cases of cancer are diagnosed and approximately 3000 children succumb to cancer. Childhood cancers differ significantly from adult cancers in terms of histology and sensitivity to conventional treatments, explaining why the prognosis of children with cancer has dramatically improved over the last 40 years. Standard treatments have been developed by academic clinical research networks at the national, European and international levels (SIOP); however, involvement by the pharmaceutical industry has been limited. Despite improved paediatric disease-free survival rates (ca. 65% across all paediatric cancers), cancer is still a life-threatening disease in children and remains the major cause of death from disease beyond the age of 1 year. Cure is often only achieved at a substantial cost (major organ toxicity, developmental abnormalities, secondary tumours). These long-term soliloquy constitute significant health care burden, reducing both life expectancy and quality of life for childhood cancer survivors.

Access for children to innovative-targeted therapies is extremely limited in Europe, partially due to paediatric oncology not representing a large, and hence financially attractive, area for drug marketing. This limited commercial potential results in pharmaceutical companies often not undertaking drug development specific for targets only present in paediatric malignancies.

Failure to develop effective new treatments for childhood cancers is of major concern, given the consequences of delaying the clinical evaluation of a potentially active drug. As a consequence, years of socially and economically useful life are lost, both for the child and the parents, in addition to the tragic human costs to the individual child, family and friends.

In addition to the general lack of paediatric drug development in Europe, major disparities currently exist between European member states. In some countries, such as the UK and France, only a small number of experimental studies can be conducted because of major difficulties in obtaining new drugs for evaluation. In other countries, such as Germany, every child has the right to receive any drug that is marketed in adults, even if no safety or efficacy data are available to support its use in children. Furthermore, in many countries new drugs are not available at all for children, leading to desperate parents inappropriately transferring terminally ill children to foreign countries for treatment (particularly the USA).

The regulation on Orphan Medicinal Products, adopted in 1999, has significantly improved the level of drug development in rare diseases in Europe. On 14 December 2000, the Health Council adopted a resolution on paediatric medicinal products [5]. In addition, the EU Regulation on paediatric medicines entered into force in late January 2007 [6]. This Regulation aims to establish a legislative framework to fulfil the following main objectives:

- to increase availability of medicines specifically adapted and licensed for use in the paediatric population;
- to increase information available to the patient/carer and prescriber about the use of medicines in children, including clinical trial data and
- to increase high-quality research into medicines for children.

These will be achieved through a system of requirements and incentives. The main elements of the finalised Regulation include:

- the establishment of a new body, the Paediatric Committee, sited at the European Medicines Agency (EMEA);
- a requirement for new products and products currently covered by patent protection to include paediatric data based on a paediatric investigation plan (PIP), and a six-month extension of the supplementary protection certificate (SPC) if PIP information is incorporated into the Summary of Product Characteristics (SmPC);
- for orphan medicinal products, a two-year extension of market exclusivity if information arising from a completed PIP is incorporated into the SmPC;
- For off-patent products, a new category of marketing authorisation called the paediatric use marketing authorisation associated with 10-year data and market protection;
- an European database of paediatric clinical trials, partially to be publicly accessible;
- a requirement to submit paediatric clinical trial data to the regulatory authorities;
- coordination of an European Paediatric Clinical Trials Network;
- funding for the study of off-patent medicines provided through the European Community Framework Programmes (FP6, FP7) and
- an identifying symbol on the package of all products authorised for use in children.

The US Food and Drug Administration has implemented similar regulatory initiatives (***Paediatric Exclusivity and Paediatric Rule***, 1997), which have significantly increased the number of paediatric studies [7]. These European and international developments are expected to create a significant demand for pharmaceutical companies to increase the number of paediatric clinical trials with new anti-cancer drugs.

During the past 10 years, initiatives have been undertaken by paediatric oncologists in Europe to promote the clinical evaluation of new anti-cancer compounds in children within national academic paediatric oncology groups. In 1995, collaboration was established between the Pharmacology Group of the French Society of Paediatric Oncology (SFOP) and the New Agent Group of the UK Children’s Cancer and Leukaemia Group (CCLG), now representing 20 clinical paediatric oncology centres. Other European cooperative groups, such as those in the Netherlands, Italy and Germany, have recently joined this collaboration and initiated new drug trials [8].

In addition to clinical research, high-quality and internationally competitive basic research on the genetics and biology of paediatric malignancies is being performed in Europe. There is a strong willingness to translate this research into patient benefit, however at present, there is no mechanism for linking basic genomic research to drug development and clinical trials.

Therefore, there is an urgent need to integrate and strengthen the existing basic and clinical academic research activities with commercial sectors at an European level. The recent call by the EU Network of Excellence to structure clinical research in paediatric oncology in Europe is a positive step, but more action is needed at both EU and Member State levels [9].

The specific policy objectives for paediatric oncology drug development identified by the ITCC consortium^aa^ include the following.

- Prioritisation and selection of anti-cancer compounds developed by pharmaceutical companies for adult use through a comprehensive pre-clinical R&D drug evaluation programme to identify compounds that may be also active in paediatric cancers.
- Identification and validation of drug targets unique to paediatric cancers for therapeutic exploitation.
- Demonstration of proof of concept through mechanistic hypothesis-testing Phase I/II trials of novel agents by establishing a clinical trials network with critical mass (numbers of investigator centres and patients) and access to contemporary technologies.
- Improve information access and ethical aspects of paediatric clinical research for life-threatening diseases. Specifically, such policies should strive to:
  ○ provide fair and equal information access for parents and patients across Europe on clinical research and updated new therapies via internet-based dissemination such as professional sites, Orphanet or other means of communication;
  ○ improve information quality within each trial through guidelines and parents participation in trial design;
  ○ respond to cultural and ethical differences in member states and associate candidate countries by proposing guidelines and solutions to policymakers and institutional entities.
- Training for new clinical investigator centres, which aim to join paediatric drug development for young scientists and physicians, for all member states and associate candidate countries.

#### [V] SWOT analysis

The faculty identified a number of general issues pertinent to public cancer drug development (Table 5.1).

### 5.3.2. Results of the survey of European and USA key opinion leaders

Response rate of the questionnaire was 70.5% with 79 responses, acceptable for survey research. Characteristics of faculty responders to the questionnaire sent out to the senior cancer drug development faculty were analysed (Table 5.2). Apart from the sex ratio and age range, the sample was reasonably balanced in terms of professional status, geographic locality (by region, i.e. Europe or the USA) and area of interest.

Many of the key issues surveyed could not be sub-analyse the response distribution; however, where clear statistical differences in responses were found are shown graphically with the vertical axis representing number of respondents (Figures 5.1–5.16).

#### [I] Investment in cancer drug development

Policies surrounding funding of cancer research are absolutely critical to the public effort and to PPP. To quantify the views of the faculty, we asked three key questions pertaining to the public-private role(s) in funding drug development research.

- Is private sector support for drug development essential?
- Is the current level of national public sector investment adequate?
- Does the public sector have a limited role in cancer drug development?

Results were quite clear that private sector support for drug development was essential (Figure 5.1), as shown by the number of respondents agreeing or agreeing strongly with the question.

Whilst there was strong view that private sector support was essential, there was a clear division between USA and Canadian versus European views regarding adequacy of public funding at national levels, with the former expressing insufficient funding (Figure 5.2). This is at odds with the findings that public funding systems, particularly in the USA, have well funded programmes of support for both pre-clinical and clinical cancer drug development. Clearly, the perception is there is ‘not enough for the job at hand’. Some commentators questioned the role of public funding to support cancer drug development, however, from a key opinion leader perspective (and in line with the pre-survey interviews), they strongly disagree that the public role is only ‘limited’ (Figure 5.3).

#### [II] Environment for cancer drug discovery and development

The environment for drug discovery research was identified as critical to success during the interview phase. Whilst discussion within the pharmaceutical industry has tended to oscillate between the ‘science problem’ and stronger management and productivity (cost, speed and decision making), for the public sector the concerns have been much broader, even generic [10]. In part, this lies with the fact that public sector drug development activity is a far more networked, organic structure with multiple sovereign parties and complicated funding models (‘source to sink’). A further factor underlying this cultural difference, a view that came across strongly in the interviews, is that the public sector had much longer strategic planning time lines. Typically, ‘programmatic’ cycles of five-plus years were presented, rather than shorter time frames reflected by the ‘project’ approach taken by industry.

Faculty considered intellectual environment and sufficient infrastructure support to be the two most critical aspects for success in their drug development enterprises (Figure 5.4). Technology transfer support and formal industry links, whilst generally agreed as useful, were not seen major issues. Our data, as well as other findings from this work, strongly suggest in cancer drug development a ‘two cultures’^bb^ situation exists where only few individuals from either culture makes the transition between the public and private sectors. This is a critical issue when considering PPP, one often given very little attention.

#### [III] Models of public-private partnership

To delve into these cultural issues further, we investigated key factors around developing successful PPP working models. In this particular area, the views of the cancer drug development faculty were much more heterogeneous. The importance of R&D alliances for industry has certainly dramatically grown over the last decade; however, most of the policy studies and commentaries have been focused on PPP. Increasingly, PPP have gained traction both at institutional level (greater commercial outreach by both principle investigators in the public sector and by host institutions themselves) and supra-national level (e.g. Innovative Medicines Initiative and the European and Developing Countries Clinical Trials Partnership) [11].

We asked our faculty the following questions.

- Are financial incentives important for PPP?
- Should private sector support be short-term project based?
- Should nationalisation of parts of the drug development process be considered?
- Is the balance between private and public cancer drug development correct?

Funding is considered ‘mission critical’, however, in response to whether financial incentives were important for PPP, faculty was split – both disagreeing and agreeing that this was important – but with the greatest number actually disagreeing (Figure 5.5).

During interviews, many of the faculty argued both for the role of the private sector in PPP to be principally project focused, but some also suggested the model needed to evolve with greater long term ‘infrastructure’ commitments by industry (Figure 5.6).

A controversial area arising during interviews was regarding balance between the private and public sectors (Figure 5.7). Some of those interviewed suggested that commercial inclusion was detrimental to rational cancer drug development and potentially stifled innovation. In these interviews, the solution tended to focus on greater public sector (particularly governmental) involvement in supporting cancer drug development. Discussions were complicated by numerous drivers for these views, including the need to develop drugs for super-orphan indications and previous histories of difficult working relations with industry. Our enquiry whether cancer drug development should receive greater public support found clear geographic differences in responses.

Whereas European-based faculty were far more neutral/modestly disagreeing with this proposal, responders from the USA and Canada clearly felt greater public control was needed (Figure 5.8). Although we expected this pattern to be replicated in the following enquiry of current public-private balance correctness, we found a wide spread of responses across the spectrum with a non-significant tendency for the USA and Canada to disagree.

Our findings suggest there is still considerable disagreement within the public sector cancer drug development community as to ideal ‘balance’ between private and public sector partnerships. The opportunity to investigate new models and re-frame PPP is clearly needed. Policy has tended to focus on pharmaceutical-biotech alliances, particularly surrounding ‘fallen angels’, as well as partnered and perceived niche products [12]; however, the importance of developing true PPP to improve innovation (knowledge enrichment, spin off markers) and productivity is a largely unexplored area.

#### [IV] Key policy areas for ‘success’ in cancer drug development

What are the key areas for new policy development over the next decade to improve innovation in cancer drug discovery and development? One of the most important areas, particularly in Europe since the introduction of the ‘Clinical Trials’ Directive, is the regulatory environment. The following questions were explored.

- Is the regulatory environment a key area for success?
- How important are reimbursement policies of new cancer drugs to future success?
- How important are supra-national funding initiatives?
- How important are national funding policies from research funding organisations?
- Is institutional support important for success in cancer drug discovery?
- Are technology transfer and/or incentive schemes important policy areas?

With regards to regulatory environment as a key to success, we found substantial splits between the USA, Canada, the Netherlands and the United Kingdom versus continental Europe (Figure 5.9). This may be partially explained by some European countries greater sympathy in including the ‘Clinical Trials’ Directive into national legislation. However, it is clear that for both public and private sector cancer drug development investigators this remains a hugely serious issue.

Reimbursement of new drugs was raised by many of the faculty as a key policy issue (Figure 5.10) and expressed during interviews that if countries or regions failed in their reimbursement policies, then public sector alliances would be damaged. Perhaps unsurprisingly, American faculty were relatively neutral compared with European. The largest positive responses came from the United Kingdom, suggesting it has become an over-dominant public policy issue skewing the focus away from other equally important factors.

Returning to the importance (or not) of public investment policies in cancer drug development, faulty were clear that both supra-national and national policies were essential, with a clearer consensus in the latter case (Figures 5.11 and 5.12).

Since the EDCTP, publication of PPP in neglected diseases report [13], and global initiatives of GAVI and GFATM, it has become widely accepted that public funding is essential to drive innovation mainly due to the lack of commercial viability found by orphan diseases. However, the ‘orphanisation’ of many common cancers through molecular sub-stratification creates the same problem of commercial viability for developing innovative cancer medicines against rarer indications. Indeed, when one considers the rare adult cancers and paediatric oncology, such an environment already exists.

The key environment for public sector drug development research lies within investigators’ host institutions. Whilst the USA has a long history of major federal support for comprehensive cancer centres [14], Europe has lagged behind, although recent initiatives seek to reverse this trend (i.e. cancer centre accreditation by OECI [15], Network of Core Institutions Initiative by EORTC). Increasingly, both host institutions and research funding organisations are offering technology transfer support.

Whilst institutional level support and organisation were seen as critical policy areas, faculty were split on the relative importance of technology transfer support (Figures 5.13 and 5.14). This may reflect a lack of knowledge or reflect stifling versus promotion of true innovation, as exclaimed by many American faculty with the example of the Bayh-Dole Act [16]. A similar view was expressed by a UK Royal Society report viewing IPR as a double-edged sword, both promoting invention and exploitation as well as limiting the free flow of ideas and information [17].

#### [V] Cancer research and development models

Turning full circle, we return to the question on whether current PPP models or indeed cancer drug R&D models are currently appropriate or whether new policies are needed.

- Are new models in PPP needed?
- Are new models for R&D in cancer drug discovery and development needed?

Responses to both these statements found major consensus that both the current R&D models and PPP arrangements require change if cancer drug development is to be successful in the future (Figures 5.15 and 5.16). During interviews, various solutions and options were offered including major supra-national re-organisations, harmonisations and key paradigm changes in the science approach. The core solutions are discussed below.

## 5.4. Discussion and Policy Implications

This section empirically examines the interaction, relationships and perceptions around cancer drug discovery and development between the public and private sector by focusing on the former community. The weaving of both quantitative data from the questionnaire and qualitative data from interviews has provided one of the first major evidence-based studies of public sector cancer drug development. Ideally, we would have had more responders from non-English speaking countries, particularly those countries increasingly engaged in cancer drug development, such as India, Japan and China. In light of our high response rates and commonality of views on many policy issues, our results are likely to be widely applicable and may serve as a benchmark for future high-resolution studies.

### 5.4.1. Public-private partnership models

Models of PPP are currently a key policy area. In the last two decades, there has been a proliferation PPP internationally in health research, primarily developing and transitional countries within Group I diseases (infections, perinatal mortality, etc). Whilst the notion of PPP is not new with such models existing as early as 1969,^cc^ there have been rapid changes in conceptual and operational approaches. These changes have often been driven by perceived or real deficiencies in public health, often including UN sponsorship. Definitions of PPP have varied but with common flavour – ‘a collaborative relationship between entities to work toward shared objectives through a mutually agreed division of labour’ [18] or ‘...a group of allies sharing the gaols, efforts and rewards of a joint undertaking’ [19].

Despite the globalisation of the cancer burden, surprisingly little thought has been given to the nature of international PPP in any domain of cancer research with most of the focus being placed upon carcinogen control and national cancer control programmes. Indeed, such a deficiency is apparent across all aspects of non-communicable diseases. In the domain of cancer drug development, our data from key opinion leaders clearly show both public and private sectors are necessary. Whilst there might be disagreement over the current and ideal balance of public and private sector, what is absolutely clear is new models are urgently needed. An integral part of this finding was the expression by that the overall R&D model for cancer drug development needs changing to reduce attrition rates, increase the rate and sophistication of parallel biomarker development, and to process the vast numbers of combination regimens and indications for the next generation of cancer drugs. Some of our data suggest that in certain geographic regions the appetite for greater public sector involvement in key cancer drug discovery areas is substantial. In particular, the key PPP areas for policy development were:

- strong institutional support and dedicated funding from public research organisations;
- new models increasing freedom to operate for important translational leads within specific projects, via improving support, light touch governance and a substantial decreasing administrative bureaucracy (nationally legislative, private-contractual, public-contractual).
- Partnerships supporting trans-national cooperation and collaboration focused on key cancers, including ‘orphans’ and not commercially attractive cancer.

The need for these new policy approaches was tempered by expressions that these partnerships should be in the ‘public good’, subject to high-quality peer review and fully and publicly disclosable upon completion. Clear organisational policies, guidelines, selection of partners and governance was seen as essential to ensure an appropriate balance of public funding to compete priorities and transparent probity in the conduct of drug development research. Work in other areas of PPP could easily be applied to protect the legitimacy and integrity of such models [20].

### 5.4.2. Investing in cancer drug development

Public funding remains a key area for cancer drug development. Whilst there was acknowledgement that budgetary concerns were putting increasing pressure on both national and philanthropic funding, the faculty expressed a need for debate around the issue of the public funding of cancer drug development research (and in cancer research per se). Both in the USA and Europe, federal funders were considered relatively slow to support public sector involvement in cancer drug discovery, and although there was clear recognition that supra-national initiatives were needed, there was disagreement about how this funding should flow. For example, the European faculty agreed with the identified bottlenecks that had been identified [21], however, remained broadly neutral on whether the IMI was sufficiently resourced to achieve such ambitious aims. Indeed part of the problem identified was the substitutional nature of this funding in many member states. Crucially, and a major difference commercially, faculty viewed the role of public funding as to provide long-term stable infrastructural funding with arms-length governance to allow creative partnerships. Key multi-national, multi-group initiatives aimed at specific barriers in cancer drug development were further considered important areas for new PPP funding initiatives. However, most faculties did not see financial incentives as key drivers in such public-private partnerships, instead commenting that patient outcomes and benefits were the most critical test of drug development irrespective of commercial viability.

Previous research has supported the importance of public sector investment in basic sciences [22], as well as correlated between federal funding and privately funded research [23]; however, the importance of public sector investment for drug development research for specific therapeutic area has not been extensively examined. Our findings strongly support the need for the public sector investment across all areas of cancer research, including drug discovery and development. It is clear there is not a simple linear relationship between the public and private sector investment in drug development research, where the public sector performs the more ‘basic’ aspects and the private sector exploits this. Rather, there is a more complex interplay along the entire development pathway continuing into post-marketing. One critical point made by faculty was a need for both the private sector and public policymakers to appreciate cancer drug development extends past Phase IV and not considered ***fait accompli*** once marketing authorisation is given. Key policy issues are the following.

- Requirement for specific federal programmes aimed at critical scientific hurdles and orphan areas.
- National, and less so supra-national policies, directed at public sectors are considered essential. Currently many countries do not have sufficient public sector support.
- Investment in dedicated training cancer drug development programmes for both clinical and non-clinical faculty.

### 5.4.3. Environment for cancer drug development

Our findings indicate intellectual environment (‘trained drug development faculty embedded in centres with sufficient critical mass’), and infrastructure provisions were considered the most important areas for institutional and national policies. Many faculty commented the time had come to be more rational about which major technologies centres should invest is and which centres they should ‘have’ via external strategic alliances. Many comments focused on the role of federal funding to provide dedicated facilities for clinical development as well as key clinical technologies. Whilst public funding is recognised as essential for proof-of-concept work feeding into downstream product development, national RFO’s and institutions have a broader role in providing dedicated clinical facilities plus specific facilities to support development work in such areas as novel biologicals^dd^. Both Europe and the USA have major supra-national initiatives aimed at drug development (IMI and Critical Path Initiative); however, faculty saw these as lesser priorities than ensuring sound institutional level and national level policies.

A number of countries clearly identified over-regulation and reimbursement of new cancer drugs as critical policy issues. In the case of regulatory impact, there is a clear difference in opinion between the countries that have been subjected to the full force of multiple regulations and those, it appears that have not been. Issues of over-regulation continue to overshadow all aspects of public sector clinical cancer research, remaining one of the greatest future threats for both public and private sectors. Regarding government intervention, some faculty pointed to the additional impact (both negative and positive) of government legislation (in the case of Europe this mostly focused on the impact of Directives and Regulations). Whilst agreeing that a balanced approach [25] was essential, the key policy issue focused on the interface between policymakers and the public sector drug development community, with a clear view that in the battle for the legislative ‘hearts and minds’, the private sector had disproportionate access to and influence on policymakers.

In summary, this paper is a first examination of public sector opinions of cancer drug development. Results show strong opinions for the need of PPP, although conflict on the degree of regulation and public-private balance. What is undisputed is the need for re-examination of cancer R&D models in order to increase efficiency in cancer drug development and ultimately affecting cancer outcomes. New policy approaches are needed, including greater transnational cooperation, support of translational research and degree of institutional involvement. Funding for this new approach and cancer R&D remains problematic, with no clear resolution on best balance of short-versus long-term planning and degree of bureaucracy. Investment in intellectual environment remains important, in addition to examination of regulation bottlenecks. Ultimately, the goal is best cancer outcomes with cooperation the key to improving cancer survival.

## Figures and Tables

**Figure 5.1:** Is private sector support for drug development essential?

**Figure 5.2:** Is the current level of national public sector investment adequate?

**Figure 5.3:** Does the public sector have a limited role in cancer drug development?

**Figure 5.4:** How important is the intellectual (academic faculty) environment?

**Figure 5.5:** Are financial incentives important for public-private partnerships?

**Figure 5.6:** Should private sector support be short-term project based?

**Figure 5.7:** Should nationalisation of parts of the drug development process be considered?

**Figure 5.8:** Is the balance between private and public cancer drug development correct?

**Figure 5.9:** Is the regulatory environment a key area for success?

**Figure 5.10:** How important are policies around the reimbursement of new cancer drugs to future success?

**Figure 5.11:** How important are supra-national funding initiatives?

**Figure 5.12:** How important are national funding policies from research funding organisations?

**Figure 5.13:** Is institutional support important for success in cancer drug discovery?

**Figure 5.14:** Are technology transfer and/or incentive schemes important policy areas?

**Figure 5.15:** Are new models in PPP are needed?

**Figure 5.16:** New models for R&D in cancer drug discovery and development are needed.

**Table 5.1:** Strengths, weaknesses, opportunities and threats for public cancer drug development

***Source:*** The authors.

**Table 5.2:** Demographic characteristics of responses to drug development questionnaire

***Source:*** The authors.

## References

[1] Kaitin KI (2008). Operating models for R&D excellence.

[2] Kola I, Landis J (2004). Can the pharmaceutical industry reduce attrition rates. Nat Reviews Drug Discovery.

[3] Acquadro C, Berzan R (2003). Incorportaing the patients perspective into drug development and communication. Value Health.

[4] Newell DR (2003). Professor Tom Connors and the development of novel cancer therapies by the phase I/II clinical trials committee of Cancer Research UK. BJC.

[5] EU Council Resolution on Paediatric Medicinal Products http://www.nppg.scot.nhs.uk/misc/301y0119.htm.

[6] Official Journal of the European Union (2006).

[7] Hirschfeld S, Ho PT, Smith M, Pazdur R (2003). Regulatory approvals of paediatric oncology drugs: previous experience and new initiatives. J Clin Oncol.

[8] Lashford LS, Thiesse P, Jouvet A (2002). Temozolomide in malignant gliomas of childhood: a United Kingdom Children’s Cancer Study Group and French Society for Pediatric Oncology Intergroup Study. J Clin Oncol.

[9] HEALTH-2010-single-stage. 2.4.1 one network of excellence in this area to be funded to the level of 12M euro

[10] David E, Tramontin T, Zemmel R (2009). Pharmaceutical R&D: the road to positive returns. Nature Reviews Drug Discovery.

[11] European Development Clinical Trials Programme. See http://www.edctp.org/

[12] Czerepak EA, Ryser S (2008). Drug approvals and failures: implications for alliances. Nature Reviews Drug Discovery.

[13] Moran M (2005). A breakthrough in R&D for neglected diseases: new ways to get the drugs we need. PloS Medicine.

[14] Sullivan R (2009). Has the US Cancer Centre model been ‘successful’? Lessons for the European cancer community. Molec Onc.

[15] Saghatchian M (2008). Towards quality, comprehensiveness and excellence. The accreditation project of the Organisation of European Cancer Institutes (OECI). Tumori.

[16] Boettiger S, Bennett AB (2006). Bayh-Dole: if we knew then what we know now. Nature Biotech.

[17] Royal Society Report (2003) Keeping science open: the effects of intellectual property policy on the conduct of science

[18] Partnership for development: proposed actions for the World Bank (Washington DC, World Bank (Discussion paper, Partnerships Group 20 May 1998)

[19] Global Forum for Health Research (1999) Helping to correct the 10/90 gap: an overview. Geneva

[20] Buse K, Waxman A (2001). Public-private health partnerships: a strategy for WHO. Bull World Health Organ [online].

[21] Innovative Medicines Initiative Strategic Research Agenda (July 2005) Creating biomedical R&D leadership for Europe to benefit patients and society

[22] Gambardella A (1992). Competitive Advantage from in-house scientific research: The US pharmaceutical industry in the 1980s. Res Policy.

[23] Ward M, Dranove D (1995). The vertical chain of R&D in the pharmaceutical industry. Econ Inquiry.

[24] Cockburn I, Henderson R (1996). Public-private interaction in pharmaceutical research. Proc Natl Acad Sci USA.

[25] Miller HI, Henderson DR (2007). Governmental influences on drug development: striking a better balance. Nature Drug Discovery.

# Sub article: 6. Supporting and Enabling Innovation in Oncology: Issues in Public Policy

**Published:** 2010-02-03

## 6.1. Background and Objectives

This chapter addresses the question of value of pharmaceutical innovation, particularly in oncology, from a societal perspective and endeavours to address the following questions: first, how do we derive value from innovation; second, is medical innovation in health care worthwhile; third, what approaches are in place to assess the value of (pharmaceutical) innovation, particularly in European countries and, fourth, from a public policy perspective, how do we provide incentives for pharmaceutical innovation particularly in oncology.

Section 2 outlines the methodology employed in this chapter. Section 3 critically appraises the contribution of pharmaceutical innovation to health, health care and well being by drawing on international literature. Section 4 debates whether health and pharmaceutical innovation have been worthwhile from a societal perspective, then discussing evidence on clinical and socioeconomic benefit. Section 5 outlines the different approaches to valuing pharmaceutical innovation in oncology and in Europe in particular and focuses on clinical effectiveness, rate of return regulation and value based pricing. Section 6 discusses available incentives to drive the process of therapeutic innovation forward. Finally, Section 7 draws the main conclusions.

## 6.2. Data and Methods

Both primary and secondary data sources were used to provide the evidence base for this chapter^ee^. The geographical focus of analysis is the European Union, although evidence is drawn from elsewhere, particularly the USA.

Secondary data were acquired by meta-analysis of existing literature on innovation benefits using sources collated from academic databases (primarily ***Medline*** and ***IBSS***) as well as governmental bodies, non-governmental organisations and industry publications. Two phrases entered into search engines: ‘***value of innovation***’ and ‘***pharmaceutical innovation***’, originally producing 1083 and 1008 results, respectively, and subsequently filtered for inclusion and resulted in 60 relevant studies.

Primary data were acquired by means of a questionnaire survey in 15 EU countries (UK, Germany, France, Italy, Spain, the Netherlands, Sweden, Czech Republic, Switzerland, Denmark, Poland, Slovakia, Greece, Finland and Portugal). The survey asked experts in each country to reflect on national policies to assess the value of pharmaceutical innovation, circumstances under which price premia are awarded to new medical technologies, and existence of bias or preference towards certain types of new treatments (e.g. oncology agents) versus others (e.g. new anti-retroviral treatments). The survey tool also distinguished between breakthrough and incremental innovation. Experts included academics and a range of decision makers.

## 6.3. Contribution of Pharmaceutical Innovation to Health and Well Being

### 6.3.1. What is innovation?

The term innovation implies a new way of doing something. It may refer to incremental, radical, and revolutionary changes in thinking, products, processes or organisations [1]. The objective of innovation is positive change, to make someone or something better. In economics the change must increase value, customer value or producer value. Innovation leading to increased productivity is the fundamental source of increasing wealth in an economy.

Similar to the above generic definition of innovation is applied to medical and pharmaceutical innovation, and comprises both radical (breakthrough) and incremental innovation. Health care markets often treat breakthrough innovation differently from incremental innovation, with often processes in place to enable stakeholders to differentiate between these two innovation types. Some systems attempt to adopt a specific framework in order to recognise different types of medicines based on their innovative potential. For example, the US Food and Drug Administration (FDA) distinguishes between ***priority*** review and ***standard*** review drugs. Standard review ‘is applied to a drug that offers at most, only minor improvement over existing marketed therapies’, whereas priority review is applied to ‘drugs that offer major advances in treatment, or provide a treatment where no adequate therapy exists’. Although some studies have attempted to quantify the differential effects of standard versus priority review drugs on health outcomes, only tentatively suggesting a significant effect, this area requires more research to substantiate these claims.

One issue particularly pertinent is incorporation of post-marketing information of drug safety or efficacy and the method for price premium adjustment, if at all, to reflect this new information. This is important given the current debate about ex-ante versus ex-post value assessment (and therefore pricing) in some policy settings, notably the United Kingdom, and perhaps Sweden.

### 6.3.2. Impact of pharmaceutical innovation on health

Over the past 25 years, medical and pharmaceutical innovations, both breakthrough and incremental, have transformed treatment of severe illnesses such as cancer and rheumatoid arthritis, and dramatically improving patients’ lives.

Achieving even incremental innovation requires significant investment and commitment in resources and therefore perceived as a challenge for health care systems. Critics typically site (rising) pharmaceutical spending as a major contributor to spiralling health care costs, suggesting pricing practices and pharmaceutical/biotechnology industry profits are largely to blame for health care budget crises in many countries. Others believe the opposite to be true – rather than being the problem, pharmaceutical innovation on the part of the overall pharmaceutical/biotechnology industry has added significant value to patients, to the economy and to the larger society. In a recent study on the relative importance of medical innovations, clinicians were asked to identify and rank the health care technologies they consider most valuable to themselves and their patients. Pharmaceutical interventions, such as ACE inhibitors, statins, PPIs, H_2_ blockers and inhaled steroids, were all ranked very highly on that list reflecting the therapeutic benefit they deliver [2].

Indeed, the evidence on the value of medical and pharmaceutical innovation and its contribution to health, productivity and return on investment (ROI) is substantial and growing. For example, treatments for heart attacks deliver a $7 return on each $1 of invested in new therapies, such as thrombolytic medicines, stents and long-term drug therapies (1984–1998) [3]. Improved treatments for low birth-weight infants show a $6 return for each $1 incremental investment in new therapies, including special ventilators and artificial surfactants (1950–1990) [3].

Specific classes of drugs, such as statins, have proven to deliver extraordinary value [4] in a number of ways, both for primary and secondary heart disease prevention. Early, statin initiation following an acute heart attack reduces the risk of fatal heart disease or a recurrent heart attack by 24%; every dollar spent on statin therapy in heart attack survivors (versus usual care) has produced health gains valued as high as $9.44. In Type II diabetes patients, statin therapy to lower cholesterol also decreases the risk of coronary events by 25%; every additional dollar spent on statin therapy in Type II diabetics with high cholesterol produces health gains valued at $3. Beta-blockers are another example of high-positive return on investment, as treatment of heart attack patients with beta-blockers show a 35 to 1 return [4]. In cardiovascular disease, GDP gains resulting from increased public/charitable medical research in the UK deliver an additional rate of return ranging from 20%–67%, whilst the internal rate of return from the value of UK net health gains was over 9% [5].

Drug innovation has also transformed the treatment of grievous illnesses, including cancer where significant value has been delivered both at societal and patient levels with the promise of future progress. Research estimates that innovative cancer drugs have increased the 1-year crude cancer survival rate from 69.4% to 76.1%, the 5-year rate from 45.5% to 51.3% and the 10-year rate from 34.2% to 38.1% (1975–1995) [6]. These increases accounted for 50%–60% of the gains in age-adjusted survival rates during the first 6 years after diagnosis, added more than 1 year of life to patients diagnosed with cancer in 1995 and increased the life expectancy of the entire US population by 0.4 years (since lifetime risk of being diagnosed with cancer is roughly 40%) [6]. Further research suggests the value to the patient of a cancer-free life year is actually closer to about $300,000, well above the typical $30,000–75,000 per QALY values used as thresholds in health economic evaluations [7,8].

If society continues to fund further research in cancer therapy, it is likely greater rewards could be expected. Recent studies suggest a cure for cancer might be worth $47 trillion [9], whilst a 1% reduction in cancer mortality worth $500 billion [9,11].

Despite increasing evidence on the impact of pharmaceutical innovation in society, there is concern the value of pharmaceutical innovation may not be fully recognised; there are gaps in measurement, value affected by pricing, or/and reimbursement policies. Metrics, standards and tools to assess drug value are often incomplete and inadequate, as they typically measure and assign a value to life years saved with some estimate of quality adjustment and direct cost offsets, but fail to additionally measure a number of important parameters of interest, such as:

- value to society, not only from the view point of the health care system, but on overall economic productivity;
- value to the patient, the caregiver and also the physician and
- value over time, of the value achieved during the initial treatment period and also of the benefits realised over the duration of the patient’s life (e.g. avoiding costs of future treatments).

Even when a more comprehensive set of metrics is considered, payers and insurers have frequently made explicit decisions to exclude some sources of value (e.g. impact on productivity) from their assessments of the relevant technologies or interventions.

Assessments of value do not take into account the fact that patent protection only allows pharmaceutical and/or biotechnology companies to realise the benefits of their innovation for a fixed period, whilst society enjoys the same benefits in perpetuity. For example, the manufacturer of Prozac enjoyed the financial benefits of this innovation during its effective patent term post-launch, but millions of patients continue to enjoy these benefits today at much reduced (generic) prices. In sum, if all the benefits of drug innovation were appropriately quantified and considered, the value of such innovation over the past three decades would potentially be even larger than initially estimated.

## 6.4. Is Health Care and Pharmaceutical Innovation Worthwhile?

This section discusses the societal benefits of innovation, illustrating how clinical benefits, such as faster recovery (partial or total), higher tolerability, higher survival rate or life expectancy, are more relevant to some therapeutic areas than others, but the socioeconomic benefits gained (e.g. higher productivity) have much in common.

### 6.4.1. Therapeutic/clinical benefits

#### [I] Life expectancy and survival

One of the benefits of pharmaceutical innovation across an ever-increasing range of therapeutic areas is the increased likelihood of an efficacious therapy for any given disease existing which ultimately reduces morbidity and the probability of mortality. Numerous studies have attempted to model the decline in mortality rates for a variety of clinical conditions (e.g. coronary heart disease, diabetes, colorectal cancer) attributable to the introduction of new pharmaceutical therapies over the last 50 years.

Although this link between pharmaceutical innovation and increased life expectancy might seem obvious, econometric analyses have nonetheless been carried out to estimate the quantitative impact of the approval of new drugs on mortality rates from HIV and rare diseases [12]. Though the results of regression analyses support the hypothesis that increased availability of new drugs is associated with a decline in mortality rates, the mechanism by which this might occur is unclear. Some of these clinical benefits potentially contributing to increased life expectancy/decreased mortality are disaggregated in the following sections.

#### [II] Higher probability of faster or full recovery and preventing re-emergence

The link between improved therapeutic/clinical benefits and the development of innovative pharmaceuticals is often preceded by a progression in knowledge of disease aetiology. Chronic myeloid leukaemia (CML) is one example where understanding of the molecular basis at a genetic level led to rational design of a successful targeted therapy. Aided by well-established diagnostic procedures and prognostic factors, the development of Imatinib, a tyrosine kinase inhibitor which helps slow cancer cell proliferation, significantly improved the chances of remission for sufferers of CML, such that 87% of patients now achieve remission [13].

If new treatments show higher probability of faster/full recovery or a higher probability of preventing re-emergence, then payers are typically inclined to award price premia over existing therapies across surveyed countries.

#### [III] Slowing disease progression

In an age where chronic diseases are becoming increasingly prevalent, slowing disease progression is of significant importance. Diabetes, affecting 1.4 million people in the United Kingdom [14] and several million across Europe, is associated with debilitating long-term clinical complications, including retinopathy, peripheral neuropathy, cardiovascular disease, hypertension and nephropathy.

Innovation has affected diabetes therapy through the discovery of glitazones, a group of molecules that increase sensitivity to insulin therapy, revolutionary in non-insulin-dependant diabetes. In an economic evaluation in the UK NHS setting, Beale *et al*. found rosiglitazone combination therapy combined with metformin resulted in better glycaemic control than traditional treatment consisting of metformin and sulphonylurea [15]. Patients on rosiglitazone combination therapy enjoyed a greater life expectancy with an average 123 life years gained per 1000 obese patients. As this treatment delays the onset of insulin therapy, it also results in significant improvement in patients’ quality of life. The reaction of payers to a value proposition promising to slow disease progression and delay or exclude more invasive therapy is also very positive in principle.

#### [IV] Less severe side effects

Adverse side effects can decrease treatment compliance and in the long-term result in higher costs – unused and wasted medication in the UK NHS is currently valued at £100 million annually [16]. This is an important issue in psychiatry where patients’ suitability for community versus institutional care is conditional on medication adherence. Selective serotonin re-uptake inhibitors (SSRIs) used in depression treatments have fewer side effects than traditional tricyclic antidepressants (TCAs). Several studies have shown, despite the price premium awarded to SSRIs, treatment substitutions could be made with virtually no change in overall costs due to reduced readmissions [17]. However, a review assessing the economic benefits of TCAs and SSRIs found although direct medical costs were lower with SSRIs administration, it was difficult to make a definitive evaluation of the overall socioeconomic impact of prescribing SSRIs versus TCAs due to the omission of possible confounding variables [18].

The importance of increasing tolerability is particularly relevant to CNS therapeutics as side effects often have behavioural consequences. For example, third-generation antihistamines developed from active ingredients of second-generation agents, retain the activity of the parent compound but with improved tolerability and pharmacokinetics yet fewer side effects of reduced drowsiness and improved safety.

#### [V] Broader, easier dosing and administration to improve compliance and efficacy

One area in which such incremental innovation occurs is in the development of new drug delivery methods. Clinical benefits of innovative drug delivery forms include a simpler dosing regimen, improved compliance, improved pharmacokinetic profile, increased safety and fewer adverse side effects [19].

To improve compliance, the dosing regimen can be simplified to involve fewer, higher dosage administrations. Methylphenidate, prescribed for attention deficit disorder, can now be administered twice daily, resulting in increased efficacy of the drug whilst reducing the amount of disruption in a child’s school routine.

#### [VI] Quality of life: Greater physical, psychological and social self-sustainability

In some cases, the treatment itself may cause a decrease in quality of life. For example, intravenous chemotherapy treatment is toxic and unpleasant to administer. In the majority of cases improvements in quality of life are recorded. Treatment for multiple myeloma has benefited from recent developments in the production of an oral form of immunomodulatory drugs such as thalidomide benefiting from lower toxicity and increased patient quality of life [20]. Another example of improvement in quality of life is for mental illnesses where discoveries in neuropharmacology have enabled patients to participate in their family and community whilst receiving treatment.

It is often difficult, however, to assess of individual quality of life. This is particularly true with regards to mental health patients as their subjective opinion of their quality of life often diverges from objective bystanders such as caregivers. Consequently, effort in developing new surveys have been made to capture a more accurate quality-of-life assessment to be used for treatment decision making.

### 6.4.2. Socioeconomic benefits

#### [I] Reduced total costs of treatment and non-drug spending

One of the primary socioeconomic benefits potentially accruing to the payer (and society) as a result of innovative therapies is a net reduction in total treatment costs, for example as a result of increased compliance leading to increased efficacy. Kleinke suggested six ‘value propositions’ to categorise innovative pharmaceuticals, depending how costs and benefits of the drug are realised with respect to time and the size of the population (Table 6.1) [21].

In particular, it has been suggested newer drugs prescriptions appear to reduce overall total non-drug medical spending. Evidence gathered from the 1996 US Medical Expenditure Panel Survey (MEPS) found a positive correlation between the age of a drug and the health care spending patients consuming the drug [22]. The study found newer pharmaceutical therapies reduced all types of non-drug health care spending, with the reduction of inpatient spending being most significant; their model suggested that increases of $18 in prescription charges were offset by a reduction of $71.09 in non-drug charges. In line with the notion that newer therapies provide direct economic benefits compared to older therapies, these publications were thought to have important implications for pharmaceutical policy.

However, this set of analyses has been criticised for not accounting whether the medication prescription was concomitant with or post-dated the non-drug health care costs, as this has implications for the direction of causality in the relationship between health care costs, severity of illness and drug age. The studies were also carried out over very limited time periods with little longitudinal data. Controlling for these factors reduces the significance of the results of the Lichtenberg model and brought into question its reliability [23]. Therefore, further work needs to be undertaken in this area, particularly using longitudinal data and analysing policy implementation effects.

#### [II] Costs and outcomes

Any reduced costs have to be considered alongside the change in the delivered clinical benefits, as the relationship between cost-savings and socioeconomic benefits is far from straightforward. In this context, cohort studies from the American Medicare system investigated patient outcomes admitted for hip fracture, colorectal cancer treatment or myocardial infarction across states with different levels of Medicare spending [24]. They found patients in higher spending states received more care, although this did not lead to significantly lower mortality rates, better functional status or better health services satisfaction. What cannot be concluded from this study, however, is whether reducing spending in some areas could have a negative effect on health outcomes. Further research is needed to understand the dynamics of reducing spending without adverse effecting patients, whether this is feasible and in what therapeutic areas.

One problem with properly assessing the cost savings from new therapies is ideal settings found in clinical trials are often not replicated in real life. To this end, Cutler *et al*. carried out an empirical study modelling the effects of hypertensive therapy over the last 40 years using data from the Framingham Heart Study and the US national survey to estimate health outcomes in the absence of innovation in CHD treatments [25]. Although their model had limitations including the inability to control for lifestyle factors associated with CHD such as exercise and sodium intake, their static analysis suggested that US$17.5 billion in costs have been avoided as a result of decreased MI and stroke compared to annual costs of anti-hypertensive medication at $8.8 billion, suggesting a cost-to-benefit ratio in favour of this treatment.

Treatment costs may not always be offset by accrued savings, resulting in government or payer making judgments about the ‘willingness to pay’ threshold. Statin use in the UK NHS is one example, where value avoided costs statin use was calculated at £218 million but not offset by the total statin costs estimated at £3.2 billion. Nonetheless, statins are estimated to have saved approximately 17,400 lives [26].

#### [III] Innovation as a driver of increased costs

Many health policymakers regard innovation, both technological and pharmaceutical, as primary reasons for increased health care costs [3]. Treatment for indications such as myocardial infarction are now more labour, capital and knowledge intensive than 30 years ago. Some argue although many new medical treatments represent genuine medical advances providing novel therapeutic opportunities for patients, there is also a tendency for inappropriate application and over-use. For example, the USA has twice as many MRI machines per capita than Western European countries, yet arguably provides no difference in health outcomes [27]. In this context, Health Technology Assessments (HTA) play an important role in informing decisions about appropriate rate of technology diffusion according to evaluated benefits, drawbacks and cost-effectiveness of an innovative treatment, intervention or technology.

#### [IV] Increased costs and R&D

It is argued that bringing an innovative pharmaceutical product to market, with all associated clinical and socioeconomic benefits, is costly and manufacturer profits are needed to ensure cash flows for future R&D. However, closer examination of American pharmaceutical manufacturers’ gross margins versus their R&D expenditure in a time series analysis reveal the pharmaceutical industry behaves consistently with a rent-seeking model, with industry R&D investments only slightly surpassed by risk-adjusted capital costs. Evidence suggests as firms evaluate potential profit opportunities, they increase R&D investments and may over-invest to almost eradicate any supra-normal profit [28].

Although intense debate exists as to what amounts excessive profits, an issue particularly relevant to the United Kingdom with a profit regulation scheme negotiated between the NHS and the pharmaceutical industry, profits nevertheless act as powerful incentives for innovation. Ideally, innovation could be directed at those areas where significant social returns can be made. One suggested method to achieve this is via the distribution of financial rewards from the health care payer to the innovator themselves, at a value in line with the social value of the (incremental) innovation, thereby aligning social objectives with industry objectives [29]. Although such models face many obstacles in terms of practical implementation, they raise important questions about the way in which the equity and efficiency objectives of both society and the pharmaceutical industry can be achieved simultaneously.

#### [V] Loss of work costs and higher productivity

One important aspect health care payers may consider is the benefit accrued in terms of lower loss of work costs and higher productivity in the work force. Importance of such costs are demonstrated in the example of varicella (chicken pox): from a health care payer’s perspective, routine childhood vaccination would cost $2 per case prevented, $2500 per QALY, whereas alternative calculations accounting for additional loss of work and medical costs find $5 savings per $1 spent vaccinating [30]. Impaired performance is also an issue where an illness does not prevent work attendance but nonetheless affects the individual in their workplace. For example, the indirect costs of migraine to the employer were recently estimated to be US$12 million [31]. Older therapies such as sumatriptan have been demonstrated to be cost-effective, analysis of migraine-related productivity losses in US firms suggested the replacement of standard therapy with newer rizatriptan would result in additional annual savings of US$84-US$118 per employee by avoiding productivity losses [32].

#### [VI] Preventing spread of infectious disease

Prevention of pandemics of infectious diseases, which have debilitating economic effects from workforce productivity plus diminished quality of life, is also an issue to considered. For example, an innovative therapy recently involved in reimbursement decisions in several countries is the human papillomavirus (HPV) vaccine, with demonstrated 98% efficacy against HPV16/18 infection during clinical trials. Infection with strain HPV16/18 is associated with 97% of cervical cancer and without screening programmes found in some countries, there would be several thousand deaths per year from cervical cancer. In the United Kingdom alone, it is estimated an absence of screening would lead to 5000 deaths annually from cervical cancer [33]. Benefits of this new vaccine need to be offset against its modest administration costs to the eligible population – if administered to all females in the United Kingdom under the NHS, it would represent an additional annual outlay of approximately £10 million for the Department of Health [34].

#### [VII] Decreasing resistance

Therapy resistance results in many inefficiencies, due to wasted medications, greater clinician attention required and effecting patient’s quality of life through protracted suffering. Resistance is particularly an issue in antibiotic treatments. Bacterial resistance to antibiotics can be exacerbated by using inappropriate spectrum antibiotics, inadvertently selecting those bacteria harbouring resistance. Developing new generations of antibiotics has been key to increasing the choice of different treatments available, enabling physicians to select the appropriate treatment for a given strain of bacteria. For example, fourth-generation cephalosporins can target a much wider range of gram-positive and gram-negative bacteria than their first-generation counterparts, resulting in a wider range of therapeutic uses.

## 6.5. Different Approaches to Valuing Pharmaceutical Innovation in Europe

### 6.5.1. Valuing innovation in the context of drug reimbursement

Few would disagree that pharmaceutical innovation is worthwhile, although many would argue not all of the features discussed in the previous sections are weighed equally due to differences in opinion across different settings, often dominated by individual value judgments. Indeed, the debate surrounding the value of innovation centres not only around clinical criteria, but also socioeconomic and budgetary. This is done with a view to decide how new products should be reimbursed and whether the decision to reimburse them meet a number of criteria. The type of criteria applied in this context and across study countries vary (Table 6.2), and range from therapeutic assessment of cost-effectiveness and budgetary impacts to cross-country price comparisons.

All countries apply multiple criteria to inform decisions about inclusion of new products in the reimbursement list and the rate at which a new product might be reimbursed. More often than not, valuing an innovative new medicine becomes a function of the price the same medicine commands in neighbouring countries.

Despite the wide range of criteria used explicitly or implicitly to inform pricing and/or reimbursement decisions, explicit strategies used in practice to value pharmaceutical innovation are limited. Evidence suggests that European countries apply three different approaches to valuing pharmaceutical innovation: first, rate of return regulation which, based on its application in the United Kingdom, includes incentives to conduct R&D. Second, assessment of clinical/therapeutic benefit of individual technologies by ranking new treatments based on their therapeutic potential, as is currently undertaken in France and less so in Italy. Third, HTA to inform the relative effectiveness of new treatments. Several countries apply HTA explicitly (the United Kingdom, the Netherlands, Sweden, Switzerland, Finland, Poland), whilst in others HTA plays an increasing role but not used explicitly in decision making. All three approaches are pursued nationally due to national management of health and pharmaceutical care budgets. Supra-national bodies such as the EC have no competence in deciding on allocation or spending of health care budgets and by default have no competence in passing judgment on methodological valuation of new technologies or treatments. The following sections outline each of the three approaches identified and also discuss the role of European institutions in value assessment.

### 6.5.2. Rate-of-return regulation

Rate-of return regulation applies in the case of the United Kingdom, through the Pharmaceutical Price Regulation Scheme (PPRS), which regulates the profits of pharmaceutical companies and, indirectly through profits, the prices of medicines. The scheme has been in operation in the United Kingdom for more than 50 years and administered by the UK Department of Health.

The PPRS objectives are: first, to deliver value for money for the UK NHS by securing the provision of safe and effective medicines at reasonable prices, and to encourage efficient development and competitive supply of medicines; second, to encourage innovation by promoting a strong and profitable pharmaceutical industry capable and willing to invest in sustained R&D for future availability of new and improved medicines benefiting patients and industry; third, to promote access and uptake for new medicines and fourth, to provide stability, sustainability and predictability to help the NHS and industry develop sustainable financial and investment strategies. The scheme applies to branded generics, vaccines, *in vivo* diagnostics, blood products, dialysis fluids, branded products supplied through tendering processes on central or local contracts and biotechnology products.

The relatively free pricing in the United Kingdom – which is, nevertheless, subject to negotiation and moderate reduction in prices each time the scheme is re-negotiated – makes it an attractive market for the launch of new pharmaceutical products. It also provides clear incentives for innovation through the return on capital employed (ROCE) and R&D allowances. A thorough assessment of the true socioeconomic value of innovative pharmaceutical products in the United Kingdom is particularly important, as the United Kingdom is a priceleader in international reference pricing. The countries, which reference prices to the United Kingdom, represent approximately 25% of global pharmaceutical markets (though not all products in each country are referenced to the United Kingdom,) [35].

The scheme has been criticised on several occasions for non-transparency, capital over-investment and for inefficiencies. The divergence between price and value was the main focus of an Office of Fair Trading (OFT) investigation report [35], calling for radical overhaul of the scheme in favour of value-based pricing. The key argument in favour of a new approach from the OFT perspective is that ‘...neither the profit nor the price controls take account of the therapeutic value of drugs. This undermines the extent to which they can help secure value-reflective prices for the NHS. For a scheme that sets out to deliver value for money for the NHS and give companies the right incentives to invest, we consider this to be a significant flaw... the UK is almost unique in the world in not taking explicit account of the therapeutic benefits of drugs in its pricing system’.

The 2009 PPRS will become effective on 1 January 2009 [36], following re-negotiation, and will include several options for patient access schemes, whether financially or outcome-based, the latter implying flexible pricing based on the clinical evidence produced and risk sharing between the Department of Health and individual companies.

### 6.5.3. Assessment of clinical/therapeutic benefit

Our survey has shown all surveyed countries examine available clinical evidence very closely (Table 6.2); however, in two countries it appears the strength of the clinical evidence forms the basis for a classification of drugs as highly innovative, innovative or as me-too.

France applies the principle of therapeutic benefit rendered (Amelioration du Service Medical Rendu, ASMR), where pharmaceutical pricing and reimbursement is decided by negotiations between the pharmaceutical industry and a variety of organisations. The Transparency Committee (CT) initially decides whether or not reimbursement is granted, the scope of indications for which the drug is to be prescribed, and ultimately determines rate of reimbursement (ranging from 30% to 65%). However, prices are determined by the Comité Economique des Produits de Santé (CEPS) based on ‘improvement in medical benefit rating’ (ASMR) or ‘incremental medical benefit’, as judged by the CT (Table 6.3).

The ASMR rating is not the sole deciding factor for determining the final price. Although ASMR 1 to 4 products will be priced higher than their existing alternatives, with ASMR 5 pharmaceuticals not given a price premium compared to existing products, expected sales levels and external reference pricing are also considered. If the new drug is expected to increase the overall sales level in that therapeutic group, a lower price than existing drug may be justified. Overall, CEPS would like to keep prices at or below the EU average.

An Italian algorithm was developed to quantify the value of innovation, in the hope of capturing patients, policymakers and pharmaceutical companies’ perspectives in the innovation process [38]. The Innovation Assessment Algorithm (IAA) had three key aims: first, to take into account and incorporate different properties of drug innovation; second, to provide a numeric weight as a measure of the innovative value of a drug and third, to reassess innovation over time, by incorporating clinical evidence emerging post-marketing authorisation. The final score gives greater weight to earlier ‘efficacy’ branches than effectiveness branches. Whilst several models have been suggested to explicitly quantify innovation in such a manner, this model is significant by identifying innovation aspects in the early-intermediate R&D stage, as well as differentiating between therapeutic innovation, common innovation and industrial innovation (Table 6.4).

Recent evidence suggests analysis of innovation per se is directly influencing policy in Italy. Indeed, a similar hierarchical scheme devised by the AIFA along similar lines, divides pharmaceuticals into three broad categories: those for fatal or serious conditions resulting in permanent disability/hospitalisation, those eliminating or reduce the risk of serious diseases (e.g. hypertensive treatments), and lastly those for ‘non-serious’ diseases such as hay fever. Interestingly, a clear distinction is made between pharmacological innovation (i.e. a new method with necessarily increased efficacy) and technological innovation perhaps not providing therapeutic innovation (Figure 6.1). Ceteris paribus, the former would be rated higher than the latter under their new scheme. AIFA have also stressed the importance of incorporating post-marketing data into their algorithm.

Although the IAA algorithm may appear to have high informational requirements, a similar framework might be useful in structuring the economic information required from pharmaceutical companies to enable greater transparency of health system priorities. Such scientific approaches provide a robust and reliable method of quantifying innovation, and attractive as they simultaneously pacify several stakeholders. The particular model mentioned above also takes into account economic information emerging post-authorisation (ex-post).

### 6.5.4. Health technology assessment in assessing value of innovation

The varying nature and complexity of health technologies, in combination with limited national budgets, has resulted in tensions between delivering cost-effective health care and improving or sustaining a country’s manufacturing and research base. As a result, it has become increasingly important to achieve a balance between affordable health care and the use of innovative health technologies, including pharmaceuticals. Thus, it is necessary to both consider the value (in both medical and economic terms) of a product, plus who benefits from innovation, the optimal usage^ff^, and the appropriate placement in the spectrum of care [40].

The HTA can assist in meeting these challenges by determining which technologies are ineffective versus effective, and define the most appropriate indications for use of the technology [41]. Moreover, HTA can serve to validate a new technology and define its role in health care system. These are important benefits to enable governments to make value-driven decisions concurrently supports innovation, and garners patients and physicians with the information needed to make the best treatment choices [42].

However, the effectiveness of HTA in achieving the above benefits, particularly in terms of encouraging innovation, hinges on properly performed assessments as well as appropriate implementation and use of subsequent recommendations. HTA can encourage innovation if the assessments are properly done and consider a wide range of costs and benefits associated with a new technology, rather than focus solely on acquisition costs. In particular, adoption costs need to be viewed in terms of broader benefits ensued if a technology were integrated into the health system, as budget-driven constraints on the general diffusion of technologies do not necessarily result in the selection of the most effective or cost-effective products. This may require governments to allow additional funding and flexibility between budgets allowing expenditure levels are driven by value, as opposed to arbitrary spending caps [40].

The utility of HTA in encouraging innovation and value-added health care depends on the assessment process, including when and how the review was performed, and resultant decision-making procedures. In particular, the following issues can potentially affect the effective use of HTA in meeting these objectives [39, 43–45].

- Delays in the HTA process can result in deferred reimbursement decisions, restricting patient access to needed treatments.
- Evidence requirements can pose significant hurdles for manufacturers, particularly small, innovative companies, potentially discouraging sponsors from pursuing breakthrough technologies.
- As HTA becomes widespread, assessments occur earlier in the technology diffusion process, which may introduce greater uncertainty in the process and the potential for innovations to appear more or less beneficial than when assessed at latter stages.
- Current assessment methodologies may limit the comparability and transferability across countries and studies.
- Lack of transparency, accountability and stakeholder involvement in the HTA process can decrease the acceptance and implementation of assessment results.
- Limited skilled HTA personnel and international collaboration between review agencies can stymie the efficiency and effectiveness of assessments.
- Separate processes for organisations dedicated to economic assessments, reimbursement/pricing decisions and practice guideline development may hinder the effectiveness and efficacy of the overall decision-making process, leading to unnecessary duplication of efforts and resource use.

In addition, HTAs are more likely to be used by decision makers if policy instruments (e.g. reports, practice guidelines) are available to act on the assessment, and if prior commitments to effectively use the assessments are established. Moreover, as technology cost-effectiveness and patient demand may change with time, periodic review of HTA recommendations are important. To achieve these objectives, greater participation and collaboration among stakeholders, particularly HTA personnel, government officials, industry, health providers and patients, is required. Without adequate input and understanding of the HTA process, stakeholders may mistrust the evidence and subsequent recommendations of the assessment.

Overall, in order for HTA to be of optimal benefit, the assessment process needs to be linked with innovation and other aspects of policymaking. To the latter, it is important that HTA recognise the complexities of decision making, whereby subjective and normative concerns are duly considered. Otherwise, HTA may be limited in its powers to impact upon the policy process and subsequent access to new and effective products. The role of HTA in encouraging innovation and value in health care could be improved by better understanding and addressing the inherent challenges in the HTA process, as outlined below.

The introduction and growth in HTA in Europe parallels an era in health policy that places greater emphases on measurement, accountability, value for money and evidence-based policies and practices. Moreover, the advent of randomised control trials and subsequent availability of data, growth in medical research and information technology and increased decentralisation of health system decision making have all contributed to an increased need for HTA activities [46].

In Europe, the first institutions or organisational bodies dedicated to the evaluation of health care technologies were established in the 1980s, initially at the regional and local level in France and Spain and, later, at the regional level in Sweden in 1987 [43,47]. During the following decade, HTA programmes have been established in almost all countries, either through the provision of new agencies or institutes, or in established academic units or governmental and non-governmental entities (Table 6.5). Broadly speaking, such bodies fall into two general strands: 1) independent (‘arms-length’) review bodies that produce and disseminate assessment reports on a breadth of topics, including health technologies and interventions, and 2) entities under governmental mandate (e.g., from health ministries) with responsibilities for decision making and priority setting, typically pertaining to the reimbursement and pricing of heath technologies. The latter type of HTA body serves either an advisory or regulatory function.

In parallel with establishing HTA entities, many EU countries are investing resources in HTA and associated evaluation activities. For example, Sweden dedicates €5 million/year on the Swedish Council on Technology Assessment in Health Care (SBU) and the Dutch Fund for Investigative Medicine spends €8.6 million/year on health evaluations [41].

### 6.5.5. Role of European institutions

Over the past 3 years, the High Level Pharmaceutical Forum has provided impetus for debate and potential coordination among national pharma policymakers. Prior to its conclusion in 2008, the High Level Pharmaceutical Forum Working Group on Pricing recently submitted a questionnaire to all Member States with the aim of identifying demand-side benefits viewed as important when assessing the value of an innovative medicine [48]. The benefits identified by the Member States fell into three broad categories: therapeutic/clinical benefits, quality-of-life improvements and socioeconomic benefits (Table 6.6).

Despite EU institutions having no competence in health care policy harmonisation, it has been an important achievement to gather the Member States and other stakeholders around the table to understand some of the driving developments at Member State level. More than anything, ‘the Pricing Working Group of the High Level Pharmaceutical Forum has worked out a set of guiding principles which demonstrate that dialogue between Commission, Member States and multiple stakeholders is possible in an attempt to meet the needs of patients, payers and industry alike’ [49].

## 6.6. Fostering Innovation in Oncology: A List of Priorities

In order to foster innovation in oncology, a list of priorities emerge in a number of areas: first, the role of science, research and innovation policy; second, the role of pricing and reimbursement systems in encouraging and rewarding innovation; third, the continuous evaluation of oncology drugs; fourth, the encouragement of long-term innovation and fifth, the optimisation of resource allocation in health care.

### 6.6.1. The role of national and supra-national science, research and innovation policy

#### [I] Guiding government policymaking

The analysis and discussion in Chapters 3, 4 and 5 suggest that a new challenging role is emerging for government, which is both complex and sophisticated, requires scarce resources be more optimally used, and encourages collaboration and shared decision making with other stakeholders (charitable and private sectors), beyond partisanship. This holds in (bio-)medical as well as other areas of research [50].

In an era of globalisation, the role of government in incentivising pharmaceutical R&D in general, and encouraging oncology R&D in particular, is by no means limited, but rather, multi-dimensional and pro-active. Government should encourage private innovation, leveraging investment in innovation to spur economic growth; this can be achieved both directly, through the funding of basic research in key or/and under-researched areas such as rare cancers, or indirectly through the use of market mechanisms such as tax incentives.

The role of government is also significant in basic technology research, where the expectations of long-term public benefit exceed the expectations for private returns to those undertaking the research [51]. The use of collaborative consortia with private co-funding or cost-sharing should not be excluded but altogether encouraged.

More generally, however, a shift in the way that government encourages innovation in the private sector is needed. Direct funding of R&D is a useful tool and can leverage additional resources from the private sector. Yet, encouraging the development of new cancer drugs and the technologies on which they can be delivered probably requires a proactive stance in indirect measures. For instance, prescriptive and coercive regulations have been found to be cumbersome, expensive and inefficient tools for incentivising private investment in technology. It could be the case that output- or performance-based regulations be adopted in this respect.

Tax incentives (e.g. research and development credits) could be further fine-tuned and targeted so as to serve specific objectives, for instance targeting areas of work such as rare diseases and rare cancers. In the same spirit, aspects of intellectual property can be used to encourage innovation, e.g. through enhanced market exclusivity periods.

As creation of knowledge becomes global and their individual funders, whether public, private or charitable, can only fund or have partial access to (new) knowledge development, a new model may be needed in the future where funders of innovation in key areas such as oncology must learn to cooperate as well as compete. Importantly, open access to innovation and knowledge may be needed with society reflecting on its implications (e.g. harnessing the potential of some technology platforms faster) as well as the requirements to achieve it (e.g. re-thinking the global regulatory environment, or intellectual property).

#### [II] Lessons learned from recent initiatives are national and supra-national level

In Europe, the EU Slovakian Presidency in 2008 made headlines with selecting cancer as its main health priority. As a result, an European Partnership for Action Against Cancer was formed (2009–2013), which aims to support Member States and stakeholders (academic, institutions, industry) by creating a framework for identifying and sharing cancer prevention and treatment information, capacity and expertise [52]. This action will be launched end September 2009, and funded until 2013 by the EC in addition to the Research and Development Framework Programme. This action is supported by two other EU level initiatives: the Innovative Medicines Initiative (IMI) and the European Roadmap (ESFRI). The IMI is a public-private partnership for medicines development [53–55] with an additional focus on cancer research, whilst the ESFRI supports clinical trial and bio-banking initiatives.

One of their main goals is cancer research, focusing on translational, transnational and partnered collaborations. Specifically, their aim is to achieve coordination of one third of cancer research from all funding sources by 2013. These goals will be driven by multi-stakeholder working groups undertaking the work directly or, most likely in the case of cancer research, will monitor work done by outside organisations.

The outlined European programmes have undertaken to combat the fragmented state of Europe’s cancer research programmes and convert this variability into a cooperative strength. Although many of these programmes are relatively recent in the history of cancer drug development (European Partnership Action Against Cancer 2009; FP6 2002; FP7 2007; IMI 2007), they are a step towards cohesive cancer research and outcomes. The addition of public-private partnership is encouraging, and will hopefully make Europe more competitive globally.

Furthermore, although EC level cancer research governance and funding appears to be more cohesive than previously, cancer research funding on a charitable level does not. At country level, there is at least one cancer charity per country, many with overall umbrella cancer charities supplemented further by specific cancer charities. Although cohesive data are difficult to come by, significant sums are invested by these organisations in cancer research^gg^. As administrative costs are obviously duplicated by these organisations, this is a further area for investigation to increase cooperation.

Nationally, many countries explicitly support cancer research – the newer members to the EU still are exploring, developing or implementing their cancer plans. To date, all cancer plans support cancer research in Europe, either through their umbrella medical research organisations (i.e. UK Medical Research Council) or via specific cancer organisations (i.e. German Cancer Research Center). Some countries may have more than one organisation supporting cancer research, such as via a medical research programme plus a national cancer programme (i.e. UK, NL), or may have more than one cancer organisation (i.e. France). Thus, it appears, that not only may Europe itself be fragmented, albeit less than before, there may be national fragmentation as well.

On the other hand, some research institutions could be financially neglected if the work they perform is not transnational – very likely in the case of very new, very specialised technology only found in a few locations. The EC initiatives must take care not to neglect these special cases, as they could be sources of breakthrough technology platforms and/or treatments. National and charitable funding may be the only source for these special interest groups; supplementation by public-private partnerships may be an interesting addition with some protectionist provisions for the public institutions involved.

Meanwhile, American cancer research is less fragmented, solely due to its central organisation under the National Cancer Institute (NCI) and its funding is directly approved by the US Congress. Not only does the NCI support molecular research, it also supports translational research. There is a specific Drug Development Platform whose purpose somewhat mirrors the European IMI goal of speedier cancer drug entry into the market.

Furthermore, the NCI has an additional purpose of supporting cancer research occurring in other countries via its International Portfolio. Clinical trials are a large portion of this, but also collection of international experimental medicines protocols and trials, provision of education and expertise, and participation at board level on other cancer research organisations.

The NCI is currently examining its public-private partnership directive, with a proposal that the NCI would be a ‘safe haven’ for encouraging public-private partnerships, outlining issues of intellectual property and any other barriers. Nationally, there is room for expansion in public-private partnership, which the NCI is currently aware of [56]. At the FDA level, there are some projects occurring nationally, only one of which is supporting cancer research [57]. At the state level, there are some public-private partnerships with industry and academia, usually with private hospitals.

Globally, it appears that cancer research with focus on medicines is still relatively in its infancy, with only recently organisations and programmes given focus and direction. Public-private partnerships appear still in their infancy, both in Europe and the US and could perhaps benefit from examining other areas with expertise, such as information technology, which has resulted in major progress. Fragmentation still occurs, particularly on the charitable level, and although this may have negative consequences in terms of administration costs and research duplication by other countries, there may be benefits to highly specialised research areas still at experimental stage.

#### [III] Focus on rare cancers and re-thinking regulation

Rare cancers belong to the group of rare diseases that are normally defined as diseases with a prevalence of less than 50 in 100,000. Even when defined more conservatively by taking into account some peculiarities of natural history and prognosis, rare cancers represent about 20% of all cases of malignant neoplasms, including all cancers affecting children and teenagers and many affecting young adults [59]. There are significant variations in incidence and mortality rates for different types of rare cancers. There are also significant survival differences for the same type of rare cancers between EU member states [60]. In addition to these, there are several problems related to the treatment of rare cancers in Europe, including variability in access to treatment, variability in the availability of information about treatment, lack of medical expertise in the management of rare cancers leading to less than optimal treatment outcomes, potentially higher costs for patients with rare cancers and their families, lack of registries and tissue banks for rare cancers and difficulty in conducting clinical trials.

The recognition of rare cancers as a special case (which, nevertheless, affects a significant proportion of the cancer patient population) and requires multi-dimensional action. In this respect, the ESMO recommendations on stakeholder actions and public policies in rare cancers [60] highlight the multiplicity of actions needed to encourage research and development, access and uptake of new treatments. Such actions relate to reorganising regulation, encouraging research and clinical trials through collaborative actions and networks, calling for consensus guidelines on multi-disciplinary treatment, addressing patient access to care, as well as improving access to information on rare cancers and education of health care professionals.

### 6.6.2. Need for pricing and reimbursement systems to reward and encourage innovation

Health systems worldwide are struggling with containing costs whilst improving patient access and health outcomes. Drug spending has come under particular pressure and scrutiny, despite accounting for only 10%–20% of total system cost and generating significant value. Managing drug spending should be a priority, however, current approaches to containment may have a detrimental impact on patient access and health outcomes: top down price cuts in countries (e.g. Italy) and ‘jumbo grouping’ (e.g. Germany), significant discrepancies in access (e.g. ‘post code prescribing’ in the UK) and, potentially, outcomes (e.g. Cancer survival rates in the UK vs France) and delays in getting innovative drugs to patients as they go through time-consuming HTAs.

A key aspect is timely patient access to innovative, life-saving therapies. Upfront access restrictions stand in the way of this. A more progressive debate could facilitate the global goals of improving patient access and health outcomes whilst managing costs and appropriately rewarding innovation. Pricing and reimbursement systems can fulfil the goal of encouraging innovation, provided they take into account a number of criteria.

The first is timely access, ensuring that patients get timely access to innovative therapies, unfettered by overly restrictive reimbursement, coverage and/or pricing considerations. The second criterion is value-based reimbursement and could be linked to an explicit and objective assessment of incremental value relative to existing standards of care. The third criterion is comprehensive pricing and reimbursement in the sense that the metrics considered when assessing the value of new treatments and setting reimbursement levels should include all elements of value. The fourth criterion relates to flexibility. The total drug benefits and costs to the health system must be assessed over time, by population segment and in a real-life context – with prices/reimbursement levels adjusted as new data on relative value becomes available. Finally, a fifth criterion is collaboration. Payers, providers and manufacturers need to work together, rather than antagonistically, to explore new pragmatic ways of delivering value to the patient constituency. Each of the above criteria is discussed in turn.

#### [I] Timeliness in accessing new and effective treatments

It must be ensured that patients get timely access to innovative new drugs, unfettered by overly restrictive reimbursement/coverage and/or pricing considerations. This is not an easy task, partly because payers’ use of restrictive coverage/reimbursement or pricing policies often interfere with physicians’ ability to make important decisions about individual patient care. Timely access to care can suffer further as many countries have long registration, approval and reimbursement times, serving as a blunt instrument to deny treatment to requiring patients. Finally, the absence of data early on in a new technology’s assessment process is currently taken as evidence of equivalency, heavily weighing assessments against rewarding pharmaceutical innovation.

What policymakers must focus on is enabling ‘fast track’ approval and reimbursement procedures to ensure timely access to innovative drugs (e.g., FDA fast-track process for priority drugs) [61]. Also, establishing processes for conditional reimbursement/pricing decisions, enabling prompt access for drugs whilst real-world market data are collected and analysed is of great importance. In certain cases, launch prices can be negotiated using data from Phase III trials, with agreement to reassess pricing decisions 1 year later when Phase IV trial results become available. Finally, it must be ensured that guidelines and recommendations provide sufficient flexibility for physician discretion in individual cases (e.g. reimbursement with prior authorisation for drugs that might be recommended for a small share of the patient population) to meet potentially atypical patient preferences or clinical circumstances.

#### [II] The role value-based pricing in improving rationality in decision-making

When payers use coverage, reimbursement and/or pricing mechanisms to manage drug spending, they should do so in a value-based way-linked to an explicit and objective assessment of its incremental value relative to existing standards of care.

Current challenges include the following two aspects: first, drug pricing/reimbursement systems do not always base their decisions on an explicit assessment of a drug’s value, often relying heavily on cross-country price comparisons and/or intra-country reference price groups to set prices. Second, pricing/reimbursement systems often struggle with how to measure and explicitly reward drug innovation, with significant variations in the approaches used.

There can be positive responses to these challenges. Where controls are exerted over drug pricing/reimbursement, policymakers could ensure that these are based on mechanisms that explicitly link pricing/reimbursement decisions to an assessment of a drug’s value. Finally, developing explicit mechanisms to appropriately reward valuable innovation via drug pricing/reimbursement (when the drug offers an incremental benefit over the existing standard of care as measured in a comprehensive way) would be an important step.

#### [III] Societal approach and other metrics

When assessing drug value and setting pricing/reimbursement levels, all elements of value, including societal, should be included. However, when value is assessed, pricing/reimbursement systems frequently choose to focus on value exclusively from the health care system (payer) point of view rather than the broader societal or patient/physician (e.g. consider cost offsets to the health care system such as hospital stays and/or other drug costs avoided, but not from increased worker productivity or provider efficiency). Another problem is that most evaluations of drug costs are based on list prices, rather than actual observed net costs in a treatment setting.

It is imperative that standard guidelines for assessing benefits of drugs based on a broader range of applicable metrics are established. These should include humanistic, patient-focused benefits such as quality of life (QoL), longer term direct cost offsets, indirect system costs that might or might not be covered by payers such as worker productivity, and benefits to caregivers as well as patients (e.g. enhanced patient and physician convenience that translates into improved compliance and better outcomes). Furthermore, new standards and tools to more accurately and consistently assess the more challenging metrics may need to be developed (e.g. patient reported outcomes such as QoL).

#### [IV] Risk sharing

Risk sharing refers to a contractual arrangement between a health care payer/provider and a supplier. Traditionally, payers absorb all risks associated with purchasing new medical technologies. In this regard, payers make a decision on whether the technology offers an effective use of their funds, based on the information available at the time of launch. Risk sharing is an attempt to redistribute the balance of risk between the payer and the supplier of the medical technology, and typically involves the supplier of the medical technology providing a ‘guarantee’ relating to the performance of the technology. The guarantee may relate to one or more outcomes of treatment [62]. These could include for instance (a) clinical outcomes, (b) humanistic or quality-of-life outcomes, (c) resource use (e.g. a reduction in hospitalisations), (d) financial outcomes (e.g. a reduction in the amount spent treating a condition) and (e) economic outcomes (e.g. the achievement of a particular cost-effective threshold).

As the pressure on health care budgets intensifies and the cost of some of the more novel treatment remains high, it is likely that the use of risk sharing agreements will intensify in the future. This may be on two fronts: first, in terms of admitting new treatments onto national formularies, and second, in terms of enabling faster uptake. Enabling innovative pricing solutions, for example through pilot schemes making selected innovative medicines available for time-limited periods [63], or through patient-specific franchise schemes, could contribute to faster access and uptake of new therapies. This would have particular application in the case of oncology due to the limited number of patients.

#### [V] Flexible decisions

Total drug benefits and costs to health systems need to be assessed over time and in a real-life context, with prices/reimbursement levels adjusted as new data on relative value becomes available.

In many countries though, value assessments are conducted at drug launch, using only data collected during the treatments’ clinical development process; as new data becomes available, prices need to be allowed to be in line with demonstrated value. Furthermore, the value of a drug and its price is typically set based on the initial indication, with little or no flexibility to evolve it or change it for different indications in which the dosing and/or value over existing therapies could be significantly different. Optimal mechanisms must be determined, in order to allow for drug price/reimbursement variations across different indications/sub-populations based on value propositions.

Pragmatic and viable processes must be created, so that pricing/reimbursement levels are allowed to fluctuate both up and down over time as meaningful new data becomes available, including potential for ‘temporary’ pricing/reimbursement for novel drugs with limited evidence at launch. Finally, it is important to establish appropriate requirements for ongoing data collection on the part of drug manufacturers to provide support for ongoing price/reimbursement adjustments.

#### [VI] Collaboration between stakeholders

Payers, providers and manufacturers must work together, not antagonistically, to establish pilots to investigate new pragmatic ways of managing drug spend, avoiding wholesale ‘top down’ change.

Unfortunately, most pricing/reimbursement systems are not collaborative in their design and operation, with payers often issuing top-down directives, little sharing of data, and assessments made by relatively closed, non-transparent and often political HTA bodies. In addition, drug regulators give little or inconsistent guidance on trial design. Finally, reform often include multiple changes to the current system wholesale, without a clear and open debate of the alternatives and/or productive piloting before roll out.

In order to face these challenges, an inclusive process for defining pragmatic, effective changes to drug approval and pricing/reimbursement approaches must be developed, ensuring these are transparent to all (e.g. manufacturers understand what trials/data will be required for drug approval, reimbursement and pricing decisions well in advance). Pilots to test new approaches to drug pricing/reimbursement must also be established (i.e. in the areas outlined in above points I through IV) before large-scale implementation. Finally, effectively sequencing and staging the rollout of any changes (e.g. begin with a few newly launched drugs and expand as required) rather than attempting to enact wholesale change is an important part of the process.

#### [VII] Minimising externalities

The above will be able to deliver significant benefits if the key negative externalities often associated with the process of enabling access are minimised or altogether eliminated. Such negative externalities often emerge from international price referencing and comparisons, as well as exchange rate differentials and depreciations/appreciations.

### 6.6.3. Continuous evaluation of oncology drugs

Although ex-ante evaluation provides manufacturers with the incentive to invest in gathering the evidence that the health service requires to make approvals and also encourage innovation in areas/therapies where a substantial clinical benefit can be demonstrated, one drawback of the use of ex-ante as opposed to ex-post evidence is that there will be uncertainty surrounding the cost-effectiveness of the treatment outside the RCT setting at the time of launch. Although further ex-post reviews can also be suggested, these may be difficult to ensure as once a pharmaceutical product is approved, the incentive to carry out further trials is diminished and may even be deemed unethical. There is also concern that if ex-post evaluation slips, the evidence-based approach to health systems could be compromised [64]. Nonetheless, a balance between the value of the economic information surrounding the drug and the value of availability of the drug to patients needs to be achieved (as is often emphasised in HTA).

On the other hand, it should be made clear to pharmaceuticals that ex-post evidence is as crucial as ex-ante evidence in proving the value of a new treatment. In order to do this, there needs to be acceptance of data obtained in naturalistic settings, and methodologies on how best to extract value from such data need to be strengthened. Ownership of such data is also important, as the cost associated with gathering evidence is substantial – creating this evidence should provide the scope for collaboration between the payer community and manufacturers.

### 6.6.4. Encouraging long-term innovation

Innovation – whether breakthrough or incremental – can lead to greater subsequent understanding of the aetiology of a disease (i.e. there could be said to be a positive externality from discovery and use of a new drug). The underlying premise in this regard is that knowledge and innovation have a cumulative impact on the understanding of a disease and that impact is often not quantifiable. This may have important dynamic implications for future R&D.

A meaningful pricing system able to deal with both the short- and the long-term implications of and benefits from innovation would probably be complicated to create. Whilst it is often very difficult to anticipate the future gains from innovation and directly incorporate these into a pricing system, governments must encourage such developments to take place. Stratified pricing arrangements such as these seen in France would send a clear message to the research-based industry that innovative products have recognisable value within the pricing system, although, clearly, the starting point vis-à–vis, which ASMR a new medicine represents may be different for different stakeholders. Importantly, there need to be separate (although interconnected) rules targeting value for money and providing incentives to foster long-term innovation.

### 6.6.5. Optimising resource allocation in health care

The way resources are allocated does not necessarily guarantee their optimal use. There may be cases of resource misallocation, which could potentially lead to waste and might also affect access to care. Even if we accept that health care resource allocation operates in silos, evidence shows that even the pharmaceutical budget can be further optimised. Further focus is needed on the demand-side and the behaviour of clinicians, and very importantly, on the use of information systems in real time, both by payers as well as providers. For instance, a recent study found general practitioners performed no better than chance at ranking drugs in 6 therapeutic groups in order of list price [16].

A case where a potentially significant resource re-allocation could take place is the off-patent segment. Despite strong emphasis of European governments in generic medicines over the past two decades, recent evidence suggests that the savings accruing to health insurers from the more intensive use of cheaper generics may have not been realised [65]. There are three dimensions of savings that contribute to the total savings foregone to health insurance from greater use of generics. The first and most obvious dimension reflects the level of generic penetration in a molecule market, post-patent expiry. Generic penetration is a measure of the share of a molecule market that is purchased as generic. Yet, examining the level of generic penetration in some key market, one realises the unfulfilled potential in some of them. Whilst the United Kingdom and Germany lead with 76% and 66% generic penetration, respectively, generic penetration in Spain is 50% in France 33% and in Italy 19%. Italy, France and Spain could realise the largest savings by increasing their generic penetration, although there is significant room for improvement in all study countries for these molecules.

The second dimension of savings that contributes to total foregone savings is the price difference between the originator drug and the generic equivalent. The larger the difference between the two and the smaller the generic penetration, the greater are the foregone savings to health insurance.

Finally, the third dimension that contributes to total foregone savings is the difference between the actual purchased generic price and the lowest generic price. This dimension reflects the degree of efficient purchasing in the generics market itself, independent of generic penetration and originator brand prices. Often this dimension of savings is the most neglected by policymakers, despite the fact that in some cases, the generic price spread may exceed 10 to 1 from highest to lowest. By estimating the effect of additional generic penetration that could occur^hh^ and assuming that health insurers are in a position to procure more cost effectively to the lowest available price on the market, the additional savings to health insurance (or the savings that currently health insurance foregoes) can be calculated. Table 6.7 shows the total savings forgone for five genericised molecules across seven countries: improved genericisation and more efficient purchasing could have saved health insurers over $3 billion in 2004, amounting to a savings on current sales in the order of 43.8%.

This has significant implications for health insurance as it overpays for commodity products that can be acquired more cost effectively, as recent evidence from the Netherlands suggests [66,67]. It also has significant implications for the type of medicines that health insurance is able to finance, given a relatively inelastic budget. Should a resource reallocation occur from the commodity part of the market to the top-end of the market, patient access to needed modern medications could improve in many instances. At the other end of the spectrum, evidence also suggests that the uptake of new cancer drugs is slow and inadequate in many countries and leads to significant inequities in access [68].

The important message from this discussion is that the uptake and diffusion of innovation may require resource reallocation without compromising patient quality of care, but in itself could have positive implications for access, equity and quality. In pursuing this re-allocation where possible, health insurers may be providing a signal and an indirect incentive to innovators to continue their effort.

## 6.7. Concluding Remarks

This chapter has illustrated that innovation in the pharmaceutical sector brings significant tangible benefits to the patient, the health care payer and the economy in terms of clinical and economic effects. It concludes that, from a historical perspective, the socioeconomic and monetary benefits from pharmaceutical innovation are significant as evidence demonstrates, but that the incentives provided by society to continue the process of innovation are often blurred by the frequent policy objective to satisfy reimbursement cost control and inertia. The chapter also concludes that cost-effective considerations, in the context of health technology assessment, are an important factor in determining the value of new medicines, but may need to be supplemented with a broader incentive structure that takes into account not only static (single technology, in the short term), but also dynamic considerations (innovation as a whole over the longer term). Finally, the chapter proposes that in valuing pharmaceutical innovation it is important to consider the context in which such innovation is taking place, together with strategic issues in resource allocation in the entire pharmaceutical value chain and elsewhere in health care.

## Figures and Tables

**Figure 6.1:** Assessing therapeutic innovation in Italy

**Table 6.1:** The six economic categories for innovative medicines

***Source:*** Adapted from [21].

**Table 6.2:** Criteria for assessing new technologies in 15 EU countries (2009)

***Source:*** The authors, based on responses received.

**Table 6.3:** ASMR categories in France

***Source***: [37].

**Table 6.4:** The IAA algorithm

***Source***: [38].

**Table 6.5:** Institutions and advisory bodies responsible for HTA activities in selected EU countries (2008)

***Note:***

1 These are not an exhaustive list of the agencies available in the country.

***Source***: The authors; adapted and enhanced from [43].

**Table 6.6:** The benefits of pharmaceutical innovation^1^

***Note***:

1 Adapted from EC Progress Report.

**Table 6.7:** Generic policies, savings foregone and impact on stakeholders, 2003–2004, seven countries^1^ (based on five off-patent molecules^2^)

***Notes***:

1 United Kingdom, Germany, France, Italy, Spain, Canada, USA.

2 Omeprazole, simvastatin, lisinopril, paroxetine and metformin. Simvastatin was under patent in the USA in 2003 and 2004; therefore, no additional savings can be calculated in this particular case.

***Source***: [65].

## References

[1] Mckeown M (2008). The Truth About Innovation. Pearson / Financial Times.

[2] Fuchs VR, Sox HC (2001). Physicians’ views of the relative importance of thirty medical innovations. Health Aff (Millwood).

[3] Cutler D, McClellan M (2001). Is technological change in medicine worth it?. Health Affairs.

[4] MEDTAP International (2004). The Value of Investment in Health Care, based on: Goldman et al 1988 Costs and effectiveness of routine therapy with long-term betaadrenergic antagonists after acute myocardial infarction. N Engl J Med.

[5] Buxton M (2008). Medical Research: What’s it worth? Estimating the economic benefits from medical research in the UK.

[6] Lichtenberg F (2004). The Expanding Pharmaceutical Arsenal in the War on Cancer. Nat Bur Econ Res.

[7] Hirth R, Chernew M (2000). Willingness to pay for a quality adjusted life year: in search of a standard. Medical Decision Making.

[8] Nadler E, Eckert B, Neumann P (2006). Do Oncologists Believe New Cancer Drugs Offer Good Value. Oncologist.

[9] Murphy K, Topel R (1999). The Economic Value of Medical Research.

[10] Hay J (2006). Where’s the Value in Health Care. Value Health.

[11] Funding First (2000) Exceptional Returns: The Economic Value of America’s Investment in Medical Research

[12] Lichtenberg F (2001). Are the Benefits of Newer Drugs Worth Their Cost? Evidence From the 1996 MEPS: The Newer the drug in use, the less spending on nondrug items. Health Affairs.

[13] Hehlmann R, Hochhaus A, Baccarani M (2007). Chronic myeloid leukaemia. Lancet.

[14] National Audit Office (2001). Tackling Obesity in England [Online]. www.nao.org.uk.

[15] Beale S, Bagust A, Shearer AT (2006). Cost-effectiveness of rosiglitazone combination therapy for the treatment of type 2 diabetes mellitus in the UK. Pharmacoeconomics.

[16] National Audit Office (2007). www.nao.org.uk.

[17] Wan GJ, Crown WH, Berndt ER (2002). Healthcare expenditure in patients treated with venlafaxine or selective serotonin reuptake inhibitors for depression and anxiety. Int J Clin Pract.

[18] Crown WH (2001). Antidepressant selection and economic outcome: a review of methods and studies from clinical practice. Br J Psychiatry.

[19] Wertheimer AI, Santella TM, Finestone AJ (2005). Drug delivery systems improve pharmaceutical profile and facilitate medication adherence. Adv Ther.

[20] Morgan GJ, Krishnan B, Jenner M (2006). Advances in oral therapy for multiple myeloma. Lancet Oncol.

[21] Kleinke JD (2001). The price of progress prescription drugs in the health care market. Health Aff (Millwood).

[22] Lichtenberg FR (1996). Do (more and better) drugs keep people out of hospitals. Am Econ Rev.

[23] Zhang Y, Soumerai SB (2007). Do newer prescription drugs pay for themselves? A reassessment of the evidence. Health Aff (Millwood).

[24] Fisher ES, Wennberg DE, Stukel TA (2003). The implications of regional variations in Medicare spending. Part 2: health outcomes and satisfaction with care. Ann Intern Med.

[25] Cutler DM, Long G, Berndt ER, Royer (2007). The value of antihypertensive drugs: a perspective on medical innovation.Cutler. Health Aff (Millwood).

[26] NERA (2004). www.nera.com.

[27] Bodenheimer T (2005). High and rising health care costs. Part 2: technologic innovation. Ann Intern Med.

[28] Scherer FM (2001). The link between gross profitability and pharmaceutical R&D spending. Health Aff (Millwood).

[29] Hollis A (2005). An efficient reward system for pharmaceutical innovation. www.calgary.edu.

[30] Lieu TA, Cochi SL, Black SB (1994). Cost-effectiveness of a routine varicella vaccination program for US children. JAMA.

[31] Hawkins K, Wang S (2007). Indirect Cost Burden of Migraine in the United States. JOEM.

[32] McCormack PL, Foster RH (2005). Rizatriptan: a pharmacoeconomic review of its use in the acute treatment of migraine. Pharmacoeconomics.

[33] Peto J, Gilham C, Fletcher O (2004). The cervical cancer epidemic that screening has prevented in the UK. Lancet.

[34] Department of Health (2007). www.dh.gov.uk.

[35] Office of Fair Trading (2007). The Pharmaceutical Price Regulation Scheme [Online]. http://www.oft.gov.uk/advice_and_resources/resource_base/market-studies/priceregulation.

[36] Department of Health (2008). The Pharmaceutical Price Regulation Scheme. http://www.dh.gov.uk/en/Publicationsandstatistics/Publications/DH_091825.

[37] Pharmaceutical Pricing and Reimursement Information (2007) France

[38] Caprino L, Russo P (2006). Developing a paradigm of drug innovation: an evaluation algorithm. Drug Discov Today.

[39] Atella V (2008). Valuing innovation in drugs in the Italian NHS personal communication.

[40] Drummond M (2006). Health technology assessment. Has the UK got it right?.

[41] Drummond M (2001). The use of economic evidence by health care decision makers. Eur J Health Econ.

[42] Sorenson C, Drummond M, Kanavos P (2007). Ensuring Value for Money in Health Care: The Role of HTA in the European Union.

[43] Zentner A, Valasco-Garrido M, Busse R (2005). Methods for the comparative evaluation of pharmaceuticals. GMS Health Technol Assess.

[44] Anell A (2004). Priority setting for pharmaceuticals. Eur J Health Econ.

[45] Busse R, Orvain J, Velasco M (2002). Best practice in undertaking and reporting health technology assessments. Int J Health Tech Assess Health Care.

[46] OECD (2003).

[47] Garcia-Altes A, Ondategui-Parra S, Neumann P (2004). Cross-national comparison of technology assessment processes. Int J Health Tech Assess Health Care.

[48] European Commission (2007). http://ec.europa.eu/enterprise/phabiocom/comp_pf_mtng_20070626.htm.

[49] Cueni T (2008). Can Europe afford innovation?. Eurohealth.

[50] Branscomb L, Keller JH (1999). Investing in innovation: Creating a research and innovation policy that works.

[51] Branscomb L, Keller JH, Branscomb L, Keller JH (1999). Towards a Research and Innovation Policy. Investing in innovation: Creating a research and innovation policy that works.

[52] Commission of the European Communities (2009). Action against cancer: European Partnership. Brussels COM(2009).

[53] IMI http://imi.europa.eu/index_en.html.

[54] EFPIA Press Release (2009).

[55] IMI Research Agenda: Creating biomedical R&D leadership for Europe to benefit patients and society. Feb 2008

[56] Niederhuber JE (2009). Facilitating patient-centered cancer research and a era of drug discovery. Oncol.

[57] FDA. Private Public Partnership Programme (2009). http://www.fda.gov/AboutFDA/PartnershipsCollaborations/PublicPrivatePartnershipProgram/ucm166082.htm.

[58] European Society for Medical Oncology (ESMO) (2008). Improving rare cancer care in Europe: Recommendations on stakeholder actions and public policies.

[59] Gatta G (2006). Survival from rare cancer in adults: a population-based study. Lancet Oncol.

[60] European Society for Medical Oncology (ESMO) (2008). Improving rare cancer care in Europe: Recommendations on stakeholder actions and public policies.

[61] Food and Drug Administration, United States of America (2007). http://www.fda.gov/oashi/fast.html.

[62] Espin J (2009). Personal communication.

[63] Office for Life Sciences (OLS) (2009).

[64] Claxton K (2007). OFT, VBP: QED. Health Econ.

[65] Kanavos P, Costa Font J, Seeley E (2008). Competition in generic pharmaceutical markets. Economic Policy.

[66] CVZ (2008). Preference Policy in the Netherlands. www.cz.nl.

[67] Kanavos P, Seeley E, Vandoros S (2009). Tender systems for outpatient pharmaceuticals in the European Union: evidence from the Netherlands Germany and Belgium LSE Health for the EC.

[68] Jonsson B, Wilking N (2007). A global comparison regarding patient access to cancer drugs. Annals Oncol.

# Sub article: 7. Conclusions and Policy Implications

**Published:** 2010-02-03

## 7.1. Introduction

Cancer is becoming of increasing importance in society both on an individual and broader level, with governance playing a growing role. Cancer incidence has been globally increasing steadily over the past half century due to greater longevity, lifestyle (particularly smoking) and environmental influences, as well as improved diagnostics. Cancer mortality is now in most high-income countries the first or second cause of overall death, whilst in middle and low-income countries it is slowly increasing its ranking. Not only is cancer having an economic impact due to its increasing contribution to health care costs, it also is associated with high indirect costs due to the loss of ability to work.

Cancer treatments have improved tremendously over the past three decades and as a result outcomes have improved. The 5-year relative survival rates (1990–1994 diagnosis) for three major cancers average globally for breast cancer 75% (range 40%–82%), prostate 63% (20%–89%) and colorectal 52% (22%–63%). Treatments consist now of surgery, radiotherapy and chemotherapy, either independently or more often in combination, in addition to the newest addition to the arsenal, targeted biological treatments. This latter addition has been proven to be most successful with late stage cancers, which until now have had dismal outcomes, often with less than 10% 5-year survival rates.

Targeted biological treatments are now the newest direction in cancer research and development (R&D) with greatest promise for future oncology drugs. New oncology discoveries have uncovered genomic complexities, finding each tumour has a unique generic code. This complexity is compounded by oncology research methodology where ill, in place of healthy, individuals are test subjects, new molecules are tested in combination with best practice consisting of other treatments, as well as large numbers of cancer sites each with its own genetic fingerprint. As a result, oncology pharmacology R&D has the highest failure rate, particularly during Phase III, and 20% higher costs than other new molecular entities (NME). Despite this failure rate, this is one of the most active portions of total R&D, with new compounds in development phase increase twofold from 2005–2009.

Due to this complex interplay of oncology and pharmacology R&D, this report maps current oncology funding and management of R&D structures in Europe and the USA, examining public-private relationships and current oncology R&D strategies, as a result producing oncology innovation recommendations. Specifically, the objectives of the report are (a) to map current funding and management of oncology R&D via questionnaire and interviews of oncology experts; (b) to produce a high-resolution bibliometric analysis of oncology drug R&D in order to better understand the public-private mix in research activity; (c) to investigate the cumulative life-time funding of specific oncology drugs; (d) to review current public policy affecting oncology drug R&D, specifically, public R&D investment policies, transnational investment policies, regulatory policies, and drug reimbursement policies and (e) to propose future oncology policies supporting the R&D process.

## 7.2. Funding

The report captured both public and private funding of oncology R&D, important in estimating long-term outlook for cancer outcomes and oncology care.

Generally, there are two types of motivation for publicly funded research: economical and political/social reasons. The former helps create new knowledge and new products thus contributing to the wealth of a country, whilst the latter represent a social driver as cancer is a significant and public disease. Both are important together to create new knowledge, new treatments and ultimately improve oncology patient outcomes.

### 7.2.1. Results of public and private research funding organisations survey

Public oncology R&D funding can be sourced from a variety of sources: national governments, regional authorities, charities, non-governmental organisations (NGOs) and supra-national organisations. This funding can be ***directly*** tagged for oncology research from these organisations or ***indirectly*** flow into oncology research via overall budgets (i.e. hospital budgets).

One hundred and fifty three public research funding organisations (RFO) in the EU (UK 19, France 12, Belgium 12, Italy 11) and 21 in the USA were identified satisfying the criterion of greater than €1 million annual oncology R&D spending. The EU RFOs spent collectively €2.79 billion whilst the American RFOs spent €5.8 billion in 2007. However, the EU figure does not include the European Commission investment (through programmes such as FP6, FP7 or IMI) and is likely to be an underestimate. If the estimated annual average spend on cancer research is included, the EU figure is closer to €3 billion.

After the USA, the United Kingdom was the largest investor in oncology R&D (€1.1 billion) followed by Germany (€426 million), France (€389 million) and Italy (€233 million). Accounting for population differences still found the USA and United Kingdom at the top (€19, €18 per capita, respectively) followed by Sweden (€12.1), the Netherlands (€8.8) and Norway (€7.2). Specific oncology drug development had similar absolute leaders (USA €1.67 billion, UK €305 million, France €67 million) and per capita leaders (USA €5.60, UK €5.1, Sweden €2.22).

Although comparing the US and the UK appear to have large funding differences, these are reduced when only the EU 15 are compared, and further still when examining trends, as the EU has increased funding 34.7% from 2004 to 2007 whilst the USA only increased 9.7%. This trend furthers When direct and indirect funding are added together, finding the EU investing 0.011% of GDP, or €3.64 per capita, and the USA 0.018% GDP, or €5.74 per capita.

With the exception of the USA and the United Kingdom who have strong national cancer strategies and national funding, the remaining countries do not appear to have coherent national strategies, rather favouring an ad hoc approach to oncology R&D funding. Examination of the direction of RFOs spending found treatment, including oncology drug development, predominant, ranging from under 10% to over 70% of the RFOs investments.

Although government funding remains the major source of public funding, charitable investment in oncology R&D cannot be ignored. In Europe, this amounted to over €300 million in 2007, and in the USA over €230 million.

Examination of private spending found the 17 top pharmaceutical companies globally spending collectively €3.1 billion in 2004, with 59% sourced from European companies. With regards to public-private enterprises, these are becoming of increasing importance, particularly in Europe. Currently, 68% of oncology drug R&D projects in the USA have joint funding, compared to 57% in the EU and 31% in the rest of the world. This is rapidly changing with redirection of focus.

The European Commission (EC) has since the millennium redirected its focus on oncology R&D, specifically for translational and transnational projects. Its previous Framework Programme (FP) 6 (2002–2006) invested €480 on 108 projects and the current FP7 (2007–2013) has so far invested €265 on 65 projects with more to date. Wider European cooperation, long-term impact and translational research are key to successful funding. The Innovative Medicines Initiative (IMI) is a public-private partnership focusing on speeding up the process of drug discovery to treatment.

The USA’s National Cancer Institute (NCI) has a similar programme, the Drug Development Platform, to speed up drug discovery to marketplace. Overall, the NCI’s oncology investment was $4.83 billion, providing funding to public, private and academic research activities both nationally and internationally. Other countries also invest in oncology R&D, some as molecular and some as translational.

### 7.2.2. Policy implications

Six issues came to light with our oncology public funding survey.

First, there are funding gaps between the USA and Europe and within Europe between countries. European dominating countries are France, Germany, Italy and the United Kingdom in absolute terms, whilst in per capita terms the Netherlands and Sweden are the strongest funders, and the newest European Member states are the weakest. Europe has considerably increased its funding since 2004, reflecting increased political interest, both economically and socially, in oncology research outcomes.

Second, it appears publicly funded research is more likely to support basic rather than applied research, industry supporting the latter.

Third, research funding appears fragmented in Europe, with duplication in some areas and insufficiencies in others. The EC creation of the Framework Programme, with focus on transnational and translational research hopes to rectify this rather than create another layer of bureaucracy.

Fourth, indirect funding and charitable funding are two additional areas of oncology R&D investment. In Europe in particular, these sources can be considerable. Annually in Europe, the main oncology charities invest over €500 million in cancer research, whilst almost half of annual oncology R&D comes from indirect sources such as academia and health care systems. This is significantly greater than invested in the USA, and an often overlooked area of oncology R&D funding.

Fifth, the main investors in private oncology R&D are pharmaceutical companies, whose investment into research has increased steadily over the years with significant portions (7%–12%) earmarked for oncology. Despite this investment, the New Drug Applications have remained flat and less New Chemical Entities have been approved in recent years. This has driven large pharma companies to invest more in in-license start-up biotech companies, as well as pursue defined public-private partnerships (PPP).

Sixth, in oncology in particular, PPP have become more interesting due to the uniqueness of oncology research itself in addition to its complexity. Collaboration of industry with academia can reduce economic risk and smooth the operational process. Many pharma companies have now placed their research centres close to relevant major academic areas. More than half of all oncology research in Europe and the USA is now through PPP, and likely to increase in the future.

## 7.3. Capturing Investment in Oncology Through Research Outputs

The bibliometric analysis was a useful addition to our report, giving us information on research outputs with regards to total output, per country, per cancer site, partnerships and industry investment. This information is useful to pinpoint leaders and opportunities for oncology investment and shows where future breakthroughs may come from in what discipline.

### 7.3.1. Results of the bibliometric survey on oncology research output

Bibliometric analysis of 19 cancer drugs, selected for their treatment success, published in the Web of Science between 1963 to mid-2009 produced 28,752 papers for analysis. Paper outputs rose from 200 annually in 1980 to 2000 annually by 2007–2008. Examination of 15 main oncology research countries found the USA the leader (33%) followed by Japan (10.6%), Italy (7.5%) and the United Kingdom (7.1%).

Although international collaboration is increasing, neighbouring countries still favour each other (USA:Canada, UK:NL). An examination of the 15 countries versus the 19 selected oncology drugs found that countries appear to concentrate on certain drugs and produce less research on others. Furthermore, the oncology research portfolio per country had poor correlation to its internal oncology burden, with the exception of melanoma in Australia. Initially, the USA and Europe dominated oncology research outputs; however, this is changing dramatically with the Rest of World (RoW) beginning to dominate.

The type of oncology research performed changed with time from basic to clinical, although per drug this was not necessarily the case. Different countries produced different types of research (i.e. basic: India, China; clinical: Spain, Greece), with 15% of papers describing phased clinical trials, primarily Phase II.

The presence of 26 leading pharma companies, including the 12 associated with development of the 19 selected drugs, among the addresses of the papers occurred on 1589 papers, or 5.5% of the total. Dominating companies responsible for oncology paper outputs were Aventis (274 papers), AstraZeneca (173) and BristolMyerSquibb (155).

### 7.3.2. Policy implications

The results show that oncology research has increased dramatically over the years, and continues to grow even for older compounds. Main oncology R&D countries are the US, Japan and Europe, although China recently has significantly increased its output, whilst collaborations between countries have remained stable since the 1990s. This latter finding is surprising, as proximity still appears to play a major role in international collaborations, despite the advances in global markets and communication.

Furthermore, it appears the burden of a specific disease is not reflected in its oncology R&D output, either nationally or internationally. In addition, there appears to be a gradual mixing of basic to applied research, which means that policy approaches for translational research must understand and support this. It appears also, that this research has a variety of funding mechanisms supporting it, from industry, government and philanthropic sources. Without the basic underlying research, translational research cannot be completed, thus completely ignoring basic research through funding and policy will have long-term negative impacts.

## 7.4. Public Policy in Oncology Development

A unique aspect to this report is a comprehensive survey of oncology clinicians with regards to their opinions on public policy issues affecting cancer, never before completed. This survey was unique in its elicitation of oncology issues influenced by public policy, with both quantitatively and qualitatively responses, in addition to its international responders.

### 7.4.1. Results of our public policy survey of oncology clinicians

Our survey of leading oncology clinicians globally on public policy issues surrounding oncology R&D yielded a number of interesting results. Respondents felt strongly that PPP were the way of the future; however, how this partnership should best be defined was not clearly resolved. Some felt financial incentives were important, whilst others did not and the length of private support was disputed (from neutral to agree).

Differences in country of origin occurred, with Europeans less agreeable to nationalisation of drug development than Americans and Canadians. Americans felt reimbursement policies were less important to success than the RoW, whilst all agreed that the degree of national public sector investment was inadequate to meet future oncology demands.

Furthermore, it was agreed that the current R&D models were unsufficient for specific oncology needs with re-examination in order. Potential new policies should include greater transnational cooperation, support of translational research and a degree of institutional involvement. Regulatory bottlenecks must be closely examined and quickly resolved to meet future oncology needs. Unresolved was ideal degree of funding by public versus private, in particular to meet long versus short-term goals. Overall, it was clear that continued investment in intellectual public research remains important to meet best cancer outcomes.

### 7.4.2. Policy implications

Despite the globalisation of the cancer burden, surprisingly little thought has been given to the nature of international PPP in any domain of cancer research with most of the focus being placed upon carcinogen control and national cancer control programmes.

In the domain of cancer drug development our data from key opinion leaders clearly shows that both public and private sectors are needed. Whilst there might be disagreement over whether the current balance of public and private sector is correct, what is absolutely clear is that new models for this relationship are urgently needed. An integral part of this finding was that the key opinion leaders were clear that the overall model of R&D in cancer drug development needs to be changed to reduce attrition rates, increase the rate and sophistication of parallel biomarker development, and work on the vast number of combination regimens and indications necessary for the next generation of cancer drugs.

In particular, the key areas for policy development to arise for PPP are:

- Strong institutional support and dedicated streams of funding from public research funding organisations.
- New models that increased the freedom-to-operate in terms of following important translational leads within the context of specific projects. This would be achieved by improved support, light touch governance and a substantial decrease in administrative bureaucracy at every level (national legislative, private-contractual, public-contractual).
- Partnerships that supported trans-national cooperation and collaboration focused on key cancers, including those traditionally viewed as ‘orphan’ and thus not particularly commercially attractive.

The need for these new policy approaches was, however, tempered by a view that these partnerships should be in the ‘public good’, subject to high-quality peer review and upon completion fully and publicly disclosable.

The findings also indicate that the intellectual environment (‘trained drug development faculty embedded in centres with sufficient critical mass’) and infrastructure provision were considered the most important areas for institutional and national policies. Many of the faculty commented that the time had come to be more rational about which major technologies a centre needed to build in and which they should ‘have’ by dint of strategic alliances with other groups.

Whilst public funding has been recognised as essential for proof-of-concept work feeding into downstream product development, there is a clear view that national RFO’s and institutions have a broader role in providing dedicated clinical facilities, as well as specific facilities to support development work in such areas as novel biologicals.

Finally, a number of countries clearly identified over-regulation and reimbursement of new cancer drugs as critical policy issues. The issue of over-regulation continues to overshadow all aspects of public sector clinical cancer research and this remains one of the greatest threats to the future for both public and private sectors.

## 7.5. Fostering Innovation in Oncology: A List of Priorities

In order to foster innovation in oncology, a list of priorities emerge in a number of areas: first, the role of science, research and innovation policy; second, the role of pricing and reimbursement systems in encouraging and rewarding innovation; third, the continuous evaluation of oncology drugs; fourth, the encouragement of long-term innovation and fifth, the optimisation of resource allocation in health care.

### 7.5.1. The role of national and supra-national science, research and innovation policy

#### (I) Guiding government policymaking

In an era of globalisation, the role of government in incentivising pharmaceutical R&D in general and encouraging oncology R&D, in particular, is by no means limited, but, rather, multi-dimensional and pro-active. The active involvement of government can be achieved both directly, through the funding of basic research in key or/and under-researched areas such as rare cancers, or indirectly, through the use of market mechanisms such as tax incentives.

The role of government is also significant in basic technology research, where the expectations of long-term public benefit exceed the expectations for private returns to those undertaking the research. The use of collaborative consortia with private co-funding or cost-sharing should not be excluded but altogether encouraged.

A shift in the way that government encourages innovation in the private sector is needed. Direct funding of R&D is a useful tool and can leverage additional resources from the private sector. Yet, encouraging the development of new cancer drugs and the technologies on which they can be delivered probably requires a proactive stance in indirect measures. For instance, prescriptive and coercive regulations have been found to be cumbersome, expensive and inefficient tools for incentivising private investment in technology. Rather, output- or performance-based regulations could be adopted in this respect.

Tax incentives (e.g. research and development credits) could be further fine-tuned and targeted so as to serve specific objectives, for instance, targeting areas of work such as rare cancers. In the same spirit, aspects of intellectual property can be used to encourage innovation, e.g. through enhanced market exclusivity periods.

As the creation of knowledge becomes global and individual funders of new knowledge creation, whether public, private or charitable, can only but fund or have access to a finite fraction of (new) knowledge development, a new model may be needed in the future, where funders of innovation in key areas such as oncology must learn to cooperate as well as compete. Importantly, open access to innovation and knowledge may be needed and society needs to reflect on the implications of this (e.g. harnessing the potential of some technology platforms faster) as well as the requirements to achieve it (e.g. re-thinking the global regulatory environment, or intellectual property).

#### (II) Lessons learned from recent initiatives at national and supra-national level

Research funding programmes operating at European level have undertaken to combat the fragmented state of Europe’s cancer research programmes, convert this variability into a cooperative strength as well as pay particular attention to translational research. Although many of these programmes are relatively recent in the history of cancer drug development, they are a step towards cohesive cancer research and outcomes. The addition of public-private partnership is encouraging, and will hopefully make Europe more competitive globally.

Although EC level cancer research governance and funding appear to be more cohesive than previously, cancer research funding on a charitable level does not. There is at least one major cancer charity per country, many with overall umbrella cancer charities supplemented further by specific cancer charities. Although cohesive data are difficult to come by, significant sums are invested by these organisations in cancer research. As administrative costs are obviously duplicated by these organisations, this is a further area for potential increase in cooperation.

Nationally, many countries explicitly support cancer research – the newer members to the EU still are exploring, developing or implementing their cancer plans. To date, all cancer plans support cancer research in Europe, either through their umbrella medical research organisations (i.e. UK Medical Research Council) or via specific cancer organisations (i.e. German Cancer Research Center). Some countries may have more than one organisation supporting cancer research, such as via a medical research programme plus a national cancer programme (i.e. UK, NL), or may have more than one cancer organisation (i.e. France). Thus, it appears, that not only may Europe itself be fragmented, albeit less than before, there may be national fragmentation as well.

On the other hand, some research institutions could be financially neglected if the work they perform is not transnational – very likely in the case of very new, very specialised technology only found in a few locations. EU-wide initiatives must take care not to neglect these special cases, as they could be sources of breakthrough technology platforms and/or treatments. National and charitable funding may be the only source for these special interest groups; supplementation by public-private partnerships may be an interesting addition with some protectionist provisions for the public institutions involved.

Meanwhile, American cancer research is less fragmented, solely due to its central organisation under the National Cancer Institute (NCI) and its funding is directly approved by the US Congress, supporting both molecular research and translational research. There is a specific Drug Development Platform whose purpose somewhat mirrors the European IMI goal of speedier cancer drug entry into the market.

Furthermore, the NCI has an additional purpose of supporting cancer research occurring in other countries via its International Portfolio. Clinical trials are a large portion of this, but also collection of international experimental medicines protocols and trials, provision of education and expertise, and participation at board level on other cancer research organisations.

The NCI is currently examining its public-private partnership directive, with a proposal that the NCI would be a ‘safe haven’ for encouraging public-private partnerships, outlining issues of intellectual property and any other barriers.

Globally, it appears that cancer research with focus on medicines is still relatively in its infancy, with only recently organisations and programmes given focus and direction. Public-private partnerships appear still in their infancy, both in Europe and the United States and could perhaps benefit from examining other areas with expertise, such as information technology, which has resulted in major progress. Fragmentation still occurs, particularly at charitable level, and although this may have negative consequences in terms of administration costs and research duplication by other countries, there may be benefits to highly specialised research areas still at experimental stage.

#### (III) Rare cancers, regulation and incentives

Rare cancers, even when defined more conservatively by taking into account some peculiarities of natural history and prognosis, they represent about 20% of all cases of malignant neoplasms, including all cancers affecting children and teenagers and many affecting young adults. There are significant variations in incidence and mortality rates for different types of rare cancers. There are also significant survival differences for the same type of rare cancers between EU member states, as there is variability in access to treatment, the availability of information about treatment, and a lack of medical expertise in the management of rare cancers.

The recognition of rare cancers as a special case requires multi-dimensional action to encourage research and development, access and uptake of new treatments. Such actions relate to re-organising regulation, encouraging research and clinical trials through collaborative actions and networks, calling for consensus guidelines on multi-disciplinary treatment, addressing patient access to care, as well as improving access to information on rare cancers and education of health care professionals.

### 7.5.2. Encouraging and rewarding innovation through the reimbursement system

Health systems worldwide are struggling with containing costs whilst improving patient access and health outcomes. Drug spend has come under particular pressure and scrutiny, despite accounting for only 10%–20% of total system costs. Managing drug spending should be a priority; however, current approaches to containment can be blunt and regressive, with detrimental impact on patient access and, potentially, health outcomes. Pricing and reimbursement systems can fulfil the goal of encouraging innovation, provided they take into account a number of criteria.

The first among them is timely access, ensuring that patients get timely access to innovative therapies, unfettered by overly restrictive reimbursement, coverage and/or pricing considerations. In order to do that, a number of actions can be operationalised: there can be ‘fast track’ approval and reimbursement procedures to ensure timely access to innovative drugs (e.g. FDA fast-track process for priority drugs). In addition, establishing processes for conditional reimbursement/pricing decisions, enabling prompt access for drugs whilst real-world market data are collected and analysed is of great importance. In certain cases, launch prices can be negotiated using data from Phase III trials, with agreement to reassess pricing decisions 1 year later when Phase IV trial results become available. Finally, it must be ensured that guidelines and recommendations provide sufficient flexibility for physician discretion in individual cases (e.g., reimbursement with prior authorisation for drugs that might be recommended for a small share of the patient population) to meet potentially atypical patient preferences or clinical circumstances.

The second criterion is value-based reimbursement. This could be linked to an explicit and objective assessment of incremental value relative to existing standards of care.

The third criterion is comprehensive pricing and reimbursement in the sense that the metrics considered when assessing the value of new treatments and setting reimbursement levels should include all elements of value, including societal aspects.

The fourth criterion relates to flexibility. The total drug benefits and costs to the health system must be assessed over time, by population segment and in a real-life context – with prices/reimbursement levels adjusted as new data on relative value becomes available. A fifth criterion is collaboration. Payers, providers and manufacturers need to work closer together, to explore new pragmatic ways of delivering value to the patient constituency. Finally, it is important that standard guidelines for assessing benefits of drugs based on a broader range of applicable metrics are established. These should include humanistic, patient-focused benefits such as quality of life (QoL); longer term direct cost offsets; indirect system costs that might or might not be covered by payers such as worker productivity; benefits to caregivers as well as patients (e.g. enhanced patient and physician convenience that translates into improved compliance and better outcomes). New standards and tools for more accurately and consistently assessing the more challenging metrics may need to be developed (e.g., patient reported outcomes such as QoL).

### 7.5.3. Risk sharing

Traditionally, payers absorb all risks associated with purchasing new medical technologies. Risk sharing is an attempt to redistribute the balance of risk between the payer and the supplier of the medical technology and typically involves the supplier of the medical technology providing a ‘guarantee’ relating to the performance of the technology. The guarantee may relate to one or more outcomes of treatment. These could include for instance (a) clinical outcomes, (b) humanistic or quality-of-life outcomes, (c) resource use (e.g. a reduction in hospitalisations), (d) financial outcomes (e.g. a reduction in the amount spent treating a condition) and (e) economic outcomes (e.g. the achievement of a particular cost-effectiveness threshold).

As the pressure on health care budgets intensifies and the cost of some of the more novel treatment remains high, it is likely that the use of risk sharing agreements will intensify in the future. This may be on two fronts: first, in terms of admitting new treatments onto national formularies and, second, in terms of enabling faster uptake. Enabling innovative pricing solutions, for example through pilot schemes making selected innovative medicines available for time-limited periods, or through patient-specific franchise schemes, could contribute to faster access and uptake of new therapies. This would have particular application in the case of oncology due to the limited number of patients.

### 7.5.4. Minimising (negative) externalities

Significant benefits can be gained if key negative externalities were minimised or altogether eliminated. Such negative externalities often emerge from international price referencing and comparisons as well as exchange rate differentials and currency depreciations or appreciations.

### 7.5.5. Continuous evaluation of oncology drugs

Although ex-ante evaluation provides manufacturers with the incentive to invest in gathering the evidence that the health service requires to make approvals and also encourage innovation in areas/therapies where a substantial clinical benefit can be demonstrated, one drawback of the use of ex-ante as opposed to ex-post evidence is that there will be uncertainty surrounding the cost-effectiveness of the treatment outside the RCT setting at the time of launch.

Ex-post evidence is, nevertheless, as crucial as ex-ante evidence in proving the value of a new treatment. In order to enable this, there needs to be acceptance of data obtained in naturalistic settings and methodologies on how best to extract value from such data need to be strengthened. Ownership of such data is also important, as the cost associated with gathering such evidence is substantial and creating this evidence should provide the scope for collaboration between the payer community and manufacturers.

### 7.5.6. Optimising resource allocation in health care

The way resources are allocated does not necessarily guarantee their optimal use. There may be cases of resource misallocation, which could potentially lead to waste and might also affect access to care. Even if we accept that health care resource allocation operates in silos, then evidence shows that even the pharmaceutical budget can be further optimised. In order to do this further focus is needed on the demand-side and the behaviour of clinicians, and, very importantly, on the use of information systems in real time, both by payers as well as providers. It is also important to measure the performance of systems in general and of different policies in particular. Any efficiency savings emerging should be reallocated and re-invested with a view to improving health care services and patient’s quality of care.

## References

[1] Coleman MP, Quaresma M, Berrino F (2008). Cancer survival in five continents: a worldwide population-based study. Lancet Oncol.

[2] NCI Addressing the global challenge to cancer.

[3] European Commisson (2008). Cancer Research: projects funded under the 6th Framework Programme.

[4] (2008). IMI Research Agenda: Creating biomedical R&D leadership for Europe to benefit patients and society.

[5] National Institute for Cancer (2008). Annual Report.

[6] Niederhuber JE (2009). Facilitating patient-centered cancer research and a era of drug discovery. Oncol.

[7] IMI http://imi.europa.eu/index_en.html.
